# Effectiveness and safety of acupuncture modalities for overweight and obesity treatment: a systematic review and network meta-analysis of RCTs

**DOI:** 10.3389/fmed.2024.1446515

**Published:** 2024-08-21

**Authors:** Youngjin Kim, Ha-im Park, Hongmin Chu, Hanbit Jin, Jungtae Leem

**Affiliations:** ^1^Wonkwang University Korean Medicine Hospital, Iksan, Republic of Korea; ^2^Wonkwang University Gwangju Korean Medicine Hospital, Gwangju, Republic of Korea; ^3^Mapo Hongik Korean Medicine Clinic, Seoul, Republic of Korea; ^4^Department of Diagnostics, College of Korean Medicine, Wonkwang University, Iksan, Republic of Korea; ^5^Research Center of Traditional Korean Medicine, College of Korean Medicine, Wonkwang University, Iksan, Republic of Korea; ^6^Korean Medicine Clinical Research Institute, Wonkwang University Korean Medicine Hospital, Iksan, Republic of Korea

**Keywords:** acupuncture, overweight, obesity, systematic review, network meta-analysis

## Abstract

**Introduction:**

The effectiveness and safety of acupuncture in the treatment of obesity have not been assessed. This poses a challenge for clinicians who choose to use acupuncture in the treatment of obesity, as they are unable to prioritize this approach based on outcome variables.

**Methods:**

In May 2024, a literature search of five databases was conducted. Only randomized controlled trials evaluating body weight (BW), body mass index, waist circumference (WC), and adverse events in patients with a body mass index (BMI) of 25 or higher for various acupuncture modalities were included. The risk of bias was assessed using the Cochrane risk-of-bias tool for randomized trials, version 2. Pairwise meta-analysis (PMA) and Bayesian network meta-analysis (NMA) were performed using a random effects model for quantitative synthesis.

**Results:**

Fourteen studies (*n* = 868) were included. The included studies evaluated the following acupuncture modalities: electroacupuncture (EA) (*N* = 6), laser acupuncture (LA) (*N* = 2), auricular acupuncture (AA) (*N* = 5), and manual acupuncture (MA) (*N* = 3). The PMA found that adding EA to usual care (UC), compared to UC alone, reduced BW (MD = 2.46, 95% CI = 1.12 to 3.80, *I*^2^ = 58%, REM, *N* = 3, *n* = 157). The NMA of BW showed the following effect sizes for UC alone versus each acupuncture modality combined with UC: LA (MD = 2.09, 95% CI = 0.04 to 3.86), EA (MD = 2.04, 95% CI = 0.88 to 3.50), AA (MD = 1.69, 95% CI = −0.11 to 3.58), and MA (MD = 1.02, 95% CI = −0.82 to 2.94). The probability of each modality being the optimal treatment was evaluated using the surface under the cumulative ranking curve. EA was the most efficacious for BW and BMI, while LA was the most efficacious for WC.

**Discussion:**

EA and LA can effectively complement clinical obesity management. The number of included studies was limited, and publication bias may have occurred, necessitating a cautious interpretation of the results. Furthermore, most studies lasted between six and 12 weeks. Future clinical studies of acupuncture for obesity should include longer follow-up periods.

**Systematic review registration:**

https://www.crd.york.ac.uk/prospero/display_record.php?RecordID=387788, identifier CRD42023387788.

## 1 Introduction

Obesity, a major global health problem, is defined as abnormal or excessive fat accumulation (1). The World Health Organization defines overweight as a body mass index (BMI) of 25 or more and obesity as a BMI of 30 or more (2). The global prevalence of obesity has more than doubled since 1980 (3), affecting approximately 30% of the world’s population (4). The obesity epidemic continues to escalate (5), and based on current trends, it is predicted that up to 50% of the population will be overweight or obese by 2030 (6). Obesity is not just an energy imbalance caused by fat deposits but is associated with a range of health problems. Excessive adipose tissue increases cardiac activity, leading to anatomical changes that adversely affect pulmonary, endocrine, and immune function (7). Obesity impairs vascular homeostasis and leads to vascular dysfunction (8). Furthermore, metabolic syndrome associated with abdominal obesity is associated with a 2-fold increased risk of coronary heart disease and cerebrovascular disease and a 1.5-fold increase in all-cause mortality (9). Therefore, obesity is a risk factor for cardiovascular disease and diabetes (10), and increases the risk of other conditions, including musculoskeletal disorders and certain types of cancer (11). Moreover, obesity has a significant impact on healthcare costs (12). In 2011, obesity accounted for between 0.7 and 2.8 percent of the total healthcare spending in the United States (13). Individuals with obesity incurred healthcare costs that were approximately twice those of individuals with a normal weight (13). Severe obesity can reduce the quality of life (14), leading to negative physical, social, and psychological effects. Given the multifaceted interactions between obesity and many other conditions, treating obesity is essential for enhancing life expectancy and improving health outcomes.

Current management options for obesity include lifestyle modification, pharmacotherapy, and bariatric surgery. The United States Food and Drug Administration has approved several medications, including orlistat, lorcaserin, naltrexone-bupropion, phentermine-topiramate, and liraglutide, for long-term use in the management of obesity. However, recent studies have reported adverse effects of pharmacotherapy, such as an increased risk of cancer associated with lorcaserin use (15), and acute myocardial infarction in patients with cardiovascular disease treated with naltrexone/bupropion combination therapy (16). Bariatric surgery is generally recommended for patients with severe obesity with a body mass index (BMI) of 40 or more or a BMI of 35 or more and at least one obesity-related complication (17). GLP-1 receptor agonists, the most recent class of anti-obesity medications, have been observed to cause weight regain upon discontinuation (18). Furthermore, they are expensive and unavailable for long-term use (18). Studies have shown that appropriate bariatric surgery is associated with reduced complications and favorable cost-benefit ratios in the long term (19). However, short-term complications, such as infection and embolization (20), and long-term complications, such as metabolic disorders including metabolic acidosis and alkalosis, arrhythmias, and hormonal disruption, have been reported (21). Therefore, currently available treatments for obesity have several clinical limitations, including limited use in patients with underlying medical conditions, potential complications, and long-term safety concerns. A clear need exists to explore other effective treatments that can complement existing management strategies.

East Asian traditional medicine employs a diverse array of approaches to treat obesity, including herbal medicine, acupuncture, qigong, and lifestyle therapy (22, 23). The effectiveness of acupuncture in the treatment of obesity has been previously demonstrated (24). There are various modalities of acupuncture used in the treatment of obesity, including the following: Different modalities of acupuncture include electroacupuncture (EA), which uses electrical currents to enhance stimulation; laser acupuncture (LA), which uses low-level laser beams instead of needles; auricular acupuncture (AA), which targets points on the ear; and manual acupuncture (MA), the traditional form of acupuncture. Gao et al. (25) conducted a meta-analysis of 13 studies and reported that EA significantly improved BMI, waist circumference (WC), and waist-hip ratio compared with body acupuncture, moxibustion, and other treatments. Furthermore, Kim et al. (26) conducted a meta-analysis of 27 studies that demonstrated that body acupuncture and moxibustion significantly improved BMI compared to lifestyle modifications (diet, exercise), no treatment, and placebo acupuncture (PA). However, no comparisons were made between these interventions. In addition, Zhang et al. (27) conducted a network meta-analysis of acupuncture treatments for obesity. However, the study did not compare the effectiveness of various acupuncture treatments with usual care (UC) and presented effect sizes as standardized mean differences, which is not an intuitive approach for clinicians.

As discussed above, no studies have compared the effectiveness and safety of different acupuncture modalities for the treatment of obesity when used in combination with UC. For clinicians who have decided to use acupuncture in the treatment of obesity, the lack of evidence poses challenges in terms of the prioritization of treatment based on a given outcome variable. Network meta-analysis is a relatively new research methodology that extends the concept of meta-analysis and allows the ranking of treatment effect sizes through direct and indirect comparisons of multiple treatments (28). This study aimed to compare the effectiveness and safety of various acupuncture treatments for obesity in combination with UC. A network meta-analysis was conducted to provide a basis for selecting the most appropriate acupuncture modality for clinical practice.

## 2 Materials and methods

This study adhered to the Preferred Reporting Items for Systematic Reviews and Meta-Analyses extension statement for reporting systematic reviews that incorporated network meta-analyses of healthcare interventions (29). The protocol for this systematic review was registered in the International Prospective Register of Systematic Reviews (PROSPERO, CRD42023387788).

### 2.1 Search strategy and information source

The initial search was conducted on 4 August 2022, in five English-language electronic databases: Medline (via PubMed), Cochrane Central Register of Controlled Trials (CENTRAL), Excerpta Medica Database (Embase), Allied and Complementary Medicine Database (AMED), and Cumulative Index to Nursing and Allied Health Literature (CINAHL), for articles registered before that date. The second search was performed on 8 May 2024. The search strategy used three categories: “Obesity,” “Acupuncture Modality,” and “Randomized Controlled Trials.” The three categories were combined using the “AND” Boolean operator with keywords specific to each database. The detailed search terms are listed in Supplementary Table 1.

### 2.2 Inclusion and exclusion criteria

#### 2.2.1 Type of study design

The inclusion criteria were randomized controlled trials (RCTs). Other studies, such as case reports, reviews, and meta-analyses, were excluded.

#### 2.2.2 Type of population

The inclusion criteria were studies involving individuals with a BMI of 25 or higher, including simple and complex obesity, regardless of sex or age (30).

#### 2.2.3 Type of experimental group intervention

The selection criteria were as follows: (1) studies that used any of the following interventions in isolation: MA, EA, scalp acupuncture, AA, pharmacopuncture, fire acupuncture, warm acupuncture, acupotomy, acupoint catgut embedding, and LA.

The exclusion criteria were as follows: (1) studies that used acupuncture without intradermal insertion, such as auricular acupressure, and (2) studies that used acupuncture with needles embedded in the body, except for AA and catgut embedding.

Specific acupuncture modalities were defined as follows: (1) MA was defined as acupuncture that was maintained for < 1 h to distinguish it from buried acupuncture; (2) if EA was used, even partially, regardless of the acupuncture point, it was classified as EA; (3) AA was defined as acupuncture that was maintained for a certain amount of time with the needles remaining in place for a certain amount of time; and (4) acupressure was excluded for AA but was included as acupuncture if the author used the term acupuncture.

#### 2.2.4 Type of control group intervention

The studies employed no treatment, lifestyle interventions, Western medicine, or PA as controls. Given that individuals participating in weight loss studies are typically highly motivated (31), the control group interventions were collectively referred to as “UC,” and only PA was categorized separately.

#### 2.2.5 Type of outcome

The primary outcome measure was body weight (BW; unit: kg). The secondary outcome variables were WC (cm) and BMI (kg/m^2^) (32). Additionally, we dichotomized the adverse events (AEs) and dropout rates. The dropout rate was calculated as the number of participants who dropped out compared to the number of participants who were randomized. AEs were calculated as the number of participants who experienced AEs compared to the number of participants who were analyzed.

The study’s selection criteria were as follows: (1) studies that included at least one of the following outcomes: BMI, BW, WC, and AEs as outcomes; (2) studies that reported change data (mean ± standard deviation) for the outcomes.

The specific criteria were as follows: (1) for studies comprising two experimental groups of the same kind of intervention with different doses, we selected and used only one data set from the group with the larger intervention dose; (2) for studies measuring the outcome on multiple occasions throughout the procedure, we employed the final outcome measurement; (3) for studies comprising more than one placebo group, we selected and used only one data set with the more pronounced placebo effect to obtain conservative results; and (4) for studies with incomplete outcome measurements, we requested data from the first or corresponding author by email.

### 2.3 Study selection and data extraction

The titles and abstracts of the retrieved studies were initially reviewed based on the inclusion/exclusion criteria. The full texts of potentially eligible articles were cross-reviewed to make the final selection. The process of selecting and excluding the literature was conducted independently by three researchers (YK, HK, and HJ). In the event of a discrepancy, a consensus was reached through discussions between the researchers. If a consensus could not be reached, another researcher (JL) was consulted for the final selection.

Data extraction was performed independently by two researchers (YK and HK) according to the data template form. The data template comprised the key characteristics of the study (publication year, first author’s country, journal name, and study design), subject information (sample size, sex, and age), intervention details (acupuncture points, stimulation, needle type, treatment duration, frequency, and type of intervention), and outcomes (mean and standard deviation of BW, BMI, WC, and AEs).

### 2.4 Risk of bias assessment

Two researchers (YK and HK) independently assessed the quality of the included studies according to Cochrane ROB 2.0 (33). The risk of bias was evaluated for each study based on five categories: (1) bias arising from the randomization process; (2) bias due to deviations from intended interventions; (3) bias due to missing outcome data; (4) bias in the measurement of outcomes; and (5) bias in the selection of reported outcomes. Each category was rated as low, with a few concerns, or high, according to the degree of bias. Disagreements between the two researchers were resolved through discussion; in cases of unresolved disagreements, a third researcher (JL) was consulted.

### 2.5 Quantitative synthesis

#### 2.5.1 Pairwise meta-analysis (PMA)

PMA was performed to directly compare the effect sizes of each intervention and control groups (34). PMA was performed using Review Manager (Version 5.4, Cochrane Collaboration, Oxford, UK). For continuous variables (BW, BMI, and WC), the mean difference (MD) and 95% confidence interval (CI) were used, and a random-effects model was applied to account for the heterogeneity of the various interventions used in the treatment. For binary variables (AEs and dropout rate), the risk ratio (RR) was used, and a random-effects model was applied. The PMA results were visualized using a forest plot. The I-square value was used to evaluate heterogeneity (35).

#### 2.5.2 Network meta-analysis (NMA)

##### 2.5.2.1 Reviewing the assumptions of network meta-analysis

NMA relies on four assumptions: connectivity, homogeneity, transitivity, and consistency of the network (36). These assumptions must be met to ensure the reliability of the results. Network connectivity was assessed visually using a network plot, which allowed for the examination of direct and indirect connections between studies. We confirmed that at least one closed loop was observed and that every study had at least one connection. Homogeneity between studies was assessed using the I-square test. As the intervention details varied across studies, we used a random-effects model to account for both within- and between-study variability in our statistical analysis. Transitivity and consistency examine whether the results of direct and indirect comparisons are consistent. The statistical test of transitivity was a consistency test, and we used the net-split method to test the transitivity and consistency of each intervention comparison.

##### 2.5.2.2 Setting of network meta-analysis

We conducted a Bayesian NMA using the “gemtc” package (Version 1.0-1)^1^ in R software (version 4.2.1; R Foundation for Statistical Computing, Vienna, Austria) to, directly and indirectly, compare the effects of interventions and controls (37). We used the MD for continuous variables and applied a random-effects model (38). The model employed a Markov chain Monte Carlo (MCMC) simulation, and we ran 200,000 simulations, discarding the initial 5,000 simulations to exclude MCMC bias and taking samples every five simulations. We assessed the model convergence using the Brooks-Gelman-Rubin method and evaluated the model convergence by assessing the degree to which the potential scale reduction factor converged to 1 (39).

Network forest plots were used to visualize the effect sizes of each intervention. Nodes were used to represent various interventions and edges to represent head-to-head comparisons between interventions. Rank plots were used to illustrate the probability of each intervention being ranked according to treatment. The surface under the cumulative ranking curve (SUCRA) (40) was represented based on these plots. A SUCRA value closer to 1 and a higher number indicate that the treatment has a relatively better effect, whereas a value closer to 0 indicates a lower effect of the intervention.

### 2.6 Publication bias

To assess the possibility of publication bias and minor study effects in the network meta-analysis of the main (BW) and secondary outcomes (BMI and WC), we used funnel plots and Egger’s regression tests. A funnel plot was constructed by plotting the effect sizes of the included studies against their standard errors. Asymmetry in the plot suggests the presence of a publication bias or minor study effects (41). Furthermore, to quantitatively assess the asymmetry of the funnel plot, we conducted Egger’s regression test (42). A *p*-value less than 0.10 was considered indicative of significant asymmetry and potential publication bias. Analyses were conducted using the R package “netmeta” (version 2.7-0).^2^

## 3 Results

### 3.1 Study description

**FIGURE 1:**
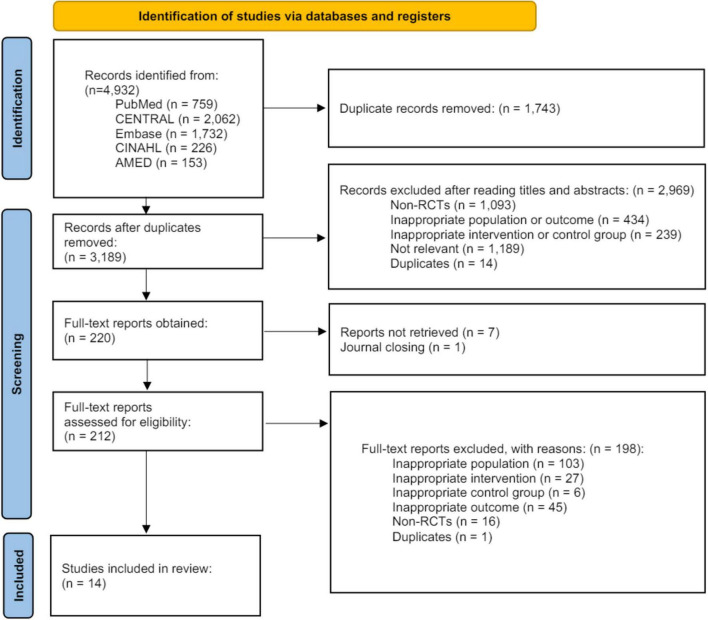
Prisma flow chart. AMED, Allied and Complementary Medicine Database; CENTRAL, Cochrane Central Register of Controlled Trials; CINAHL, Cumulative Index to Nursing and Allied Health Literature; RCT, randomized controlled trial.

A total of 4,932 studies were identified from five English-language databases. After the initial screening of titles and abstracts, 212 studies were reviewed in full text, and 14 RCTs (43–56) with 868 participants were included in the final analysis (Figure 1). Details of the 198 excluded studies are shown in Supplementary Table 2. Of the 14 studies, 11 were in English (43–46, 48–51, 54–56), one was in Chinese (47), and two were in Korean (52, 53). The characteristics of the included studies, including the year of publication, country, age, sex, number of participants, type of intervention and control group, type of outcome, and occurrence of AEs, are summarized in Table 1. Four interventions were used in the included studies: (1) MA+UC; (2) AA+UC; (3) EA+UC; and (4) LA+UC. Of the 14 studies, three included the MA+UC intervention (45, 46, 53), five included AA+UC (44, 45, 49, 50, 54) six included EA+UC (43, 47, 50–52, 55), and two included LA+UC (48, 56). Two control interventions were used in the studies: (1) PA+UC and (2) UC only. Of the 14 studies, nine included PA+UC (44, 46, 48–50, 52–55), and five included UC only (43, 47, 51, 53, 56). Two of the 14 studies were three-arm studies (50, 53) (MA vs. UC vs. PA and EA vs. AA vs. PA), and the other 12 were two-arm studies. Detailed descriptions of the characteristics of the interventions used in these studies, including the acupuncture points, stimulation, needle retention time, and needle type, are summarized in Table 2.

**TABLE 1:** Characteristics of included randomized controlled trials.

Study ID, country	Age (year) Mean (SD)	Initial BMI (*BW) mean (SD)	Sex (M/F or female %)	Number of patients R [T/C (total)] → A [T/C (total)]	Intervention type	Type of control group	Outcome measure	Adverse events (*n*) reported
Hsu et al. (43), Taiwan	Real 40 (11.5) UC 41.3 (9.9)	Real 33.8 (3.4) UC 33.7 (2.9)	100%	24/24 (48) → 22/20 (42)	EA+UC	UC only (exercise)	BW, BMI, WC, HC, WHR, glucose, cholesterol, triglyceride	Real: mild ecchymosis (3), abdominal discomfort (1) UC: none
Kim et al. (53) Korea¶	Real 38.6 (5.7) Sham 37.4 (6.0) UC 39.3 (6.5)	*Real 78.112 (11.503) *Sham 70.246 (7.653) *UC 67.267 (7.917)	100%	UC 18/24 Sham 18 (60) → UC 8/12 Sham 13 (33)	MA+UC (diet)	UC only (diet) PA+UC (diet)	BW, BFP, cholesterol, triglyceride, HDL-C, LDL-C	NR
Hsu et al. (44) Taiwan	Real 40.0 (10.5) Sham 39.4 (13.6)	Real 31.6 (3.9) Sham 31.2 (3.9)	100%	30/30 (60) → 23/22 (45)	AA+UC	PA+UC	BW, BMI, WC, HC, glucose, triglyceride, cholesterol, HDL-C, LDL-C, insulin, leptin, adiponectin, ghrelin, HOMA-IR	Real: minor-inflammation (1), mild tenderness (7) Sham: mild tenderness (2)
Chung et al. (52) Korea	39.6 (11.5)	Real 28.87 (2.15) Sham 28.25 (3.26)	NR	13/13 (26) → 12/11 (23)	EA+UC	PA+UC	BW, BMI, WC, WHR, ASF, BFR, VFA, BULIT-R, KoQoL, BSQ	Real: bluish (6), abdominal discomfort (3) Sham: abdominal discomfort (1), dizziness (1)
Yang et al. (47) China	18–42	*Real 74.58 (7.54) *UC 75.60 (8.31)	7/54	31/30 (61) → 31/30 (61)	EA+UC (diet, exercise)	UC only (diet, exercise)	BW, WHR	NR
Abdi et al. (54) Iran	Real 37.3 (1.0) Sham 38.7 (1.1)	Real 32.15 (0.47) Sham 31.40 (0.41)	NR	102/102 (204) → 86/83 (169)	AA+UC (diet)	PA+UC (diet)	BW, BMI, WC, Body fat, HC, WHR, FBG, TC, triglyceride, HDL-C, LDL-C, anti-HSP27, anti-HSP60, anti-HSP65, anti-HSP70, hs-CRP	None
Abdi et al. (55) Iran	Real 36.9 (8.7) Sham 37.4 (9.1)	Real 32.30 (0.52) Sham 32.74 (0.59)	NR	98/98 (196) → 79/82 (161)	EA+UC (diet)	PA+UC (diet)	BW, BMI, WC, Body fat, HC, WHR, FBG, TC, triglyceride, HDL-C, LDL-C, anti-HSP27, anti-HSP60, anti-HSP65, anti-HSP70, hs-CRP	None
Lien et al. (49) Taiwan	Real 39.2 (11.6) Sham 40.7 (9.7)	Real 29.9 (3.2) Sham 30.6 (4.1)	100%	30/30 (60) → 24/23 (47)	AA+UC (diet)	PA+UC (diet)	BW, BMI, WC, HC, WHR, glucose, triglyceride, cholesterol, HDL-C, LDL-C, adiponectin, insulin, ghrelin, leptin, HOMA-IR, WHO BREF life-quality scores	Real: dizziness (1) Sham: none
Darbandi et al. (50) Iran¶	(EA) Real 38.0 (0.9) Sham 38.0 (1.3); (AA) Real 39.0 (1.8) Sham 37.9 (1.5)	(EA) Real 33.4 (1.3) Sham 33.0 (1.5); (AA) Real 33.4 (2.6) Sham 32.0 (3.9)	0%	20/20 (40) → 20/20 (40);	(EA) EA+UC (diet); (AA) AA+UC (diet)	(EA) PA+UC (diet); (AA) AA+UC (diet)	BMI, WC, HC, TFM	EA: None; AA: None
El-Mekawy et al. (56) Egypt	Real 54.4 (4.4) Sham 52.8 (4.6)	Real 39.91 (3.43) Sham 42.78 (5.27)	100%	14/14 (28) → 14/14 (28)	LA+UC (diet, exercise)	UC only (diet, exercise)	BW, BMI, WC, HC, WHR, TC, HDL-C, LDL-C, TG, FBG, Serum insulin, HOMA-IR	NR
Fogarty et al. (46) Australia	> 18	NR	NR	24/22 (46) → 19/16 (35)	MA+UC (diet, exercise)	PA+UC (diet)	BW, EDRC scale, SF-36v2 health survey, STAI, BDI-2	NR
Tseng et al. (48) Taiwan	Male 42.6 (15.1) Female 37.8 (10.6)	Real 31.2 (5.3) Sham 30.7 (5.6)	11/41 78.85%	26/26 (52) → 26/26 (52)	LA+UC	PA+UC	BMI, WC, HC, WHR, BFP, Appetite sensations (Fullness, Hunger, Satiety, Desire-to-eat, Overall well-being)	None
Yasemin et al. (45) Turkey	(MA) 32.7 (12.3); (AA) 39.1 (7.9)	(MA) 36.6 (6.7); (AA) 38.6 (4.7)	100%	(MA) 25 → 17; (AA) 25 → 21	MA+UC; AA+UC	None	BW, BMI, WC, HC, BFP	MA: Allergic rash (1); AA: None
Ni et al. (51) China	Real 30.79 (8.8) UC 30.50 (9.53)	Real 30.37 (4.75) UC 31.12 (4.94)	Trial 11/17 Control 11/15	30/30 (60) → 28/26 (54)	EA+UC (diet, exercise)	UC only (diet, exercise)	BW, BMI, WC, WHR, FPG, FIN, ISI, HOMA-IR	NR

¶Study with three arms. A, Analyzed; AA, auricular acupuncture; ASF, thickness of abdominal subcutaneous fat; BDI-2, Becks depression inventory; BFP, body fat percentage; BFR, body fat ratio; BMI, body mass index; BSQ, body shape questionnaire; BULIT-R, Bulimia test revised; BW, body weight; C, control; EA, electroacupuncture; EDRC, eating disorder risk composite; F, female; FBG, fasting blood glucose; FIN, fasting insulin; FPG, fasting plasma insulin; HC, hip circumference; HDL-C, high-density lipoprotein cholesterol; HOMA-IR, homeostasis model assessment for insulin resistance; hs-CRP, high sensitivity C-reactive protein; HSP, heat shock protein; ISI, insulin sensitivity index; KoQoL, Korean obesity of QoL; LA, laser acupuncture; LDL-C, low-density lipoprotein cholesterol; M, male; MA, manual acupuncture; NR, not reported; PA, placebo acupuncture; R, randomized; STAI, state-trait anxiety inventory; T, treatment; TC, total cholesterol; TFM, trunk fat mass; UC, usual care; VFA, visceral fat area; WC, waist circumference; WHR, waist/hip ratio.

*Body weight.

**TABLE 2:** Details of the included acupuncture and related therapies.

Study ID, country	Acupuncture points	Stimulation	Needle retention time	Needle type (width, length)	Treatment sessions	Frequency (period)
Hsu et al. (43) Taiwan	CV6, CV9, ST28, KI14, ST26, ST40, SP6	(Needles in the lower legs) Rotated back and forth until the subjects had the sensation of Deqi; (Needles in the abdomen) Intensity: 500 Ω (12–23 V) Frequency: 42 Hz Dense-disperse wave	40 min	Length 3.8 cm	12	2 per wk (6 wks)
Kim et al. (53) Korea¶	LR1, SP1, LU8, SP5	1 stimulation per treatment; Rotated back and forth until the subjects had the sensation of Deqi; Applied pressure to LU8 1 min when waking up at home, right before a meal, eating a snack, or drinking tea	30 min	0.20 mm × 1.5 mm	12	3 per wk (4 wks)
Hsu et al. (44) Taiwan	Hunger, Shenmen, Stomach, Endocrine	One ear per treatment 6 treatments for the two ears in 12 sessions	3 days	Length 2 mm	12	2 per wk (6 wks)
Chung et al. (52) Korea	CV12, CV6, ST25, SP15, SP14; One side of LI4, LI11, ST36, ST44 (M: left side, F: right side)*	Rotated in LI4, LI11, ST36, ST44*; Intensity: 0.27–1.3 mA Frequency: 24 Hz Continuous wave	30 min	0.4 mm × 75 mm	10	2 per wk (5 wks)
Yang et al. (47) China	CV12, ST25, CV4, ST36, ST40, SP9, SP6, BL20, BL21, Ashixue	G6805-2A electroacupuncture machine A level middle, B level 3/4	30 min	NR	15	1 per day (7 wks) 3 days rest between every session
Abdi et al. (54) Iran	Shen Men, Stomach, Hunger point, Mouth, Center of the ear, Sanjiao	Inserted the ear-pressing plaster with seed into the acupuncture points	3 days	NR	12	2 per wk (6 wks)
Abdi et al. (55) Iran	ST25, GB28, CV12, CV9, CV4, SP6 LI 11, ST40 for the excess syndrome (patients with higher energy)*; CV6, SP9 for deficiency syndrome (patients with lower energy)*	Rotated back and forth until the subjects had the sensation of Deqi; Intensity: 500 Ω (12–23 V) Frequency: 30–40 Hz Dense-disperse wave	20 min	Length 3.8 cm	12	2 per wk (6 wks)
Lien et al. (49) Taiwan	Shenmen, Stomach, Hunger, Endocrine	One ear treatment 6 treatments for the two ears in 12 sessions	Remained until next treatment visit	1 cm × 20 mm	12	3 per wk (4 wks)
Darbandi et al. (50) Iran¶	(EA) ST25, GB28, CV12, CV9, CV4, SP6; LI11, ST40 for excess syndrome (patients with higher energy)*; CV6, SP9 for deficiency syndrome (patients with lower energy);* (AA) Shenmen, Stomach, Hungry point, Mouth, Center of the ear, Sanjiao	(EA) Intensity: 500 Ω (12–23 V) Frequency: 30–40 Hz Dense-disperse wave; (AA) Inserted the ear-pressing plaster with the seed into the acupuncture points	(EA) 20 min’; (AA) 3 days	(EA) Length 3.8 cm; (AA) NR	12	2 per wk (6 wks)
El-Mekawy et al. (56) Egypt	CV4, CV9, CV12, ST25, ST36, SP6, ST40	Power output 5 mW Pulse frequency 5,000 Hz Pulse radiation of 200 ns Energy density 2 J/cm^2^	2 min	Wavelength 904 nm	36	3 per wk (12 wks)
Fogarty et al. (46) Australia	LI4, LI11, ST36, ST44, LR3, Shenmen, Hungry, Stomach	Gentle lift/thrust and rotation (not in those with eating concerns)	30 min	(MA) 0.20 mm × 40 mm / 0.25 mm × 40 mm (AA) 0.16 mm × 10 mm	12	2 per wk (6 wks)
Tseng et al. (48) Taiwan	ST25, ST36, ST40, ST44, LI4, LI11, SP6, PC6	Maximum power output 150 mW Power density 0.417 W/cm^2^ Energy density 4 J/cm^2^	10 sec	Wavelength 808 m	24	3 per wk (8 wks)
Yasemin et al. (45) Turkey	(MA) LI4, LI11, ST25, ST36, SP6, SP9, CV12, CV6; (AA) Anti-aggression, Stomach	(MA) 1 stimulation per treatment; (AA) Both sides for auricular acupuncture Stimulated 3 times a day for 30 s	(MA) 30 min; (AA) 15 days (Replaced every 15 days)	(MA) 0.25 mm × 25 mm; (AA) 0.22 mm × 1.3 mm	(MA) 24; (AA) 6	(MA) 2 per wk (12 wks); (AA) 1 per 2 wk (12 wks)
Ni et al. (51) China	BL21, BL20, BL25, BL27, EX-B3, CV12, LR13, ST25, CV4, CV6, ST36, ST40, SP6	Intensity: 1–10 mA Frequency: 2/100 Hz Disperse-dense wave	30 min	Back-shu points: 0.30 mm × 40 mm Front-mu points: 0.30 mm × 40 mm / 0.30 mm × 50 mm	36	3 per wk (12 wks)

*Individualized care according to traditional medicine theory.

¶Study with three arms. AA, auricular acupuncture; EA, electroacupuncture; MA, manual acupuncture; NR, not reported.

### 3.2 Risk of bias assessment

In the deviations from intended interventions category, two studies (51, 54) received a rating of “some concern” and three studies (44, 45, 49) received a rating of “high.” The three studies were rated as “high” due to some participants discontinuing the intervention or dropping out because of treatment ineffectiveness without providing a detailed explanation. All studies received a rating of “low” for the measurement of the outcome category. Overall, the risk of bias was assessed and observed to be low in four studies, “some concern” in seven studies, and “high risk” of bias in three studies (Supplementary Figure 1).

### 3.3 Pairwise meta-analysis

#### 3.3.1 Pairwise meta-analysis on BW

The PMA was performed to assess the effectiveness of various acupuncture treatments in combination with UC versus UC alone on BW. The difference was significant for EA+UC versus UC alone (MD = 2.46, 95% CI = 1.12 to 3.80). For AA, no studies with this design were performed. In addition, various acupuncture treatments in combination with UC versus PA combining with UC for BW showed significance for LA+UC vs. PA+UC (MD = 1.90, 95% CI = 1.25 to 2.55), and one study was included (Supplementary Figures 2a, b).

#### 3.3.2 Pairwise meta-analysis on BMI

A PMA was conducted to assess the effectiveness of various acupuncture treatments in combination with UC versus UC alone on BMI. The difference was significant for EA+UC versus UC alone (MD = 1.50, 95% CI = 0.77 to 2.22). For MA and AA, no studies with this design were performed. In addition, various acupuncture treatments in combination with UC versus PA combining with UC for BMI showed significance for EA+UC vs. PA+UC (MD = 0.37, 95% CI = 0.10 to 0.63), AA+UC vs. PA+UC (MD = 0.34, 95% CI = 0.18 to 0.51), and LA+UC vs. PA+UC (MD = 0.77, 95% CI = 0.51 to 1.03). For LA, only one study was included; for MA, no study with this design was conducted (Supplementary Figures 2c, d).

#### 3.3.3 Pairwise meta-analysis on WC

A PMA was performed to assess the effectiveness of various acupuncture treatments in combination with UC versus UC alone for the treatment of WC. The difference was significant for LA+UC vs. UC alone (MD = 5.35, 95% CI = 3.27 to 7.43). One study was included for LA. For MA and AA, no studies with this design were conducted. In addition, various acupuncture treatments combined with UC versus PA combined with UC for WC were significant for EA+UC vs. PA+UC (MD = 3.56, 95% CI = 0.74 to 6.39). For LA, one study was included, and for MA, no study with this design was performed (Supplementary Figures 2e, f).

### 3.4 Network meta-analysis

#### 3.4.1 Assumption of NMA

**FIGURE 2:**
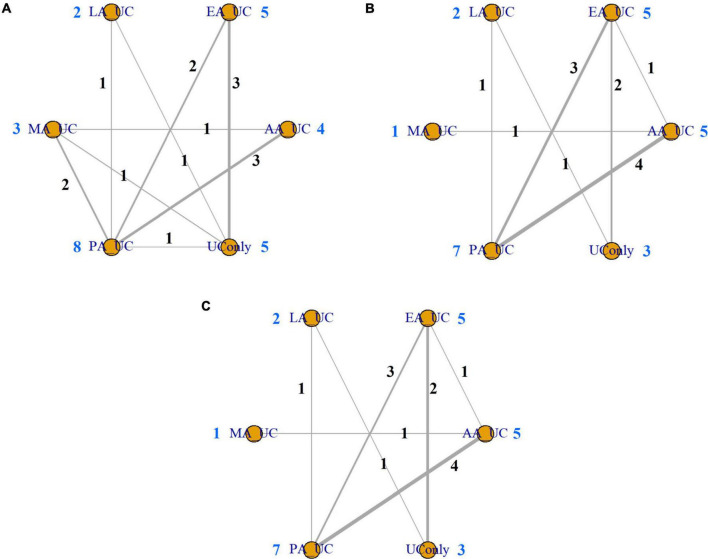
Network plot. **(A)** Body weight. **(B)** Body mass index. **(C)** Waist circumference. Network plots showing direct and indirect comparisons among various acupuncture modalities. The node size represents the number of studies included. AA, auricular acupuncture; BW, body weight; BMI, body mass index; EA, electroacupuncture; LA, laser acupuncture; MA, manual acupuncture; PA, placebo acupuncture; UC, usual care; WC, waist circumference.

In this study, we applied a random-effects model to the analysis under the assumption of homogeneity and verified the connectivity of the network by visualizing it as a network plot (Figure 2). To verify transitivity and consistency, we conducted a net-split test, including direct, indirect, and network estimations, and observed no studies showing heterogeneity. The results of the net-split test are shown in Supplementary Figure 3. Additionally, we used Markov chain MCMC diagnostics to estimate the effects of the Bayesian analyses, which are represented in Supplementary Figure 4. We performed Gelman-Rubin diagnostics, which are represented in Supplementary Figure 5.

#### 3.4.2 Comparative effectiveness of acupuncture modality on BW

**FIGURE 3:**
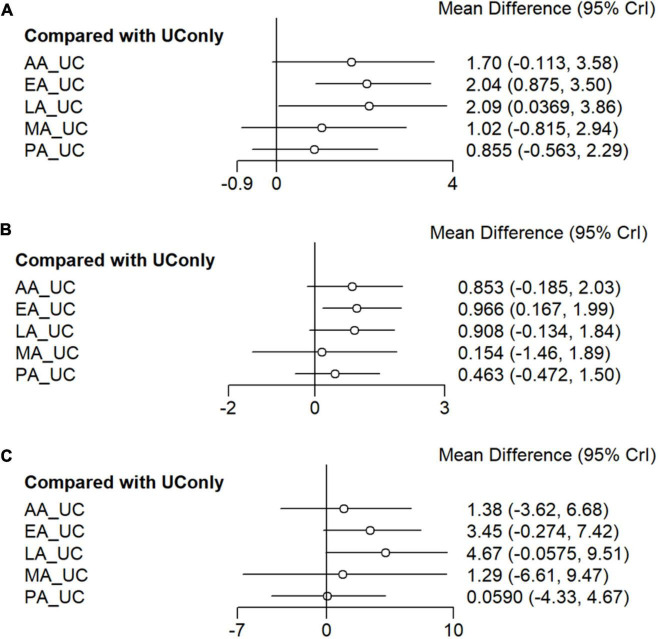
Network meta-analysis forest plot. **(A)** Body weight. **(B)** Body mass index. **(C)** Waist circumference. Network meta-analysis forest plot of primary and secondary outcomes. The forest plot was based on a random-effects model. This indicates the mean difference and 95% confidence interval for the effectiveness of the different acupuncture modalities versus usual care. The random-effects model was used to account for variability between studies. AA, auricular acupuncture; BW, body weight; BMI, body mass index; EA, electroacupuncture; LA, laser acupuncture; MA, manual acupuncture; PA, placebo acupuncture; UC, usual care; WC, waist circumference.

**FIGURE 4:**
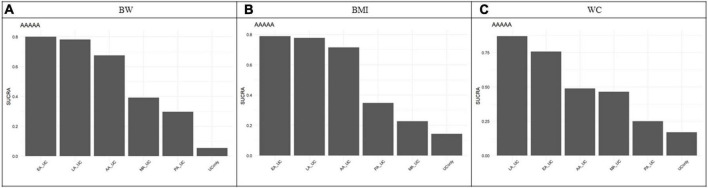
Surface Under the Cumulative Ranking Curve (SUCRA). **(A)** Body weight. **(B)** Body mass index. **(C)** Waist circumference. SUCRA showed the probability of each acupuncture modality being the best treatment for body weight, body mass index, and waist circumference. EA showed the highest SUCRA value for BW reduction (0.80), suggesting that it was the most effective modality. The SUCRA values vary from 0 to 1, where 1 indicates that the treatment might be the best and 0 indicates the worst. The probability of being the best treatment for reducing BW was highest for EA (0.8006), LA (0.7830), AA (0.6737), MA (0.3911), PA (0.2962), and UC alone (0.0553), BMI was highest for EA (0.8035), LA (0.7373), AA (0.7176), PA (0.3616), MA (0.2517), and UC alone (0.1284), WC was highest for LA (0.8685), EA (0.7577), AA (0.4891), MA (0.4648), PA (0.2501), and UC alone (0.1698). AA, auricular acupuncture; BW, body weight; BMI, body mass index; EA, electroacupuncture; LA, laser acupuncture; MA, manual acupuncture; PA, placebo acupuncture; SUCRA, surface under the cumulative ranking curve; UC, usual care; WC, waist circumference.

**FIGURE 5:**
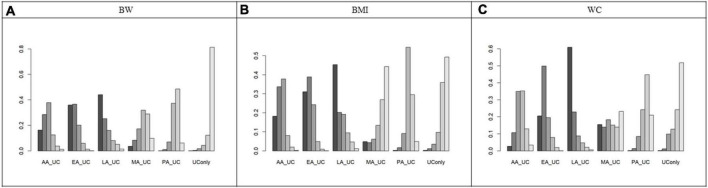
Rank plot. **(A)** Body weight **(B)** Body mass index **(C)** Waist circumference. Rank plot showing the probability of each acupuncture modality being the best treatment for body weight, body mass index, and waist circumference. AA, auricular acupuncture; BW, body weight; BMI, body mass index; EA, electroacupuncture; LA, laser acupuncture; MA, manual acupuncture; PA, placebo acupuncture; UC, usual care; WC, waist circumference.

An NMA of BW revealed the following effect sizes for UC alone versus each acupuncture modality in combination with UC: LA (MD = 2.09, 95% CI = 0.04 to 3.86), EA (MD = 2.04, 95% CI = 0.88 to 3.50), AA (MD = 1.69, 95% CI = −0.11 to 3.58), and MA (MD = 1.02, 95% CI = −0.82 to 2.94). Combined treatment with LA and EA significantly reduced BW compared with that in UC alone. The other interventions showed a tendency to reduce BW compared with that in UC only, but the difference was not significant (Figure 3A). Based on the SUCRA, the probability of being the best treatment for reducing BW was highest for EA (0.8006), followed by LA (0.7830), AA (0.6737), MA (0.3911), PA (0.2962), and UC alone (0.0553) (Figures 4A, 5A).

#### 3.4.3 Comparative effectiveness of acupuncture modality on BMI

An NMA of BMI revealed the following effect sizes for UC alone versus each acupuncture modality in combination with UC: EA (MD = 0.97, 95% CI = 0.17 to 2.00), LA (MD = 0.91, 95% CI = −0.13 to 1.84), AA (MD = 0.85, 95% CI = −0.19 to 2.03), and MA (MD = 0.15, 95% CI = −1.46 to 1.89). EA combined treatment significantly reduced BMI compared to that in UC alone. The other interventions showed a tendency to reduce BMI compared to that in UC only, although the difference was not significant (Figure 3B). Based on the SUCRA, the probability of being the best treatment for reducing BMI was highest for EA (0.8035), LA (0.7373), AA (0.7176), PA (0.3616), MA (0.2517), and UC alone (0.1284) (Figures 4B, 5B).

#### 3.4.4 Comparative effectiveness of acupuncture modality on WC

An NMA of WC revealed the following effect sizes for UC alone versus each acupuncture modality in combination with UC: LA (MD = 4.67, 95% CI = −0.06 to 9.51), EA (MD = 3.45, 95% CI = −0.27 to 7.42), AA (MD = 1.38, 95% CI = −3.62 to 6.68), and MA (MD = 1.29, 95% CI = −6.61 to 7.47). Combined treatment with LA and EA significantly reduced WC compared to that in treatment with UC alone. The other interventions showed a tendency to reduce WC compared to that in UC only but were not significant, as the 95% CI included zero (Figure 3C). Based on the SUCRA, the probability of being the best treatment for reducing WC was highest for LA (0.8685), followed by EA (0.7577), AA (0.4891), MA (0.4648), PA (0.2501), and UC alone (0.1698) (Figures 4C, 5C).

### 3.5 Safety

The analysis showed that the main reported adverse events in the experimental group were 9 cases of mild ecchymosis, 7 cases of mild tenderness, and 4 cases of abdominal discomfort. In the control group, the main adverse events were 2 cases of mild tenderness, 1 case of abdominal discomfort, and 1 case of dizziness. The PMA of AEs demonstrated no significant differences between UC alone and each acupuncture modality in combination with UC. However, the incidence of adverse events was observed to be significantly higher in the combination of each acupuncture modality with the UC group than that in the PA+UC group (RR = 4.05, 95% CI = 1.61 to 10.20) (Supplementary Figure 6). The drop-out rate was not significant (Supplementary Figure 7).

### 3.6 Publication bias

To assess the possibility of bias in the NMA of the main outcome, BW, and the secondary outcomes, BMI and WC, a funnel plot was used for visualization. The Egger test and visual inspection suggested the possibility of publication bias. Therefore, this study should be interpreted with caution because of potential publication bias. A funnel plot is shown in Supplementary Figure 8.

## 4 Discussion

### 4.1 Summary of findings

We conducted a systematic review and network meta-analysis to compare various acupuncture modalities for patients with obesity and overweight. Fourteen RCTs with 868 participants were selected for the analysis. The PMA results demonstrated that the combination of EA and UC significantly reduced both BW and BMI compared to those in UC alone. NMA revealed that the combination of EA and UC significantly reduced BW, BMI, and WC compared to those in UC alone. Additionally, the combination of LA and UC significantly reduced BW and WC. The probability of each modality being the optimal treatment was evaluated using SUCRA; EA was identified as the most efficacious treatment for BW and BMI, whereas LA was the most efficacious treatment for WC. Acupuncture treatment did not increase the incidence of side effects or serious adverse events.

### 4.2 Debate

#### 4.2.1 Comparison with existing literature

Kim et al. (26) conducted the first systematic review with a separate meta-analysis of the effects of distinct modalities on obesity, comparing the effects of MA, pharmacopuncture, acupoint catgut embedding, LA, AA, and EA with those of PA. To address the issue of a limited sample size, Kim’s study described the effect estimates of outcomes with different units as standardized mean differences. In contrast, the current study described the effect estimates of BW, BMI, and WC as mean differences to provide an intuitive reference for clinicians in decision-making. Zhang et al. (27) was an NMA study that compared the effectiveness of different treatments for obesity, including AA, stimulation, acupuncture, pharmacotherapy, and relative therapy. The study was limited to simple obesity, included acupressure as an intervention, and permitted combination therapy between the interventions to establish the treatment groups. By contrast, this study is more representative of the obese population encountered in clinical practice, including patients with a wide range of complications. Furthermore, the study evaluated the efficacy of a single acupuncture treatment to inform clinical decision-making and thus excluded treatments that were a combination of acupressure or two or more Traditional East Asian Medicine (TEAM) interventions.

#### 4.2.2 Effects and mechanisms of laser acupuncture and electroacupuncture

In addition to reducing BW, BMI, and WC, acupuncture regulates the endocrine system, promotes digestion, and attenuates oxidative stress and obesity-related peptides and inflammatory markers (57). Laboratory evidence indicates that acupuncture regulates lipid metabolism, modulates inflammatory responses, and promotes the browning of white adipose tissue. Furthermore, acupuncture has been demonstrated to suppress appetite by regulating appetite regulatory hormones and downstream signaling pathway (58). EA exerts antinociceptive and anti-inflammatory effects by regulating the balance of neural-immune–endocrine interactions. EA modulates immune cells, inflammatory cytokines, and peripheral nociceptors at the peripheral level and involves spinal nociceptive neurons and glial cells at the central level (59). EA and MA have been demonstrated to reduce abnormal 5-hydroxytryptamine and corticotropin-releasing hormone concentrations in patients with irritable bowel syndrome. Additionally, EA and MA have been shown to improve intestinal motility and sensitivity by regulating the peptides of the brain-gut axis, such as substance P, vasoactive intestinal peptide, and neuropeptide Y, in the large intestine (60). Consequently, the mechanisms of acupuncture treatment for obesity have been elucidated in considerable detail, which is beneficial for selecting appropriate acupuncture modalities according to the target symptoms of obese patients in clinical practice.

#### 4.2.3 Effectiveness of acupuncture for obesity-related conditions

Acupuncture is used to treat a variety of obesity-related conditions, and its effectiveness has been previously investigated. Recent studies have suggested that major depressive disorder (MDD) and obesity are interrelated in areas such as genetics, immunology, and endocrinology (61). Zhichao et al. (62) compared the effects of several acupuncture treatments and Western medications [selective serotonin reuptake inhibitors (SSRIs), noradrenergic, and specific serotonergic antidepressants] on MDD with NMA. The findings indicated that EA, MA, and Western medications significantly reduced MDD. The combination of EA and SSRIs demonstrated the greatest effect. Polycystic ovary syndrome (PCOS) is closely associated with body weight and BMI, with obesity representing a prominent phenotype in women with PCOS (63). Pan et al. (64) conducted RCTs comparing MA and PA combined with personalized herbal medicine in women with PCOS. The results demonstrated that the MA group exhibited a significantly greater reduction in PCOS scores than those in the PA group.

### 4.3 Implications for further study and clinical practice

The results of this study demonstrated that EA and LA were the most effective treatments for obesity, with no significant difference in safety compared with that in UC alone, which is consistent with previous studies (65). However, while EA is widely used in clinical practice, LA is not. Based on the results of the current study, it is possible to consider the active use of LA for the treatment of obesity in clinical practice. The acupuncture treatment methods employed in the studies included in the systematic review differed, and there may have been variations in their effectiveness. However, this aspect was not examined in the current study. Future studies should compare the effectiveness of slightly modified acupuncture methods in different clinical settings to determine whether they influence the outcomes of obesity treatment. Furthermore, since the effectiveness of obesity treatment may vary depending on the initial weight of the study participants, it is necessary to apply methodologies such as meta-regression analysis to examine the effectiveness of acupuncture treatment according to obesity level. In this study, only acupuncture was used for isolation. Future studies should investigate the potential synergistic effects of combining two or more TEAM treatments. Furthermore, although this study focused on BW, BMI, and WC outcomes, other indicators of obesity, such as inflammation, blood pressure, and blood glucose levels, should also be studied.

### 4.4 Strengths and limitations

This study included patients with simple and complex obesity; therefore, the results are generalizable because the patient population is similar to the obese patients encountered in clinical practice. In addition, this approach has the advantage of not combining different clinical outcomes and expressing MD results for each clinical outcome. Nevertheless, certain limitations should be noted. Although an extensive search was conducted using five English-language databases, the number of included studies was limited, and publication bias may have occurred, necessitating a cautious interpretation of the results. Another limitation is that despite some dropouts occurring, most included studies did not clearly state whether they used intention-to-treat (ITT) or per-protocol (PP) in their statistical analysis. This could introduce bias and affect the generalizability of the results. Future studies should clearly define the statistical analysis methods, specifying ITT as the primary outcome analysis and PP for sensitivity analysis, and present the results accordingly to enhance the reliability of the findings. Additionally, the overall number of reported adverse events in the included studies was relatively small, and some studies did not report on adverse events. Therefore, further observational studies are needed to track the incidence of adverse events in acupuncture treatment for obesity. Furthermore, the number of acupuncture sessions and treatment duration in the included studies were insufficient to examine the long-term effects of acupuncture. Most studies lasted between six and 12 weeks. Future clinical studies on acupuncture for obesity should include longer follow-up periods to better understand the long-term effects and ensure interpretive robustness of the results.

## 5 Conclusion

A systematic review and network meta-analysis were conducted to compare various acupuncture modalities for the treatment of patients with obesity and overweight. The analysis was conducted on 14 RCTs that included 868 participants. The PMA findings demonstrated that the combination of EA and UC was associated with a significantly greater reduction in both BW and BMI than that in UC alone. The probability of each modality being the optimal treatment was evaluated using SUCRA; EA was observed to be the most efficacious treatment for BW and BMI, whereas LA was the most efficacious treatment for WC. Based on the results of this study, it is possible to consider the active use of EA and LA for the treatment of obesity in clinical practice.

## Data Availability

The original contributions presented in this study are included in this article/Supplementary material, further inquiries can be directed to the corresponding authors.

## Footnotes

1
https://cran.r-project.org/web/packages/gemtc/index.html

2
https://github.com/cran/netmeta.git

## References

[1] World Health Organization. *Obesity and overweight.* Geneva: World Health Organization (2022).

[2] World Health Organization. *WHO Consultation on obesity: Preventing and managing the global epidemic: Report of a WHO consultation.* Geneva: World Health Organization (2000). p. 252. 11234459

[3] Flegal K Carroll M Ogden C Johnson C. Prevalence and trends in obesity among US adults, 1999-2000. *JAMA.* (2002) 288:1723–7. 10.1001/jama.288.14.1723 12365955

[4] Bomberg E Birch L Endenburg N German A Neilson J Seligman H The financial costs, behaviour and psychology of obesity: A one health analysis. *J Comp Pathol.* (2017) 156:310–25. 10.1016/j.jcpa.2017.03.007 28460796

[5] Apovian C. Obesity: Definition, comorbidities, causes, and burden. *Am J Manag Care.* (2016) 22:s176–85. 27356115

[6] Dobbs R Sawers C Thompson F Manyika J Woetzel J Child P *Overcoming obesity: An initial economic analysis.* New York, NY: McKinsey global institute (2014).

[7] Conway B Rene A. Obesity as a disease: No lightweight matter. *Obes Rev.* (2004) 5:145–51. 10.1111/j.1467-789X.2004.00144.x 15245383

[8] Tesauro M Cardillo C. Obesity, blood vessels and metabolic syndrome. *Acta Physiol.* (2011) 203:279–86. 10.1111/j.1748-1716.2011.02290.x 21439028

[9] Engin A. The definition and prevalence of obesity and metabolic syndrome. In: Engin A Engin A editors. *Obesity and lipotoxicity. Advances in experimental medicine and biology.* Cham: Springer International Publishing (2017). p. 1–17. 10.1007/978-3-319-48382-5_1 28585193

[10] Després J Lemieux I. Abdominal obesity and metabolic syndrome. *Nature.* (2006) 444:881–7. 10.1038/nature05488 17167477

[11] Kopelman P. Health risks associated with overweight and obesity. *Obes Rev.* (2007) 8:13–7. 10.1111/j.1467-789X.2007.00311.x 17316295

[12] Cawley J Meyerhoefer C. The medical care costs of obesity: An instrumental variables approach. *J Health Econ.* (2012) 31:219–30. 10.1016/j.jhealeco.2011.10.003 22094013

[13] Cawley J Biener A Meyerhoefer C Ding Y Zvenyach T Smolarz B Direct medical costs of obesity in the United States and the most populous states. *J Manag Care Spec Pharm.* (2021) 27:354–66. 10.18553/jmcp.2021.20410 33470881 PMC10394178

[14] Kushner R Foster G. Obesity and quality of life. *Nutrition.* (2000) 16:947–52. 10.1016/s0899-9007(00)00404-4 11054600

[15] de Andrade Mesquita L Fagundes Piccoli G Richter da Natividade G Frison Spiazzi B Colpani V Gerchman F. Is lorcaserin really associated with increased risk of cancer? A systematic review and meta-analysis. *Obes Rev.* (2021) 22:e13170. 10.1111/obr.13170 33258543

[16] Nissen S Wolski K Prcela L Wadden T Buse J Bakris G Effect of naltrexone-bupropion on major adverse cardiovascular events in overweight and obese patients with cardiovascular risk factors: A randomized clinical trial. *JAMA.* (2016) 315:990–1004. 10.1001/jama.2016.1558 26954408

[17] Garvey W Mechanick J Brett E Garber A Hurley D Jastreboff A AMERICAN ASSOCIATION OF CLINICAL ENDOCRINOLOGISTS AND AMERICAN COLLEGE OF ENDOCRINOLOGY COMPREHENSIVE CLINICAL PRACTICE GUIDELINES FOR MEDICAL CARE OF PATIENTS WITH OBESITY. *Endocr Pract.* (2016) 22:1–203. 10.4158/EP161365.GL 27219496

[18] Aronne L Sattar N Horn D Bays H Wharton S Lin W Continued Treatment with tirzepatide for maintenance of weight reduction in adults with obesity: The SURMOUNT-4 randomized clinical trial. *JAMA.* (2024) 331:38–48. 10.1001/jama.2023.24945 38078870 PMC10714284

[19] Marihart C Brunt A Geraci A. Older adults fighting obesity with bariatric surgery: Benefits, side effects, and outcomes. *SAGE Open Med.* (2014) 2:2050312114530917. 10.1177/2050312114530917 26770722 PMC4607185

[20] DeMaria E Murr M Byrne T Blackstone R Grant J Budak A Validation of the obesity surgery mortality risk score in a multicenter study proves it stratifies mortality risk in patients undergoing gastric bypass for morbid obesity. *Ann Surg.* (2007) 246:578–582; discussion 583–584. 10.1097/SLA.0b013e318157206e 17893494

[21] Jammah A. Endocrine and metabolic complications after bariatric surgery. *Saudi J Gastroenterol.* (2015) 21:269–77. 10.4103/1319-3767.164183 26458852 PMC4632250

[22] Davis M West A Weeks W Sirovich B. Health behaviors and utilization among users of complementary and alternative medicine for treatment versus health promotion. *Health Serv Res.* (2011) 46:1402–16. 10.1111/j.1475-6773.2011.01270.x 21554272 PMC3207184

[23] Lee E Yoon S Kim H Kim Y Leem J Park J. Ephedrae Herba in combination with herbal medicine (Zhizichi decoction and Phellodendri Cortex) for weight reduction: A case series. *Integr Med Res.* (2020) 9:100408. 10.1016/j.imr.2020.100408 32405455 PMC7210583

[24] Sharpe P Blanck H Williams J Ainsworth B Conway J. Use of complementary and alternative medicine for weight control in the United States. *J Altern Complement Med.* (2007) 13:217–22. 10.1089/acm.2006.6129 17388764

[25] Gao Y Wang Y Zhou J Hu Z Shi Y. Effectiveness of electroacupuncture for simple obesity: A systematic review and meta-analysis of randomized controlled trials. *Evid Based Complement Alternat Med.* (2020) 2020:2367610. 10.1155/2020/2367610 32714399 PMC7341404

[26] Kim S Shin I Park Y. Effect of acupuncture and intervention types on weight loss: A systematic review and meta-analysis. *Obes Rev.* (2018) 19:1585–96. 10.1111/obr.12747 30180304

[27] Zhang Y Li J Mo G Liu J Yang H Chen X Acupuncture and related therapies for obesity: A network meta-analysis. *Evid Based Complement Alternat Med.* (2018) 2018:9569685. 10.1155/2018/9569685 30363899 PMC6186334

[28] Rouse B Chaimani A Li T. Network meta-analysis: An introduction for clinicians. *Intern Emerg Med.* (2017) 12:103–11. 10.1007/s11739-016-1583-7 27913917 PMC5247317

[29] Hutton B Salanti G Caldwell D Chaimani A Schmid C Cameron C The PRISMA extension statement for reporting of systematic reviews incorporating network meta-analyses of health care interventions: Checklist and explanations. *Ann Intern Med.* (2015) 162:777–84. 10.7326/M14-2385 26030634

[30] OECD. *The heavy burden of obesity: The economics of prevention.* Paris: Organisation for Economic Co-operation and Development (2019).

[31] Reik A Holzapfel C. Randomized controlled lifestyle intervention (LION) study for weight loss and maintenance in adults with obesity-design and methods. *Front Nutr.* (2020) 7:586985. 10.3389/fnut.2020.586985 33240920 PMC7683381

[32] Delpino F Figueiredo L. Melatonin supplementation and anthropometric indicators of obesity: A systematic review and meta-analysis. *Nutrition.* (2021) 9:111399. 10.1016/j.nut.2021.111399 34626955

[33] Cochrane Training. *Cochrane handbook for systematic reviews of interventions.* (2022). Available online at: https://training.cochrane.org/handbook (accessed November 18, 2022).

[34] Shim S Kim S. Intervention meta-analysis: Application and practice using R software. *Epidemiol Health.* (2019) 41:e2019008. 10.4178/epih.e2019008 30999738 PMC6545497

[35] Higgins J Thompson S Deeks J Altman D. Measuring inconsistency in meta-analyses. *BMJ.* (2003) 327:557–60. 10.1136/bmj.327.7414.557 12958120 PMC192859

[36] Watt J Tricco A Straus S Veroniki A Naglie G Drucker A. Research techniques made simple: Network meta-analysis. *J Invest Dermatol.* (2019) 139:4–12.e1. 10.1016/j.jid.2018.10.028 30579427

[37] van Valkenhoef G. *GeMTC R package.* (2022). Available online at: https://github.com/gertvv/gemtc (accessed November 18, 2022).

[38] Ades A Sculpher M Sutton A Abrams K Cooper N Welton N Bayesian methods for evidence synthesis in cost-effectiveness analysis. *Pharmacoeconomics.* (2006) 24:1–19. 10.2165/00019053-200624010-00001 16445299

[39] van Valkenhoef G Lu G de Brock B Hillege H Ades A Welton N. Automating network meta-analysis. *Res Synth Methods.* (2012) 3:285–99. 10.1002/jrsm.1054 26053422

[40] Salanti G Ades A Ioannidis J. Graphical methods and numerical summaries for presenting results from multiple-treatment meta-analysis: An overview and tutorial. *J Clin Epidemiol.* (2011) 64:163–71. 10.1016/j.jclinepi.2010.03.016 20688472

[41] Sterne J Egger M. Funnel plots for detecting bias in meta-analysis: Guidelines on choice of axis. *J Clin Epidemiol.* (2001) 54:1046–55. 10.1016/s0895-4356(01)00377-8 11576817

[42] Egger M Davey Smith G Schneider M Minder C. Bias in meta-analysis detected by a simple, graphical test. *BMJ.* (1997) 315:629–34. 10.1136/bmj.315.7109.629 9310563 PMC2127453

[43] Hsu C Hwang K Chao C Chang H Chou P. Electroacupuncture in obese women: A randomized, controlled pilot study. *J Womens Health (Larchmt).* (2005) 14:434–40. 10.1089/jwh.2005.14.434 15989416

[44] Hsu C Wang C Hwang K Lee T Chou P Chang H. The effect of auricular acupuncture in obese women: A randomized controlled trial. *J Womens Health (Larchmt).* (2009) 18:813–8. 10.1089/jwh.2008.1005 19445642

[45] Yasemin C Turan S Kosan Z. The effects of auricular and body acupuncture in Turkish obese female patients: A randomized controlled trial indicated both methods lost body weight but auricular acupuncture was better than body acupuncture. *Acupunct Electrother Res.* (2017) 42:1–10. 10.3727/036012917x14908026364990 29772131

[46] Fogarty S Stojanovska L Harris D Zaslawski C Mathai M McAinch AJ. A randomised cross-over pilot study investigating the use of acupuncture to promote weight loss and mental health in overweight and obese individuals participating in a weight loss program. *Eat Weight Disord.* (2015) 20:379–87. 10.1007/s40519-014-0175-7 25630840

[47] Yang J Xing H Xiao H Li Q Li M Wang S. [Effects of acupuncture combined with diet adjustment and aerobic exercise on weight and waist-hip ratio in simple obesity patients]. *Zhongguo Zhen Jiu.* (2010) 30:555–8. 20862937

[48] Tseng C Tseng A Tseng J Chang C. Effect of laser acupuncture on anthropometric measurements and appetite sensations in obese subjects. *Evid Based Complement Alternat Med.* (2016) 2016:9365326. 10.1155/2016/9365326 27051454 PMC4804083

[49] Lien C Liao L Chou P Hsu C. Effects of auricular stimulation on obese women: A randomized, controlled clinical trial. *Eur J Integr Med.* (2012) 4:e45–53. 10.1016/j.eujim.2011.12.002

[50] Darbandi M Darbandi S Owji A Mokarram P Mobarhan M Fardaei M Auricular or body acupuncture: Which one is more effective in reducing abdominal fat mass in Iranian men with obesity: A randomized clinical trial. *J Diabetes Metab Disord.* (2014) 13:92. 10.1186/s40200-014-0092-3 25505744 PMC4261582

[51] Ni W Wang P Chen H Liu Y Zhang W Qiu L Obesity complicated with insulin resistance treated with the electroacupuncture at the combination of back-shu and front-mu points. *World J Acupunct Moxibust.* (2022) 32:213–7. 10.1016/j.wjam.2021.12.004

[52] Chung J Kim J Lee S Sung-Keel K. Effects of electroacupuncture on parameters related to obesity in adults with abdominal obesity: Three arm randomized single blind pilot study. *J Acupunct Res.* (2010) 27:43–57.

[53] Kim S Jang E Na W Lee S Lee J Moon H A pilot study of Sa-am acupuncture treatment used by sham acupuncture for the simple obesity. *J Acupunct Res.* (2007) 24:67–88.

[54] Abdi H Abbasi-Parizad P Zhao B Ghayour-Mobarhan M Tavallaie S Rahsepar A Effects of auricular acupuncture on anthropometric, lipid profile, inflammatory, and immunologic markers: A randomized controlled trial study. *J Altern Complement Med.* (2012) 18:668–77. 10.1089/acm.2011.0244 22788576

[55] Abdi H Zhao B Darbandi M Ghayour-Mobarhan M Tavallaie S Rahsepar A The effects of body acupuncture on obesity: Anthropometric parameters, lipid profile, and inflammatory and immunologic markers. *ScientificWorldJournal.* (2012) 2012:603539. 10.1100/2012/603539 22649299 PMC3353309

[56] El-Mekawy H ElDeeb A Ghareib H. Effect of laser acupuncture combined with a diet-exercise intervention on metabolic syndrome in post-menopausal women. *J Adv Res.* (2015) 6:757–63. 10.1016/j.jare.2014.08.002 26425364 PMC4563594

[57] Belivani M Dimitroula C Katsiki N Apostolopoulou M Cummings M Hatzitolios A. Acupuncture in the treatment of obesity: A narrative review of the literature. *Acupunct Med.* (2013) 31:88–97. 10.1136/acupmed-2012-010247 23153472

[58] Wang L Huang W Wei D Ding D Liu Y Wang J Mechanisms of acupuncture therapy for simple obesity: An evidence-based review of clinical and animal studies on simple obesity. *Evid Based Complement Alternat Med.* (2019) 2019:5796381. 10.1155/2019/5796381 30854010 PMC6378065

[59] Jun M Kim Y Kim J. Modern acupuncture-like stimulation methods: A literature review. *Integr Med Res.* (2015) 4:195–219. 10.1016/j.imr.2015.09.005 28664127 PMC5481834

[60] Ma X Hong J An C Zhang D Huang Y Wu H Acupuncture-moxibustion in treating irritable bowel syndrome: How does it work? *World J Gastroenterol.* (2014) 20:6044–54. 10.3748/wjg.v20.i20.6044 24876727 PMC4033444

[61] Milaneschi Y Simmons W van Rossum E Penninx B. Depression and obesity: Evidence of shared biological mechanisms. *Mol Psychiatry.* (2019) 24:18–33. 10.1038/s41380-018-0017-5 29453413

[62] Zhichao H Ching L Huijuan L Liang Y Zhiyu W Weiyang H A network meta-analysis on the effectiveness and safety of acupuncture in treating patients with major depressive disorder. *Sci Rep.* (2021) 11:10384. 10.1038/s41598-021-88263-y 34001924 PMC8129113

[63] Teede H Joham A Paul E Moran L Loxton D Jolley D Longitudinal weight gain in women identified with polycystic ovary syndrome: Results of an observational study in young women. *Obesity.* (2013) 21:1526–32. 10.1002/oby.20213 23818329

[64] Pan W Li F Wang Q Huang Z Yan Y Zhao L A randomized sham-controlled trial of manual acupuncture for infertile women with polycystic ovary syndrome. *Integr Med Res.* (2022) 11:100830. 10.1016/j.imr.2021.100830 35059289 PMC8760432

[65] Yang J Mallory M Wu Q Bublitz S Do A Xiong D The safety of laser acupuncture: A systematic review. *Med Acupunct.* (2020) 32:209–17. 10.1089/acu.2020.1419 32874405 PMC7455477

