# Supplementary material for: Effectiveness and safety of acupuncture modalities for overweight and obesity treatment: a systematic review and network meta-analysis of RCTs
Source: Front Med (Lausanne). 2024 Aug 21;11:1446515. doi: 10.3389/fmed.2024.1446515 (PMC11372581; doi:10.3389/fmed.2024.1446515)
Supplement: Supplementary file 1 [file Data_Sheet_1.docx]

Supplementary Material

**Supplementary Figure Legends**

**Supplementary Figures 1.** Risk of Bias – Traffic plot

Traffic plots show the risk of bias assessment for each included study using the Cochrane risk-of-bias tool. Each domain is colored to represent a low, unclear, or high risk of bias.

**Supplementary Figures 2.** Pairwise meta-analysis forest plot

Supplementary Figure 2-(a) Acupuncture + usual care vs. usual care (body weight)

Supplementary Figure 2-(b) Placebo Acupuncture + usual care vs. usual care alone (body weight)

Supplementary Figure 2-(c) Acupuncture + usual care vs. usual care alone (body mass index)

Supplementary Figure 2-(d) Placebo Acupuncture + usual care vs. usual care alone (body mass index)

Supplementary Figure 2-(e) Acupuncture + usual care vs. usual care (waist circumference)

Supplementary Figure 2-(f) Placebo Acupuncture + usual care vs. usual care (waist circumference)

Pairwise meta-analysis forest plots for primary and secondary outcomes. The forest plot was based on a random-effects model. This indicates the mean difference and 95% confidence interval for the effectiveness of the different acupuncture modalities versus usual care. The random-effects model was used to account for variability between studies.

**Supplementary Figure 3.** Inconsistency test – Netsplit test

Supplementary Figure 3-(a) Body weight

Supplementary Figure 3-(b) Body mass index

Supplementary Figure 3-(c) Waist circumference

Results of the network split test for transitivity and consistency, including direct, indirect, and network estimations. No significant inconsistency was observed between the direct and indirect comparisons of BW (P >0.05), supporting the reliability of the network meta-analysis model.

**Supplementary Figures 4.** Markov Chain Monte Carlo (MCMC) diagnostics

Supplementary Figures 4-(a) Body weight

Supplementary Figures 4-(b) Body mass index

Supplementary Figures 4-(c) Waist circumference

MCMC diagnostics for Bayesian analysis showing trace plots and density plots for (a) body weight, (b) body mass index, and (c) waist circumference. Convergence is indicated by stable trace plots and PSRF values close to 1.

**Supplementary Figures 5.** Gelman-rubin diagnostics

Supplementary Figures 5-(a) Body weight

Supplementary Figures 5-(b) Body mass index

Supplementary Figures 5-(c) Waist circumference

Gelman-Rubin diagnostics assessing model convergence for (a) body weight, (b) body mass index, and (c) waist circumference. The potential scale reduction factor values for all the parameters were close to 1, indicating satisfactory convergence. AA: Auricular acupuncture; BMI: Body mass index; BW: Body weight; EA: Electroacupuncture; LA: Laser acupuncture; MA: Manual acupuncture; PA: Placebo acupuncture; UC: Usual care; WC: Waist circumference

**Supplementary Figure 6.** Adverse effect rate – forest plot

Supplementary Figure 6-(a) acupuncture + usual care vs. usual care only

Supplementary Figure 6-(b) acupuncture + usual care vs. placebo acupuncture + usual care

Forest plots showing the risk ratios and 95% confidence intervals for the adverse effect rate in (a) acupuncture + usual care vs. usual care only, and (b) acupuncture + usual care vs. placebo acupuncture + usual care. No significant increases in adverse effects were observed for any of these modalities.

**Supplementary Figure 7.** Drop-out rate – forest plot

Supplementary Figure 7-(a) acupuncture + usual care vs. usual care only

Supplementary Figure 7-(b) acupuncture + usual care vs. placebo acupuncture + usual care

Forest plots showing the risk ratios and 95% confidence intervals for the dropout rate in (a) acupuncture + usual care vs. usual care only and (b) acupuncture + usual care vs. placebo acupuncture + usual care. The dropout rates were similar across all modalities, with no significant differences.

**Supplementary Figure 8.** Funnel plot

Supplementary Figures 8-(a) Body weight

Supplementary Figures 8-(b) Body mass index

Supplementary Figures 8-(c) Waist circumference

Funnel plots were used to assess the publication bias for (a) body weight, (b) body mass index, and (c) waist circumference. The symmetry in the plots suggests a low publication bias. AA: Auricular acupuncture; BMI: Body mass index; BW: Body weight; EA: Electroacupuncture; LA: Laser acupuncture; MA: Manual acupuncture; PA: Placebo acupuncture; UC: Usual care; WC: Waist circumference

**Supplementary Table Legends**

**Supplementary Table 1.** Detailed Search Strategy

The detailed search strategy employed in the systematic review included the databases searched (PubMed, Central, Embase via Elsevier, CINAHL, and AMED), search terms used, and the date of the last search (May 8, 2024).

**Supplementary Table 2.** Excluded Reports by Eligibility Assessment

List of reports excluded during the eligibility assessment stage, including reasons for exclusion (e.g., inappropriate population, intervention, control group, or outcomes).

**Supplementary Figures 1.** Risk of Bias – Traffic plot

**
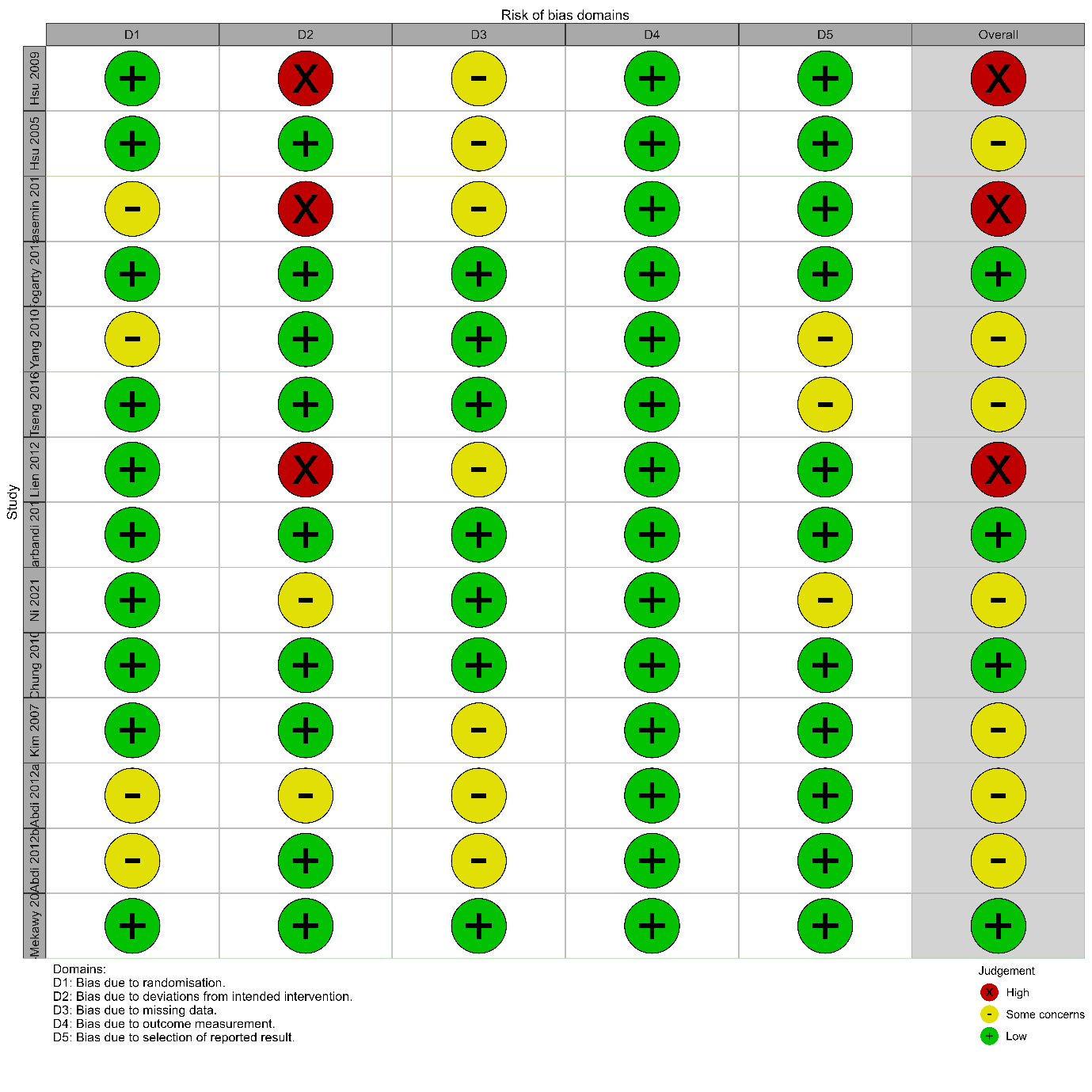
**

**Supplementary Figures 2.** Pairwise meta-analysis forest plot

**Supplementary Figure 2-(a)** Acupuncture + usual care vs usual care (Body weight)


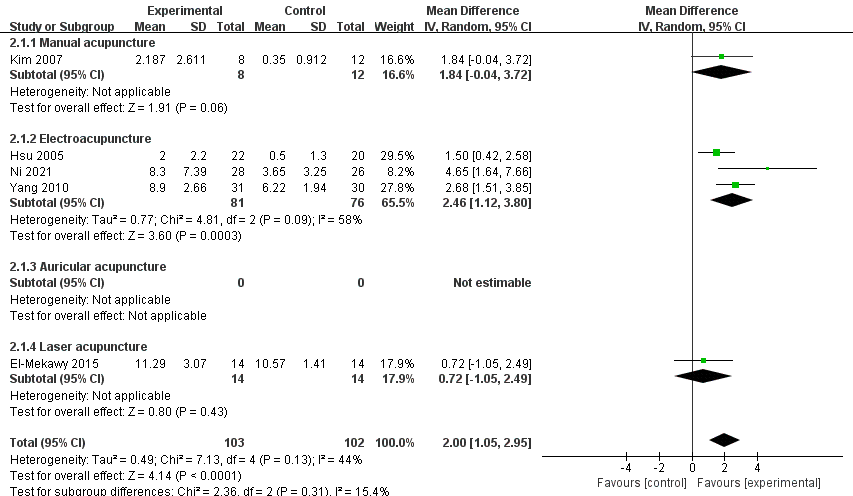


**Supplementary Figure 2-(b)** Placebo Acupuncture + usual care vs usual care (Body weight)

**
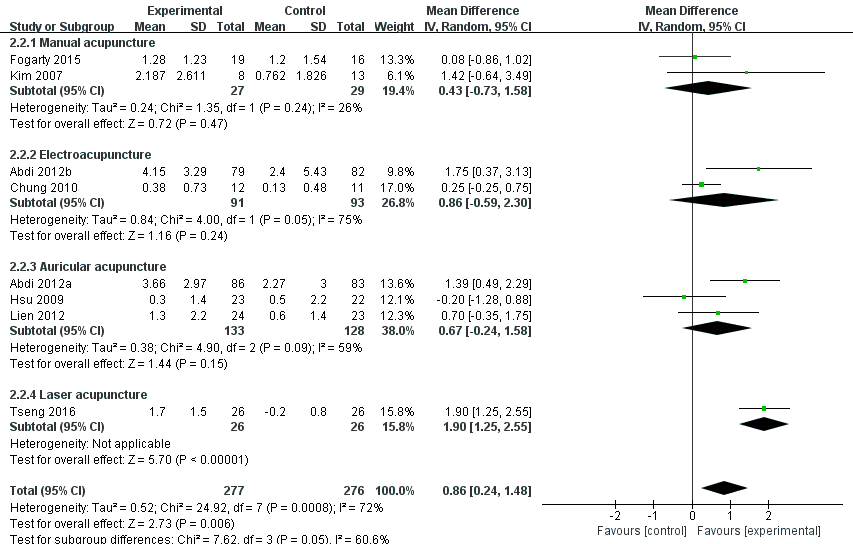
**

**Supplementary Figure 2-(c)** Acupuncture + usual care vs usual care (Body mass index)

**
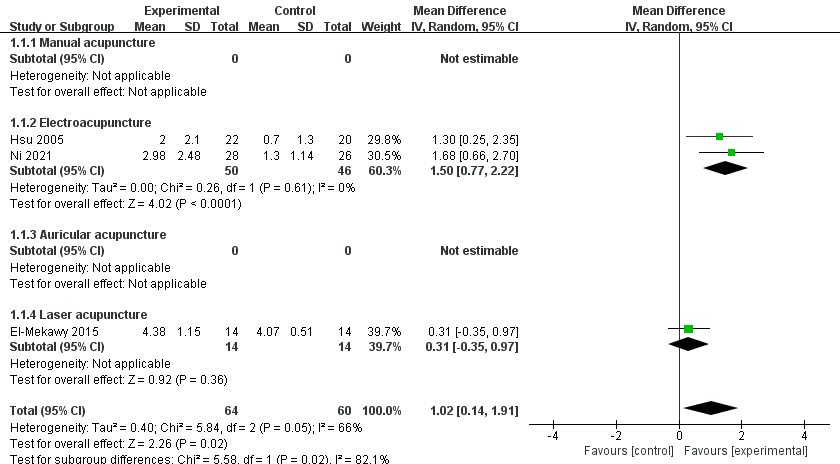
**

**Supplementary Figure 2-(d)** Placebo Acupuncture + usual care vs usual care (Body mass index)

**
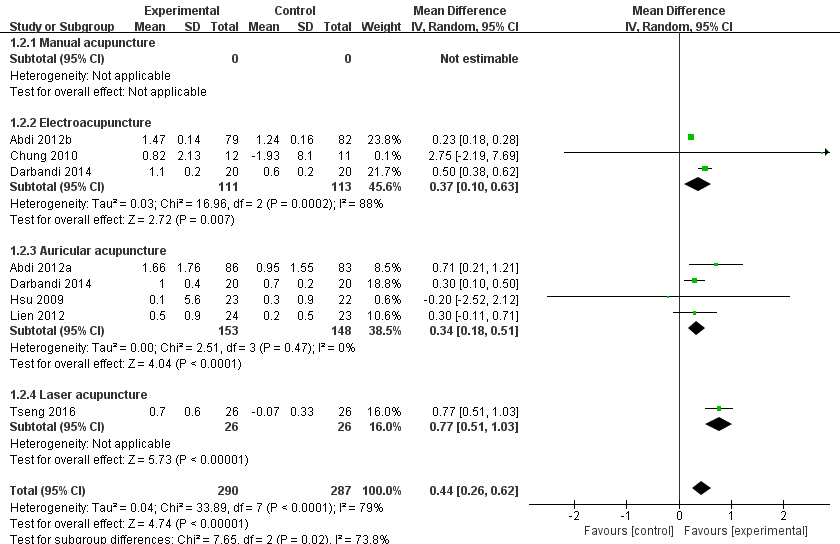
**

**Supplementary Figure 2-(e)** Acupuncture + usual care vs usual care (Waist circumference)

**
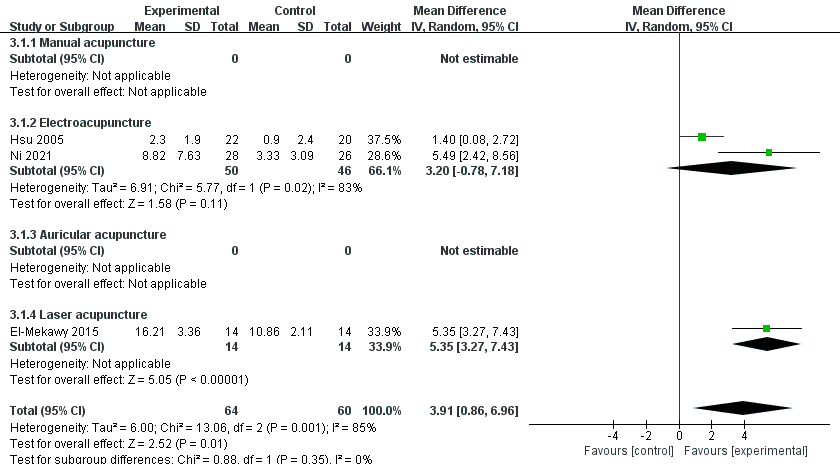
**

**Supplementary Figure 2-(f)** Placebo Acupuncture + usual care vs usual care (Waist circumference)

**
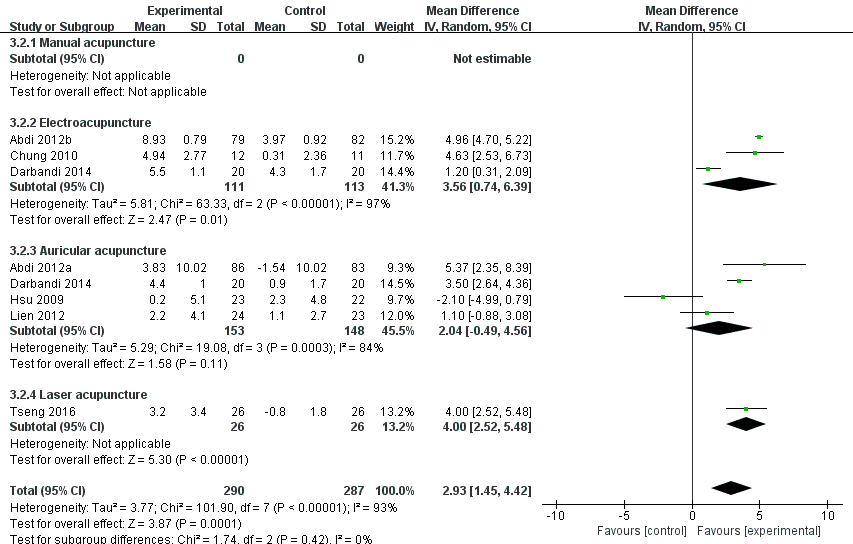
**

**Supplementary Figure 3.** Inconsistency test – Netsplit test

**Supplementary Figure 3-(a)** Body weight

**
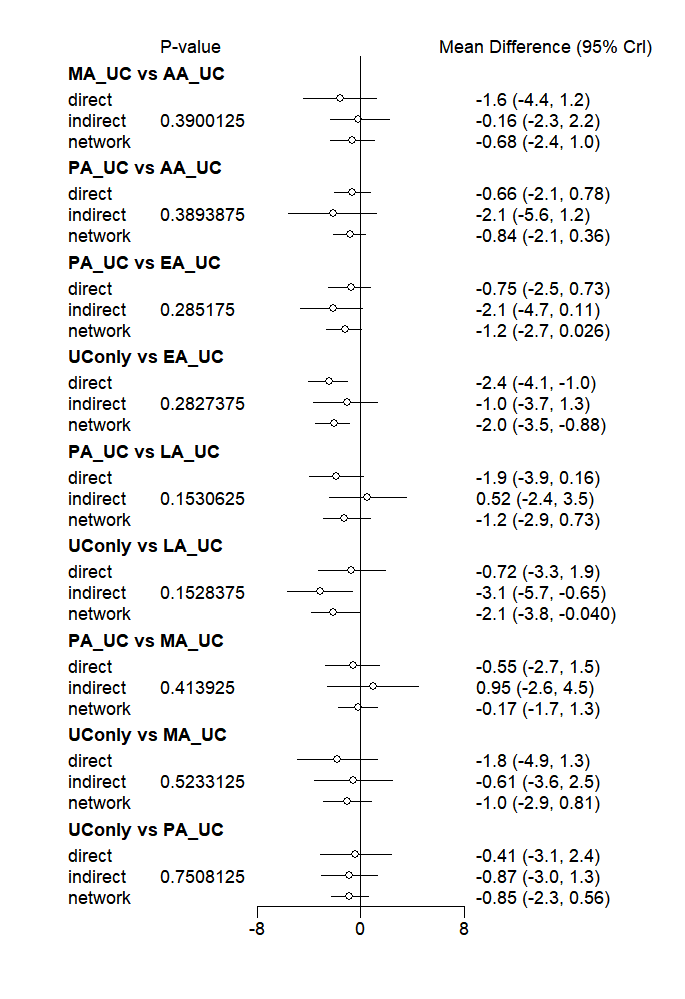
**

**Supplementary Figure 3-(b)** Body mass index

**
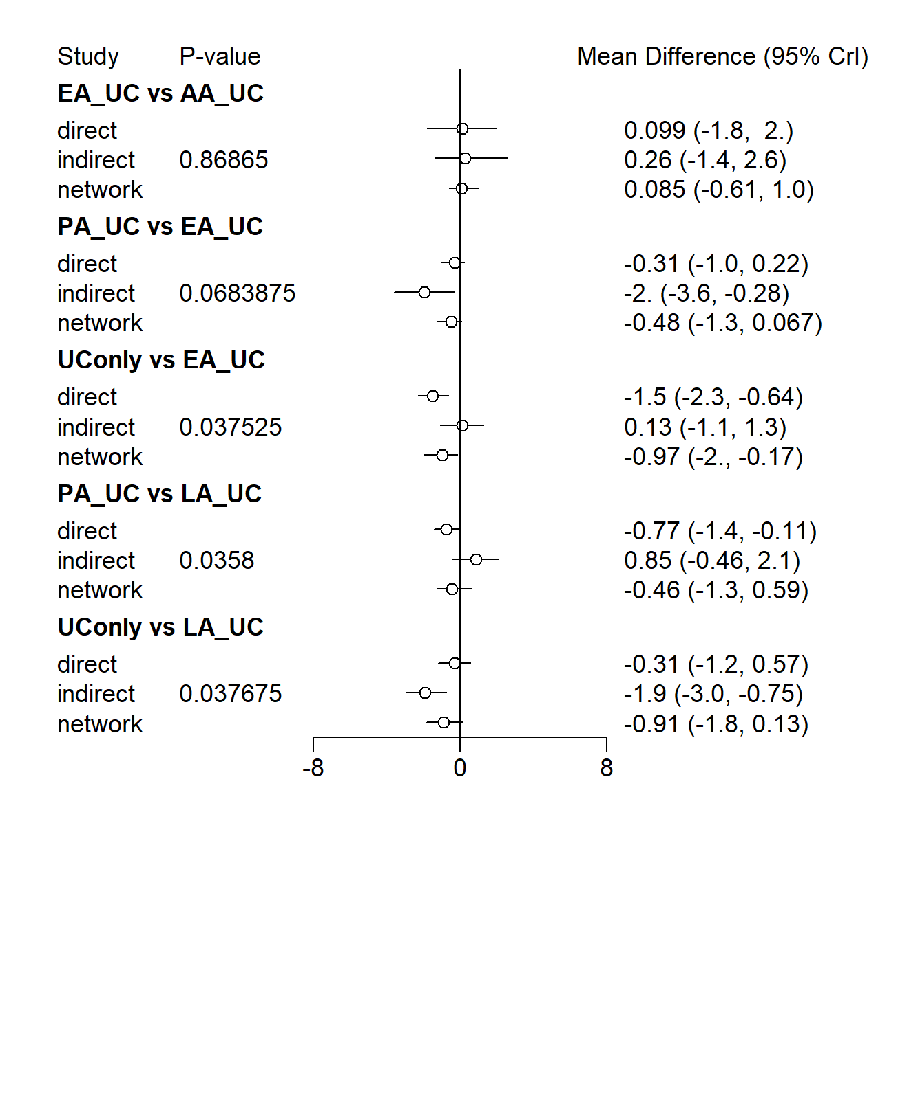
**

**Supplementary Figure 3-(c)** Waist circumference

**
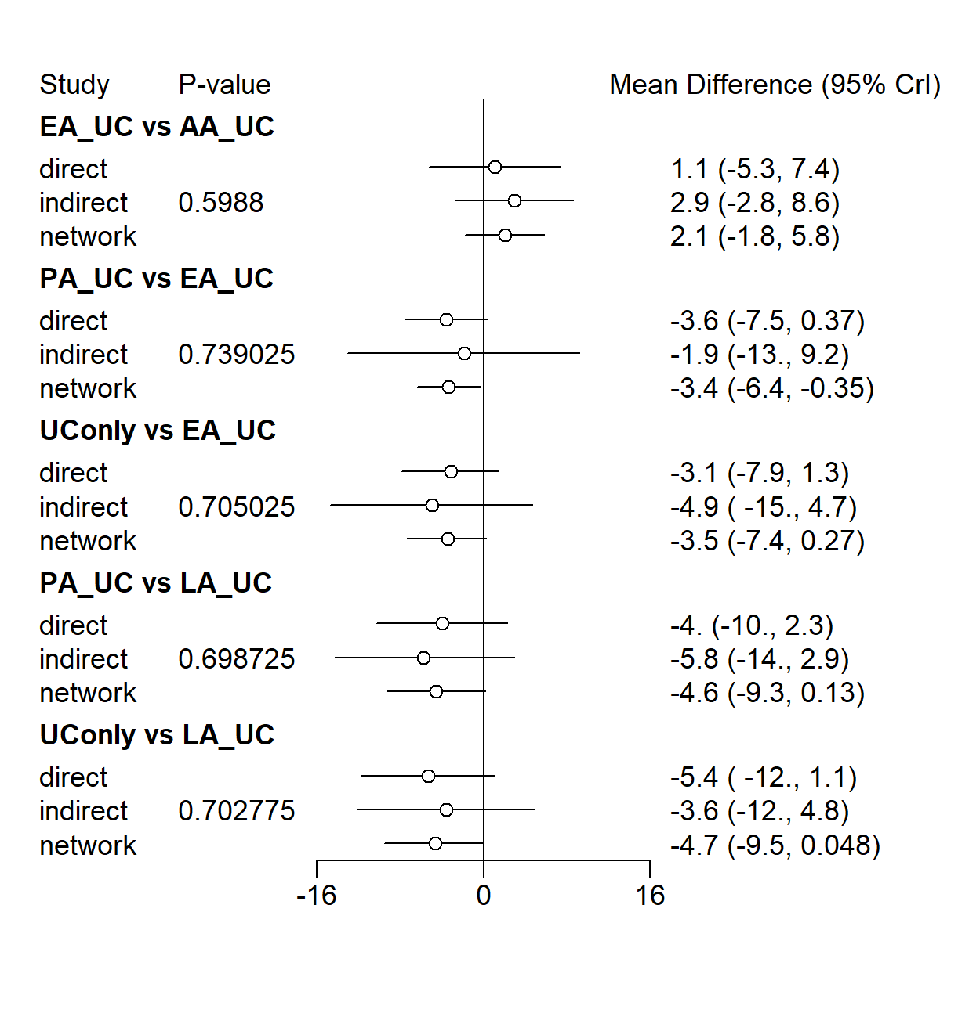
**

**Abbreviations.** AA: Auricular acupuncture; BMI: Body mass index; BW: Body weight; EA: Electroacupuncture; LA: Laser acupuncture; MA: Manual acupuncture; PA: Placebo acupuncture; UC: Usual care; WC: Waist circumference

**Supplementary Figures 4.** Markov Chain Monte Carlo(MCMC) diagnostics

**Supplementary Figures 4-(a)** Body weight


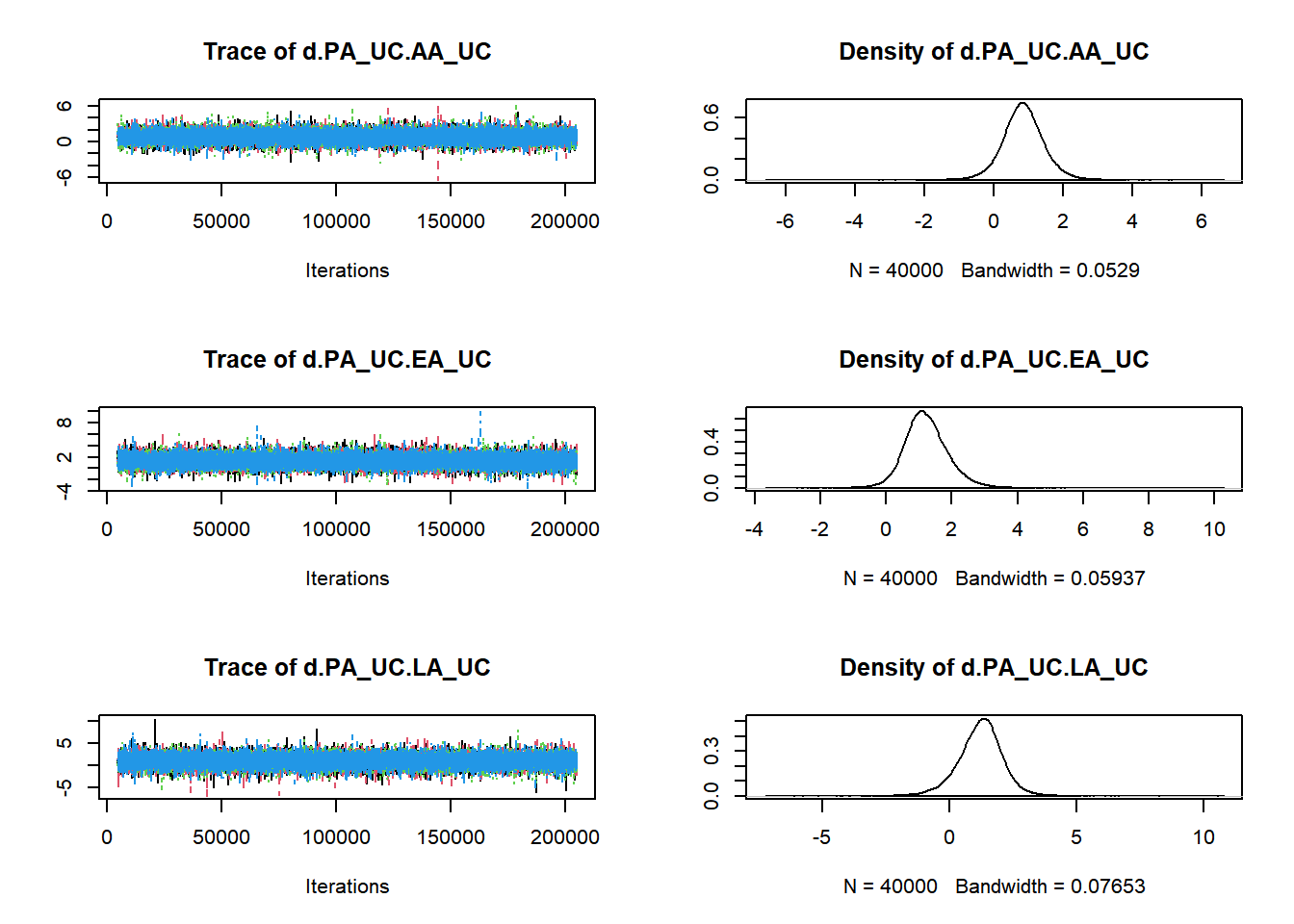

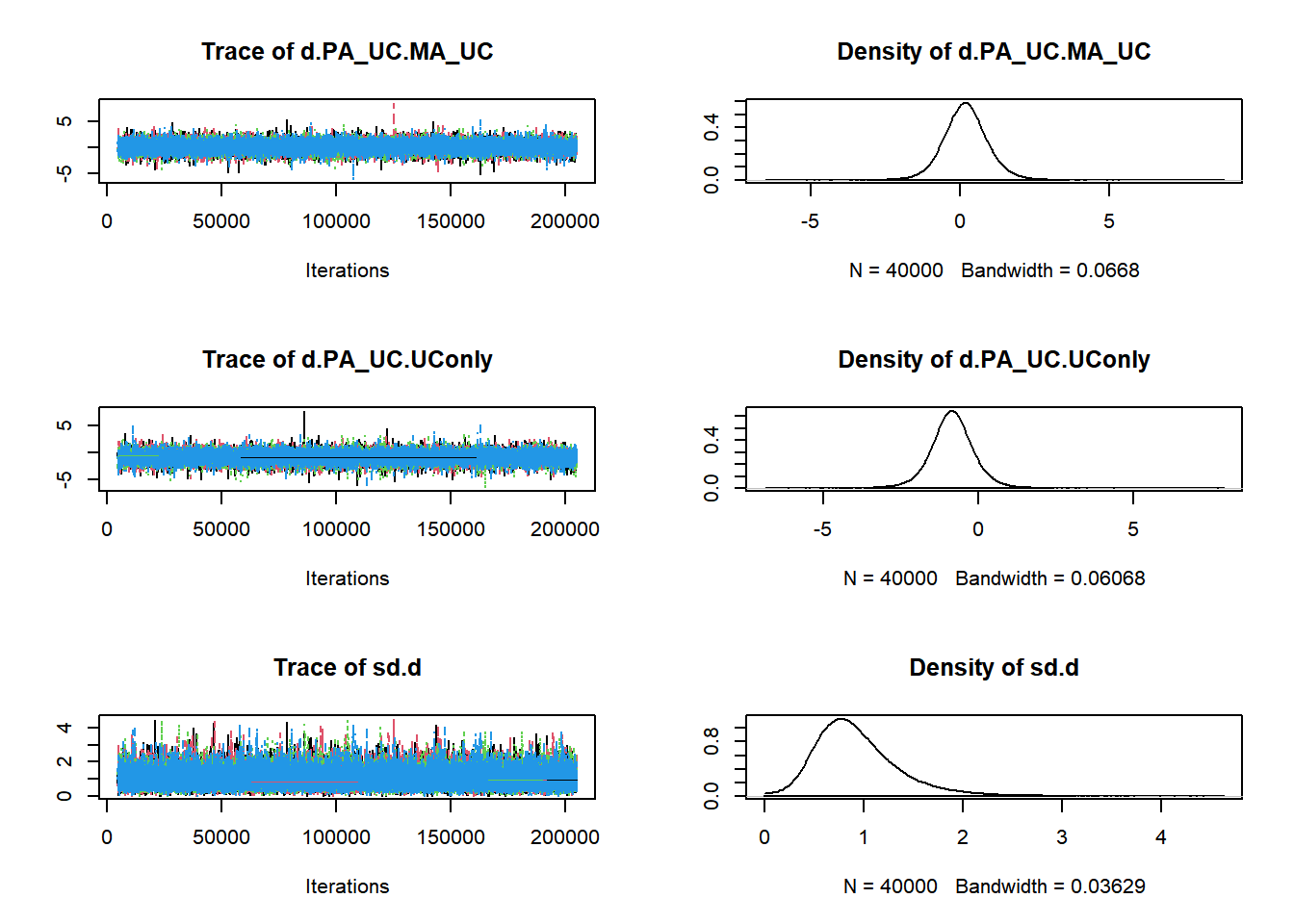


**Supplementary Figures 4-(b)** Body mass index

**
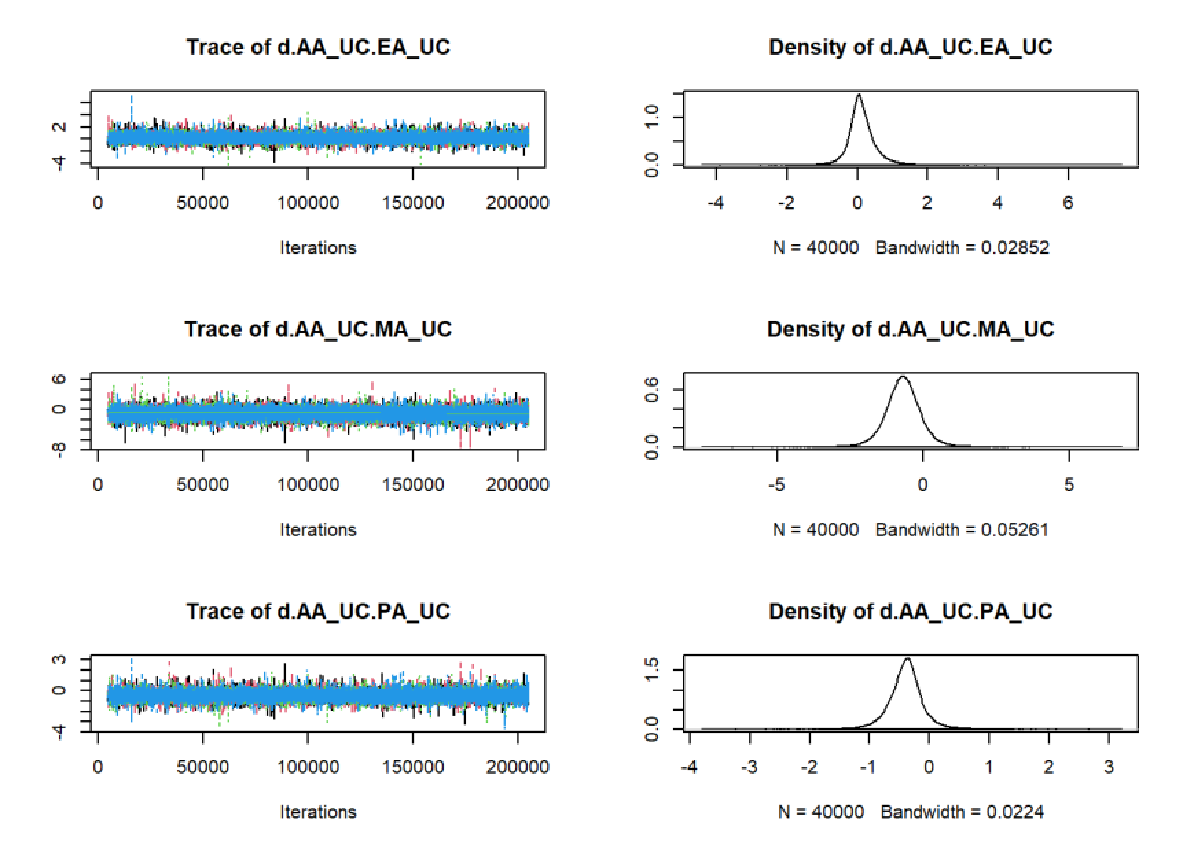
**

**
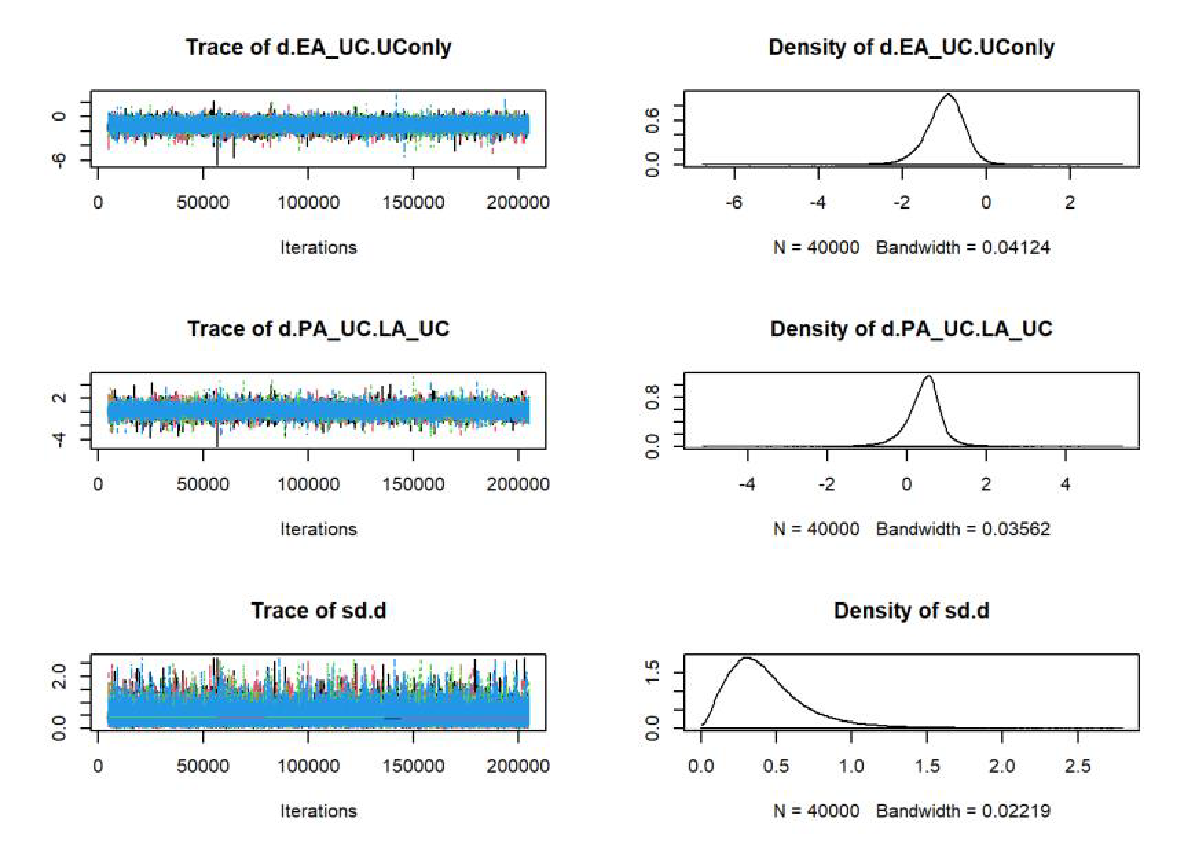
**

**Supplementary Figures 4-(c)** Waist circumference

**
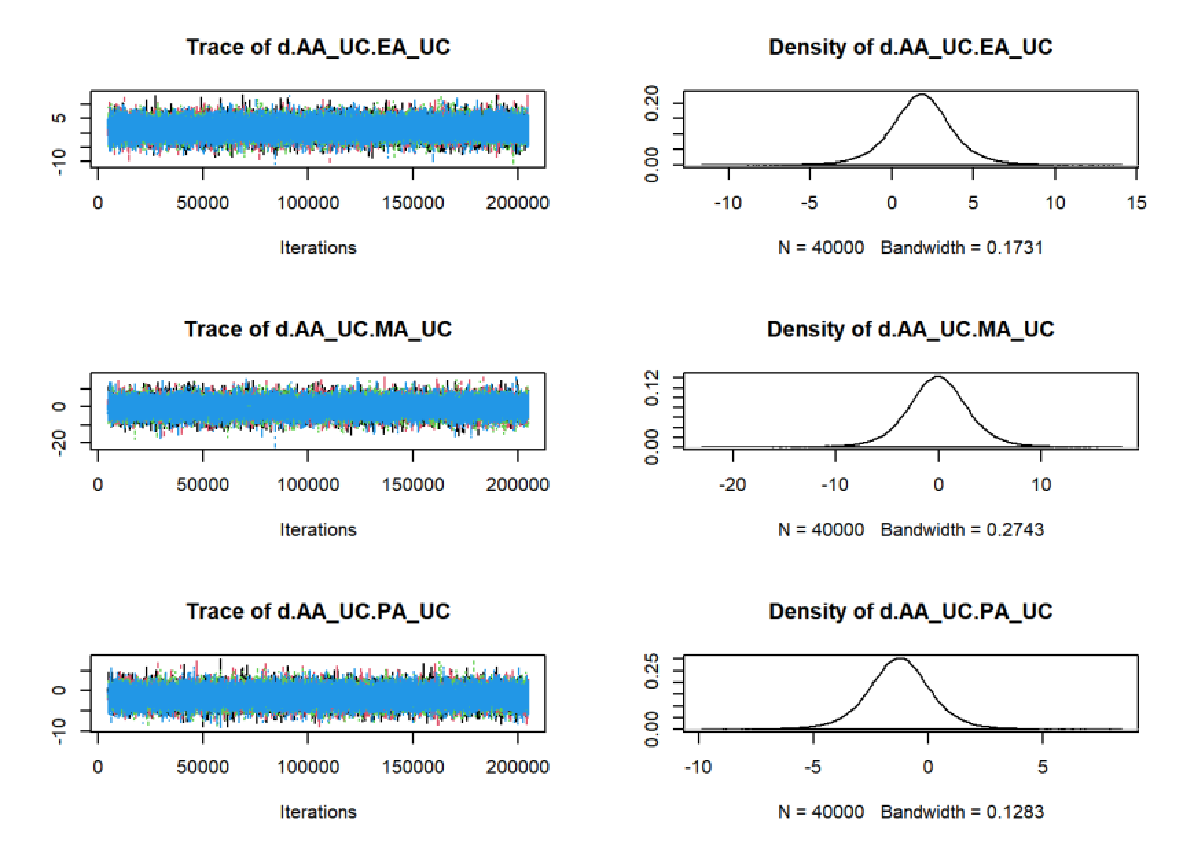
**

**
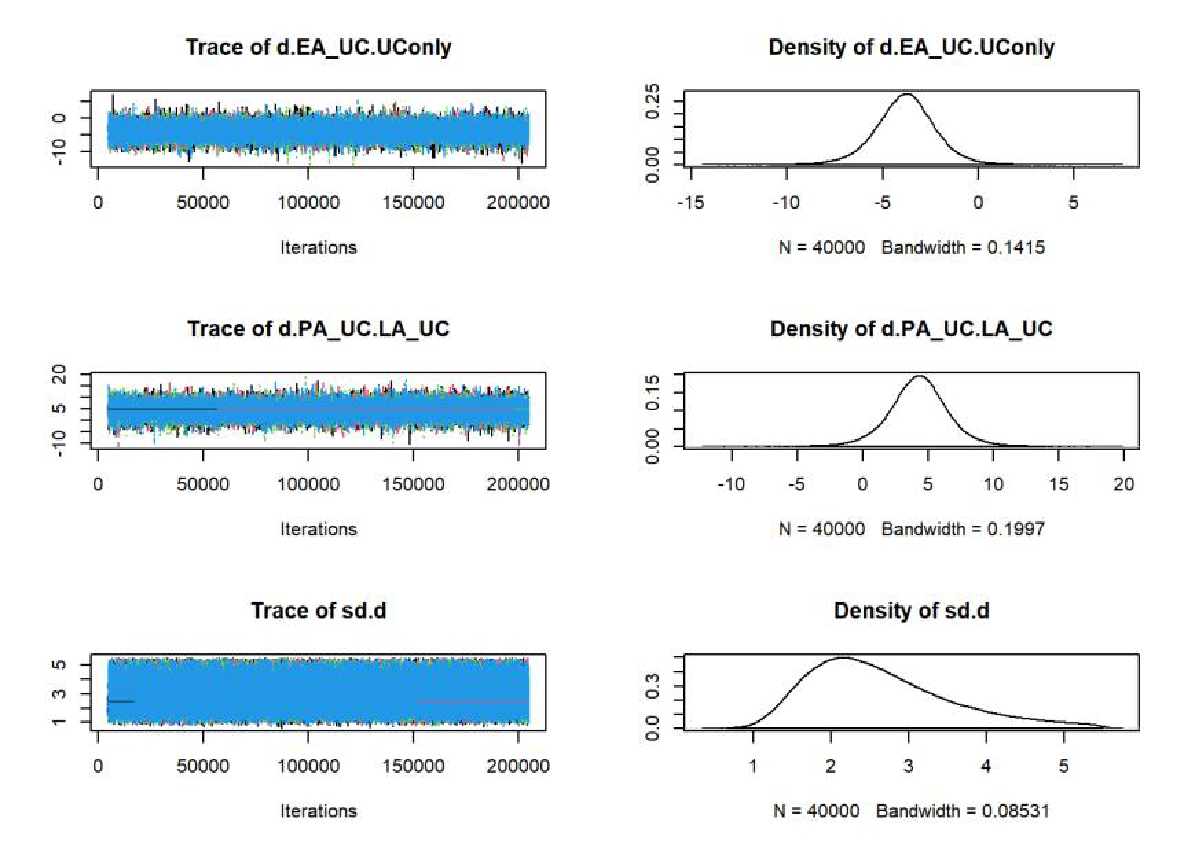
**

**Abbreviations.** AA: Auricular acupuncture; BMI: Body mass index; BW: Body weight; EA: Electroacupuncture; LA: Laser acupuncture; MA: Manual acupuncture; PA: Placebo acupuncture; UC: Usual care; WC: Waist circumference

**Supplementary Figures 5.** Gelman-rubin diagnostics

**Supplementary Figures 5-(a)** Body weight

**
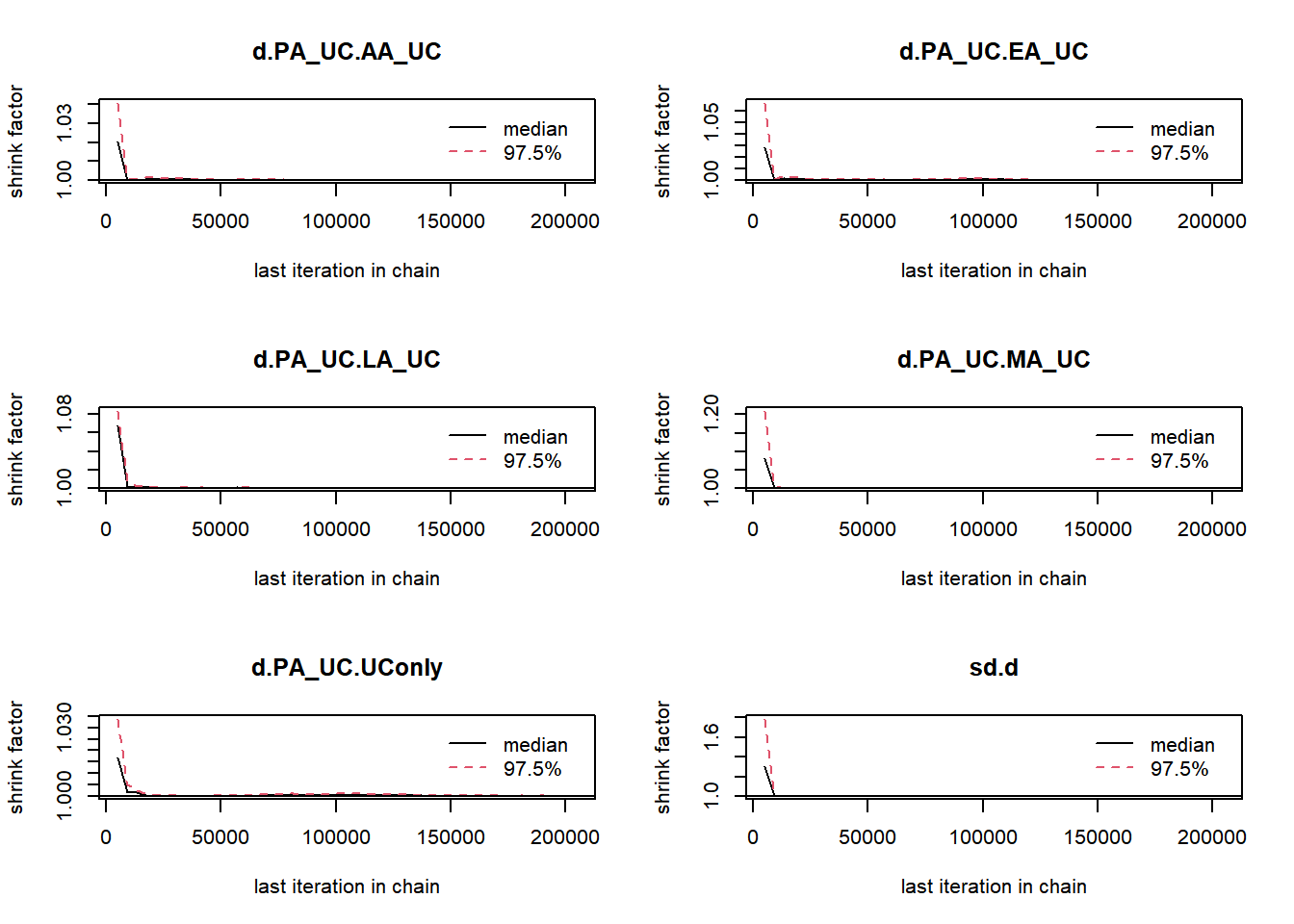
**

**Supplementary Figures 4-(c)** Body mass index

**
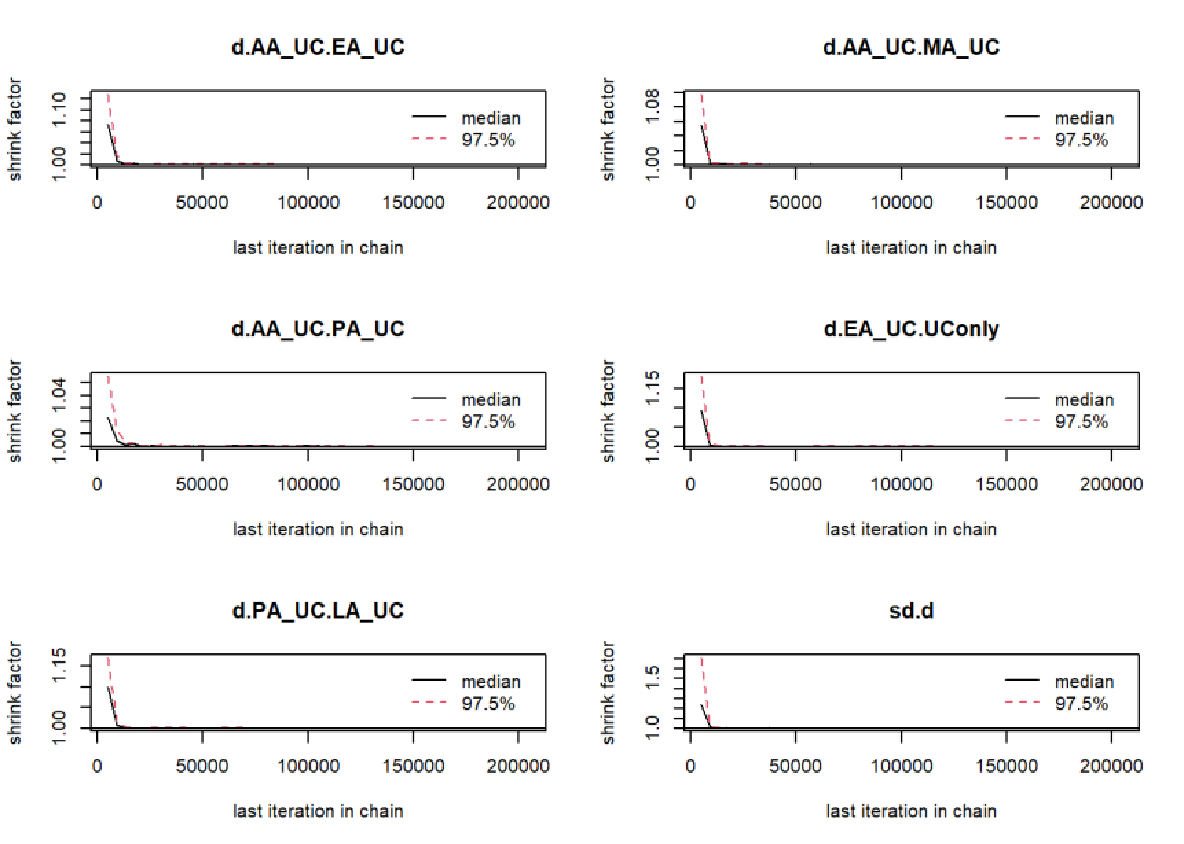
**

**Supplementary Figures 5-(c)** Waist circumference

**
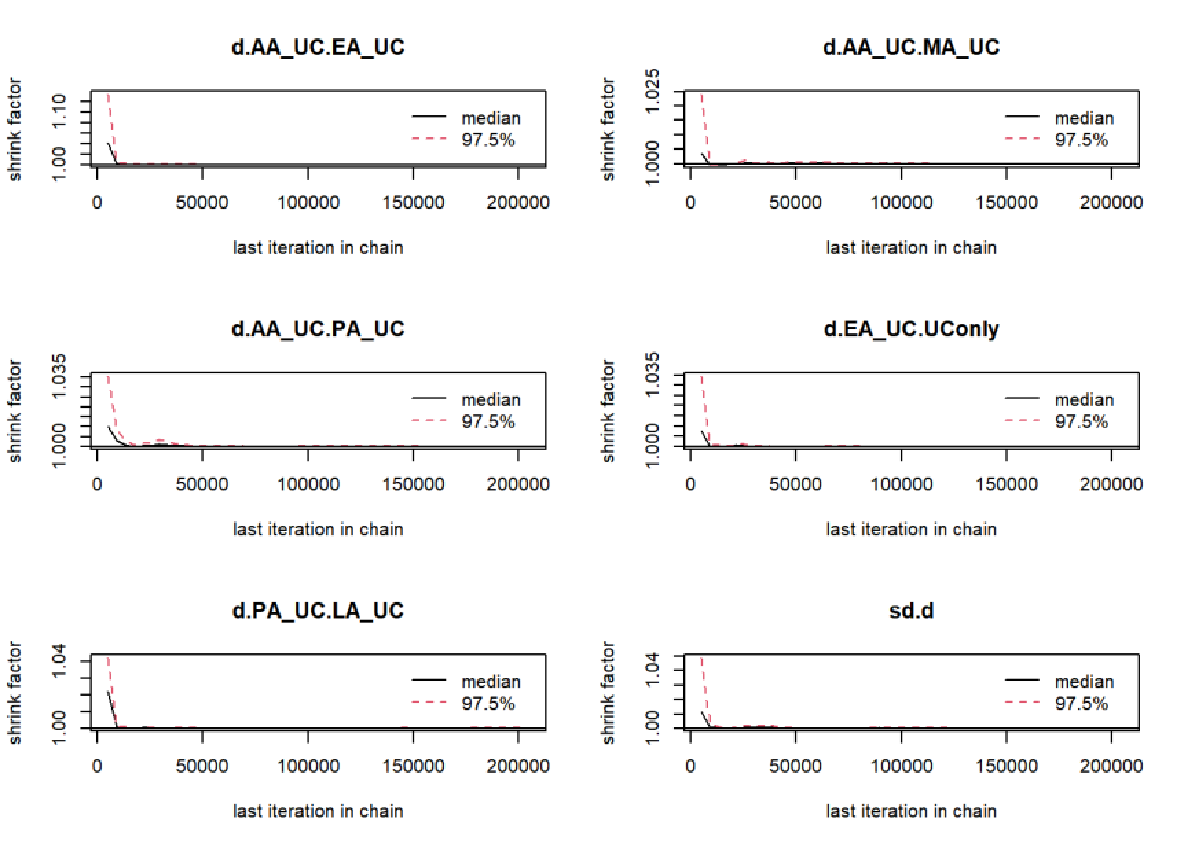
**

**Abbreviations.** AA: Auricular acupuncture; BMI: Body mass index; BW: Body weight; EA: Electroacupuncture; LA: Laser acupuncture; MA: Manual acupuncture; PA: Placebo acupuncture; UC: Usual care; WC: Waist circumference

**Supplementary Figure 6.** Adverse effect rate – forest plot

**Supplementary Figure 6-(a)** acupuncture + usual care vs usual care only

**
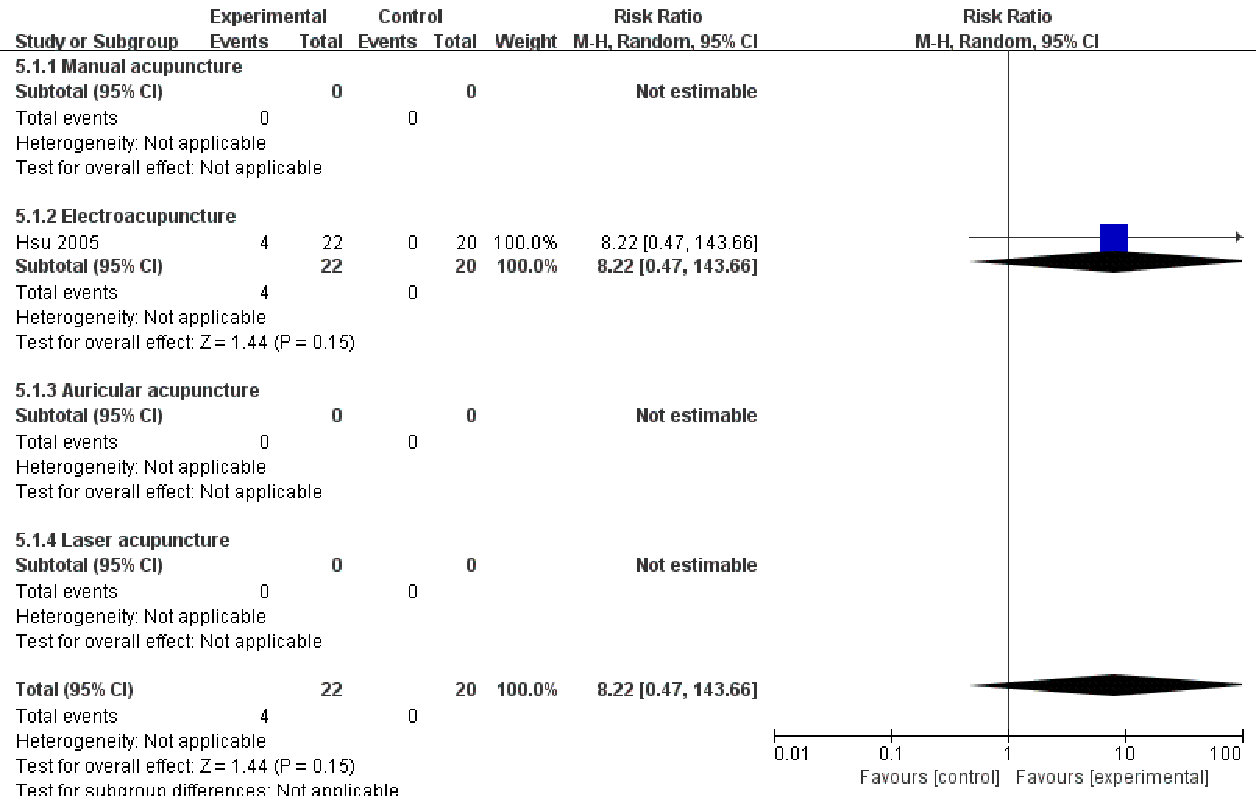
**

**Supplementary Figure 6-(b)** acupuncture + usual care vs placebo acupuncture + usual care

**
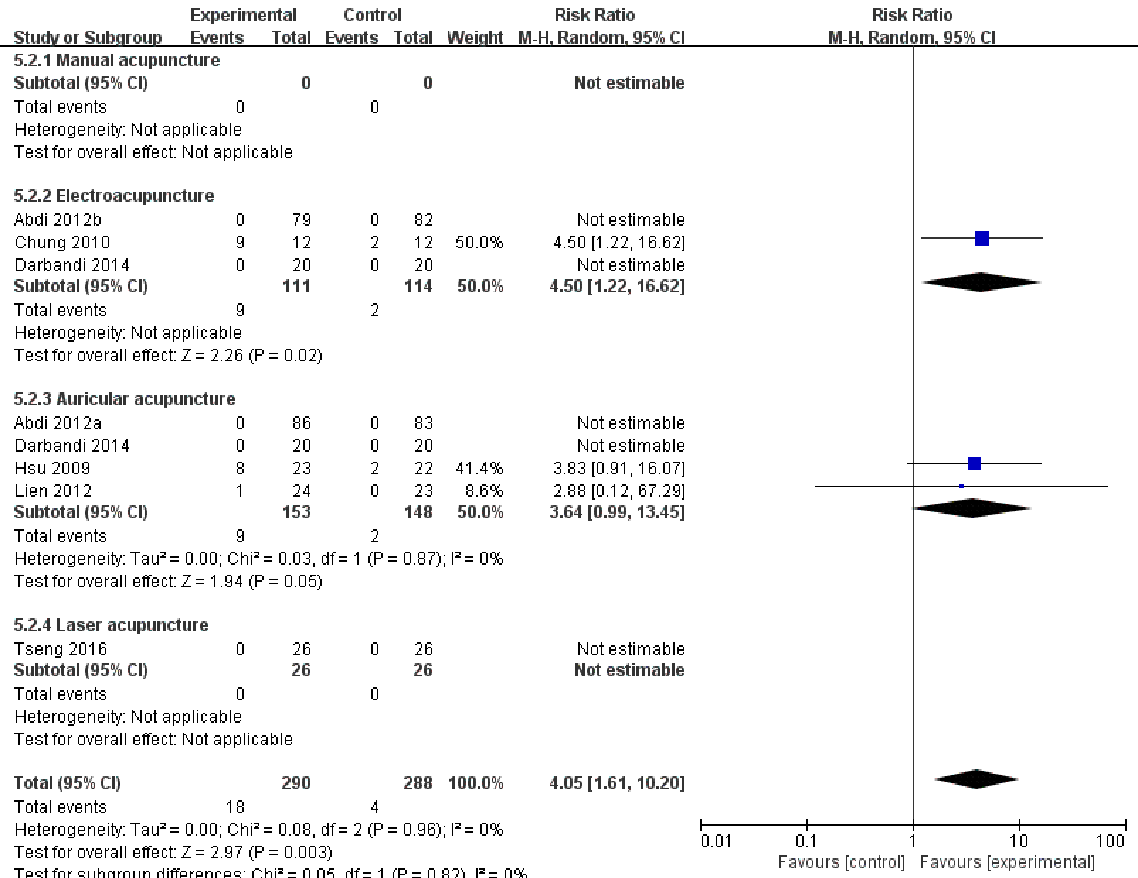
**

**Supplementary Figure 7.** Drop-out rate – forest plot

**Supplementary Figure 7-(a)** acupuncture + usual care vs usual care only

**
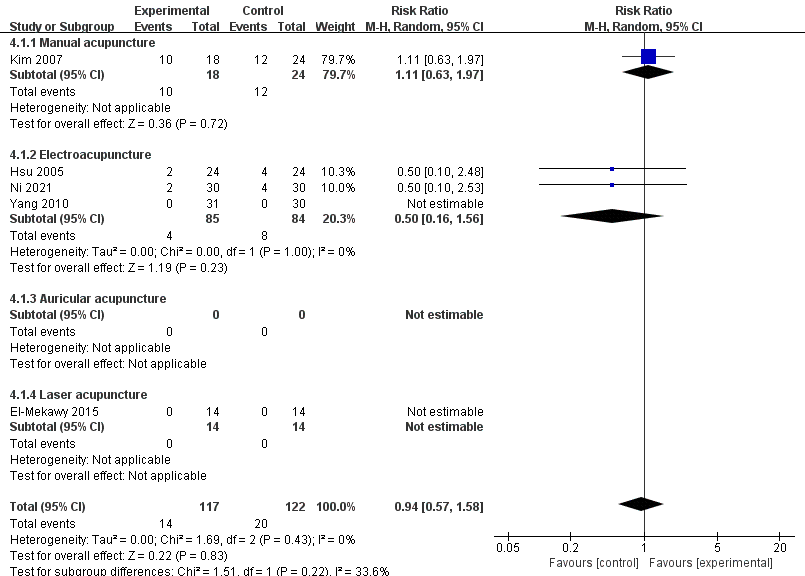
**

**Supplementary Figure 7-(b)** acupuncture + usual care vs placebo acupuncture + usual care

**
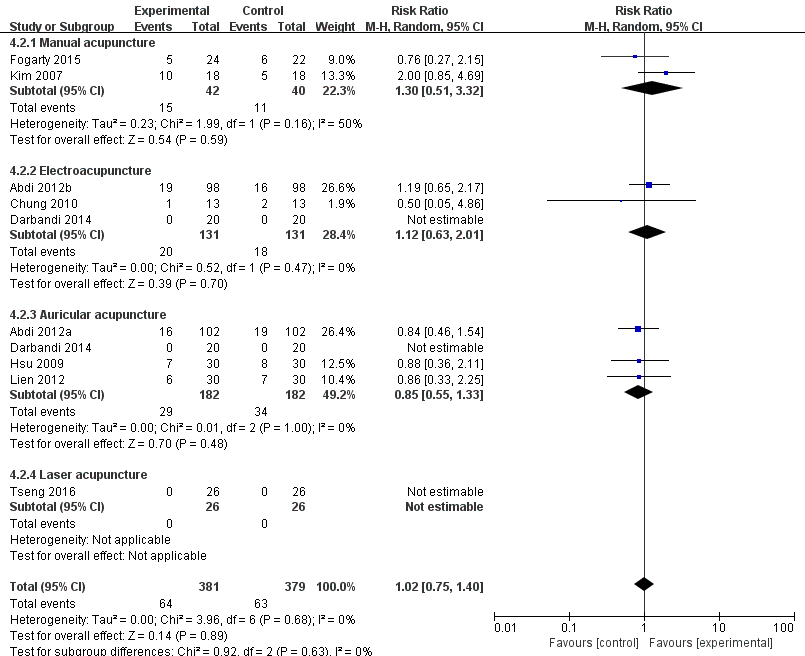
**

**Supplementary Figure 8.** Funnel plot

**Supplementary Figures 8-(a)** Body weight

**
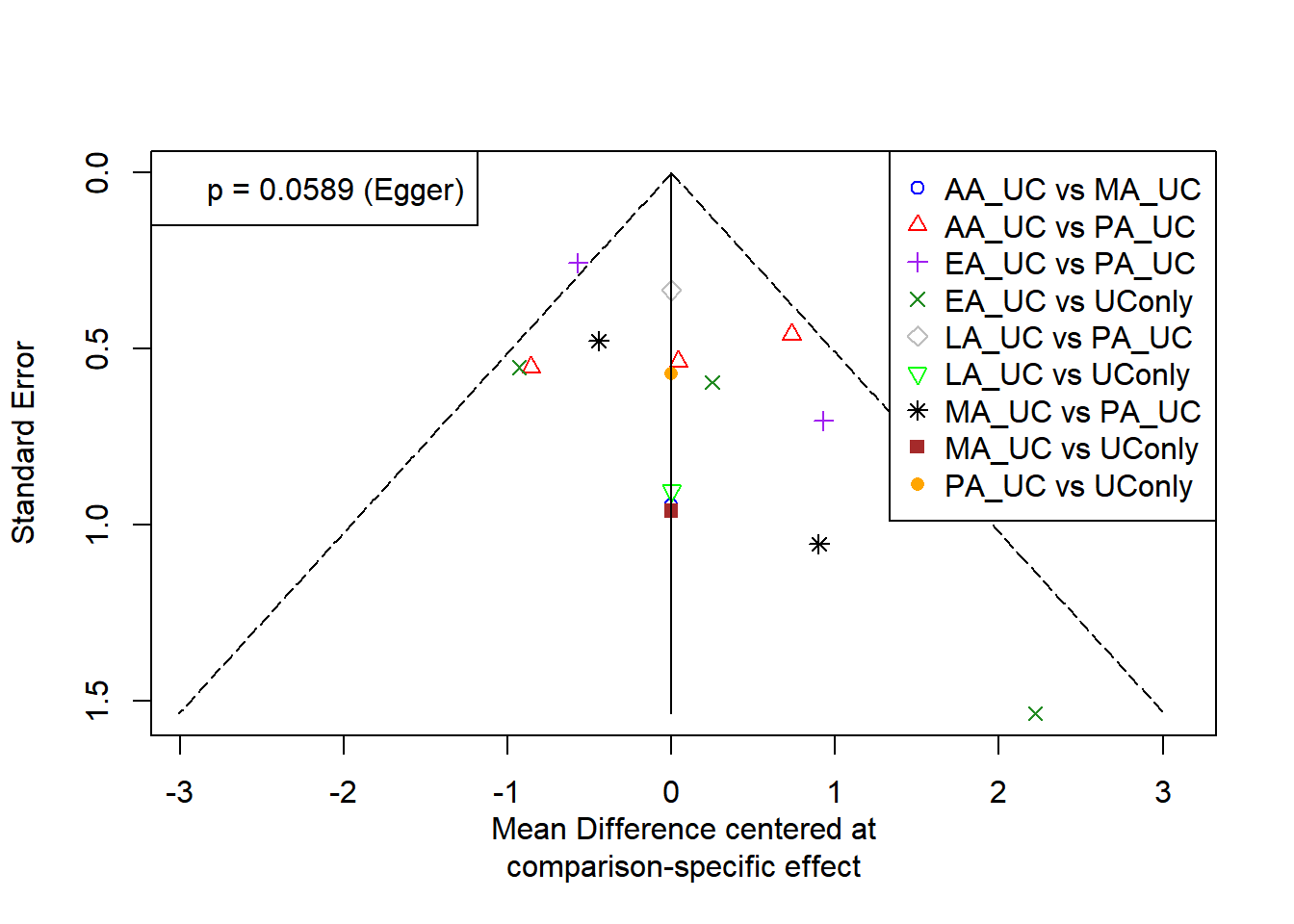
**

**Supplementary Figures 8-(b)** Body mass index

**
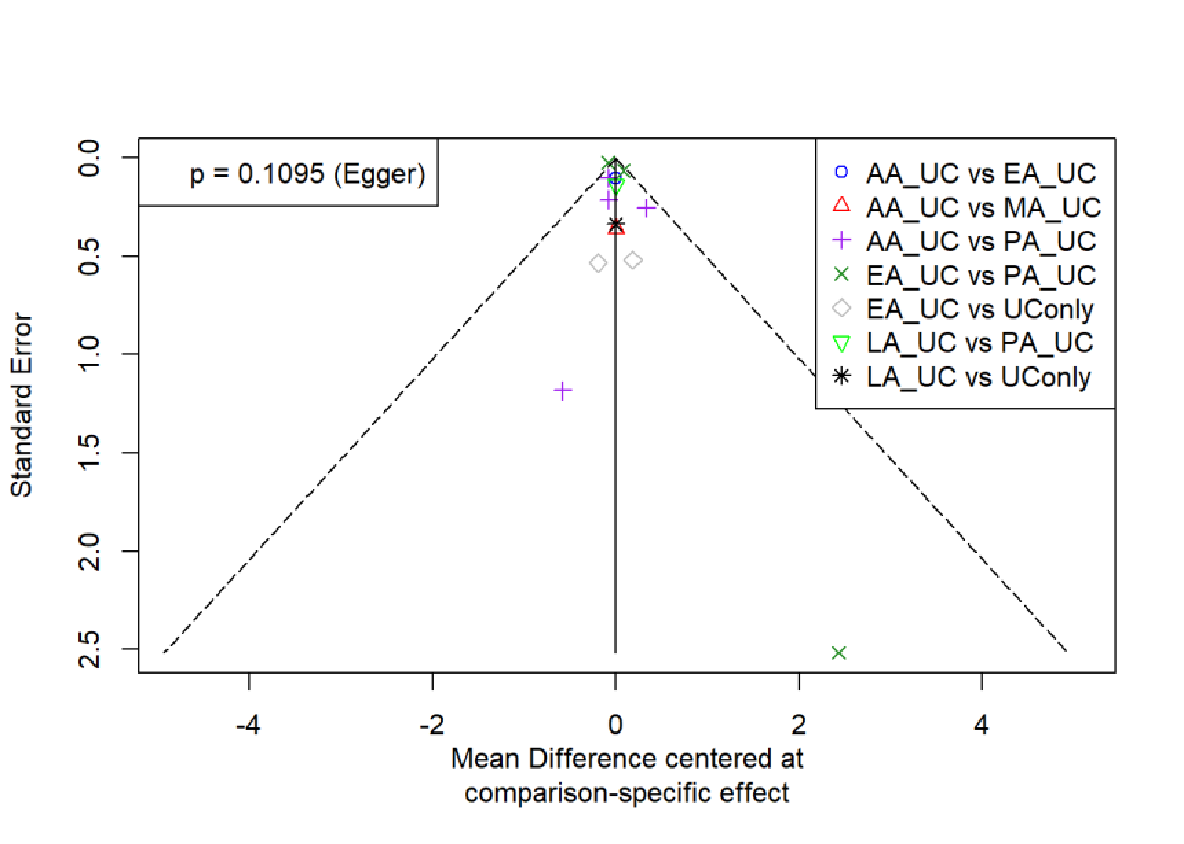
**

**Supplementary Figures 8-(c)** Waist circumference

**
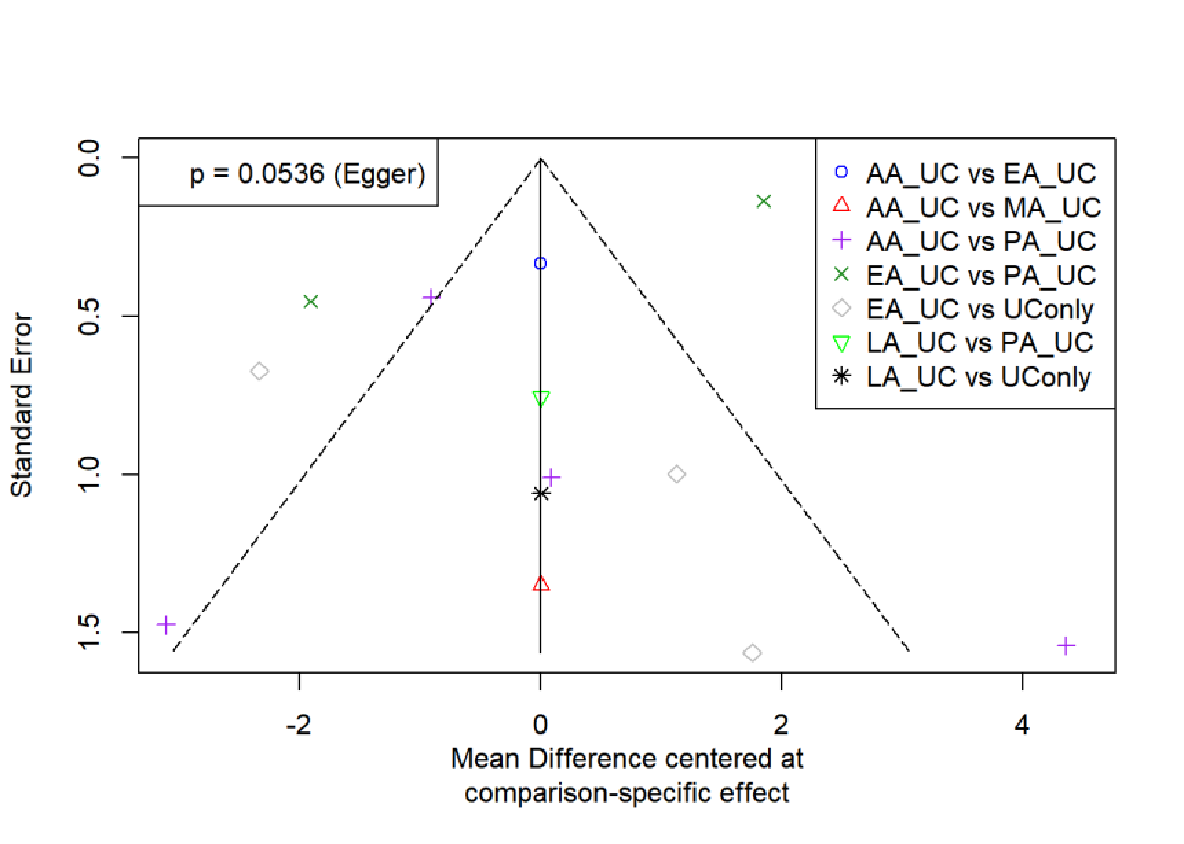
**

**Abbreviations.** AA: Auricular acupuncture; BMI: Body mass index; BW: Body weight; EA: Electroacupuncture; LA: Laser acupuncture; MA: Manual acupuncture; PA: Placebo acupuncture; UC: Usual care; WC: Waist circumference

**Supplementary Table 1. Detailed Search Strategy**

| **Database** | **#** | **Search strategies** | **Results** | |  |
| --- | --- | --- | --- | --- | --- |
| PubMed | #1 | “Obesity”[MH] OR “obesity”[TIAB] OR “Weight loss”[MH] OR “Weight loss”[TIAB] OR “weightloss”[TIAB] OR “overweight”[MH] OR “overweight”[TIAB] OR “Over weight”[TIAB] OR “obese”[TIAB] OR “weight control”[TIAB] OR “weight reduction”[TIAB] OR “Body weight”[MH] OR “body mass index”[TIAB] OR “BMI”[TIAB] | 884309  977248* | |  |
|  | #2 | “Acupuncture Therapy”[MH] OR “Acupuncture Points”[MH] OR “Acupuncture”[MH] OR “Meridians”[MH] OR “acupuncture”[TIAB] OR “acupoint”[TIAB] OR “Acupressure”[MH] OR “Needle”[TIAB] OR “Needling”[TIAB] OR “Needle embedding therapy”[TIAB] OR “dry needling”[MH] OR “trigger point”[TIAB] OR “dry needling”[TIAB] OR “meridian”[TIAB] | 154690  168605* | |  |
|  | #3 | “Electroacupuncture”[MH] OR “electroacupuncture”[TIAB] OR “electro-acupuncture”[TIAB] | 6844  7819* | |  |
|  | #4 | “Scalp acupuncture”[TIAB] OR “head acupuncture”[TIAB] | 296  351* | |  |
|  | #5 | “Acupuncture, Ear”[MH] OR “Auriculotherapy”[MH] OR “ear acupuncture”[TIAB] OR “auricular acupuncture”[TIAB] OR “auricular acupressure”[TIAB] OR “auricular acupoint stimulation”[TIAB] OR “auricular therapy”[TIAB] | 1066  1229* | |  |
|  | #6 | “Pharmacopuncture”[TIAB] OR “Herbal acupuncture”[TIAB] OR “Hydro-acupuncture”[TIAB] | 250  294* | |  |
|  | #7 | “fire acupuncture”[TIAB] OR “fire needling”[TIAB] OR “warm acupuncture”[TIAB] OR “warm needle”[TIAB] OR “Warm needle acupuncture”[TIAB] OR “needle warming moxibustion”[TIAB] | 163  228* | |  |
|  | #8 | “Acupotome”[TIAB] OR “acupotomy”[TIAB] OR “needle knife”[TIAB] OR “cutting needle”[TIAB] OR “needle scalpel”[TIAB] OR “acupotomology”[TIAB] OR “miniscalpel”[TIAB] OR “sword-like needle”[TIAB] | 1261  1389* | |  |
|  | #9 | “catgut”[MH] OR “acupoint catgut embedding”[TIAB] OR “catgut implantation”[TIAB] OR “catgut embedding”[TIAB] OR “thread embedding”[TIAB] OR “thread implantation”[TIAB] | 757  831* | |  |
|  | #10 | “Laser acupuncture”[TIAB] OR “Laser puncture”[TIAB] OR “laser needle”[TIAB] OR “laserpuncture”[TIAB] | 469  526* | |  |
|  | #11 | “Randomized Controlled Trial”[PT] OR “Controlled Clinical Trial”[PT] OR “Randomized Controlled Trial”[TIAB] OR “Controlled Clinical Trial”[TIAB] OR “clinical trial”[TIAB] OR “random”[TIAB] OR “randomized”[TIAB] OR “placebo”[TIAB] OR “Clinical Trials as Topic”[MH] OR “randomly”[TIAB] OR “trial”[TIAB] OR “clinical research”[TIAB] | 2082459  2300645* | |  |
|  | #12 | "animals"[MH] NOT "humans"[MH] | 5030887  5217662* | |  |
|  | #13 | #2 OR #3 OR #4 OR #5 OR #6 OR #7 OR #8 OR #9 OR #10 | 155868  169996* | |  |
|  | #14 | (#1 AND #11 AND #13) NOT #12 | 662  759* | |  |
| CENTRAL | #1 | (“Obesity”):ti,ab,kw OR (“Weight loss”):ti,ab,kw OR (“weightloss”):ti,ab,kw OR (“overweight”):ti,ab,kw OR (“Over weight”):ti,ab,kw | 56996  67257* | |  |
|  | #2 | ("obese"):ti,ab,kw OR ("weight control"):ti,ab,kw OR ("weight reduction"):ti,ab,kw OR ("body mass index"):ti,ab,kw | 63940  73614* | |  |
|  | #3 | (“BMI”):ti,ab,kw | 46897  59852* | |  |
|  | #4 | MeSH descriptor: [Obesity] explode all trees | 15961  21365* | |  |
|  | #5 | MeSH descriptor: [Weight Loss] explode all trees | 7161  9056* | |  |
|  | #6 | MeSH descriptor: [Overweight] explode all trees | 19085  25047* | |  |
|  | #7 | MeSH descriptor: [Body Weight] explode all trees | 31404  40516* | |  |
|  | #8 | ("acupuncture"):ti,ab,kw OR ("acupoint"):ti,ab,kw OR ("Needle"):ti,ab,kw OR ("Needling"):ti,ab,kw OR ("Needle embedding therapy"):ti,ab,kw | 32641  39756* | |  |
|  | #9 | (“trigger point”):ti,ab,kw OR (“dry needling”):ti,ab,kw OR (“meridian”):ti,ab,kw | 2569  3359* | |  |
|  | #10 | MeSH descriptor: [Acupuncture Therapy] explode all trees | 5326  7057* | |  |
|  | #11 | MeSH descriptor: [Acupuncture Points] explode all trees | 2261  2750* | |  |
|  | #12 | MeSH descriptor: [Acupuncture] explode all trees | 164  224* | |  |
|  | #13 | MeSH descriptor: [Meridians] explode all trees | 2306  2822* | |  |
|  | #14 | MeSH descriptor: [Acupressure] explode all trees | 427  585* | |  |
|  | #15 | MeSH descriptor: [Dry Needling] explode all trees | 101  173* | |  |
|  | #16 | (“electroacupuncture”):ti,ab,kw OR (“electro-acupuncture”):ti,ab,kw | 2912  3657* | |  |
|  | #17 | MeSH descriptor: [Electroacupuncture] explode all trees | 889  1162* | |  |
|  | #18 | (“Scalp acupuncture”):ti,ab,kw OR (“head acupuncture”):ti,ab,kw | 325  390* | |  |
|  | #19 | (“ear acupuncture”):ti,ab,kw OR (“auricular acupuncture”):ti,ab,kw OR (“auricular acupressure”):ti,ab,kw OR (“auricular acupoint stimulation”):ti,ab,kw OR (“auricular therapy”):ti,ab,kw | 846  1097* | |  |
|  | #20 | MeSH descriptor: [Acupuncture, Ear] explode all trees | 221  263* | |  |
|  | #21 | MeSH descriptor: [Auriculotherapy] explode all trees | 260  323* | |  |
|  | #22 | (“Pharmacopuncture”):ti,ab,kw OR (“Herbal acupuncture”):ti,ab,kw OR (“Hydro-acupuncture”):ti,ab,kw | 103  127* | |  |
|  | #23 | (“fire acupuncture”):ti,ab,kw OR (“fire needling”):ti,ab,kw OR (“warm acupuncture”):ti,ab,kw OR (“warm needle”):ti,ab,kw OR (“needle warming moxibustion”):ti,ab,kw | 149  212* | |  |
|  | #24 | (“Acupotome”):ti,ab,kw OR (“acupotomy”):ti,ab,kw OR (“needle knife”):ti,ab,kw OR (“cutting needle”):ti,ab,kw OR (“needle scalpel”):ti,ab,kw | 310  391* | |  |
|  | #25 | (“acupotomology”):ti,ab,kw OR (“miniscalpel”):ti,ab,kw OR (“sword-like needle”):ti,ab,kw | 24  31* | |  |
|  | #26 | (“acupoint catgut embedding”):ti,ab,kw OR (“catgut implantation”):ti,ab,kw OR (“catgut embedding”):ti,ab,kw OR (“thread embedding”):ti,ab,kw OR (“thread implantation”):ti,ab,kw | 304  411* | |  |
|  | #27 | MeSH descriptor: [Catgut] explode all trees | 118  128* | |  |
|  | #28 | (“Laser acupuncture”):ti,ab,kw OR (“Laser puncture”):ti,ab,kw OR (“laser needle”):ti,ab,kw OR (“laserpuncture”):ti,ab,kw | 406  508* | |  |
|  | #29 | #1 OR #2 OR #3 OR #4 OR #5 OR #6 OR #7 | 115443  139813* | |  |
|  | #30 | #8 OR #9 OR #10 OR #11 OR #12 OR #13 OR #14 OR #15 OR #16 OR #17 OR #18 OR #19 OR #20 OR #21 OR #22 OR #23 OR #24 OR #25 OR #26 OR #27 OR #28 | 35035  42874* | |  |
|  | #31 | #29 AND #30 | 1458  2062* | |  |
| EMBASE via Elsevier | #1 | ("Obesity"):ti,ab,kw OR ("Weight loss"):ti,ab,kw OR ("weightloss"):ti,ab,kw OR ("overweight"):ti,ab,kw OR ("Over weight"):ti,ab,kw OR ("obese"):ti,ab,kw OR ("weight control"):ti,ab,kw OR ("weight reduction"):ti,ab,kw OR ("body mass index"):ti,ab,kw OR ("BMI"):ti,ab,kw OR ("Obesity")/exp OR ("Weight Loss")/exp OR ("Overweight")/exp OR ("Body Weight")/exp | 1672073  1906852* | |  |
|  | #2 | ("acupuncture"):ti,ab,kw OR ("acupoint"):ti,ab,kw OR ("Needle"):ti,ab,kw OR ("Needling"):ti,ab,kw OR ("Needle embedding therapy"):ti,ab,kw OR ("trigger point"):ti,ab,kw OR ("dry needling"):ti,ab,kw OR ("meridian"):ti,ab,kw OR ("Acupuncture Therapy")/exp OR ("Acupuncture Points")/exp OR ("Acupuncture")/exp OR ("Meridians")/exp OR ("Acupressure")/exp OR ("Dry Needling")/exp | 231638  253870* | |  |
|  | #3 | (“electroacupuncture”):ti,ab,kw OR (“electro-acupuncture”):ti,ab,kw OR (“Electroacupuncture”)/exp | 9737  11306* | |  |
|  | #4 | (“Scalp acupuncture”):ti,ab,kw OR (“head acupuncture”):ti,ab,kw | 460  523* | |  |
|  | #5 | ("ear acupuncture"):ti,ab,kw OR ("auricular acupuncture"):ti,ab,kw OR ("auricular acupressure"):ti,ab,kw OR ("auricular acupoint stimulation"):ti,ab,kw OR ("auricular therapy"):ti,ab,kw OR ("Acupuncture, Ear")/exp OR ("Auriculotherapy")/exp | 1600  1902* | |  |
|  | #6 | (“Pharmacopuncture”):ti,ab,kw OR (“Herbal acupuncture”):ti,ab,kw OR (“Hydro-acupuncture”):ti,ab,kw | 357  405* | |  |
|  | #7 | (“fire acupuncture”):ti,ab,kw OR (“fire needling”):ti,ab,kw OR (“warm acupuncture”):ti,ab,kw OR (“warm needle”):ti,ab,kw OR (“needle warming moxibustion”):ti,ab,kw | 212  279* | |  |
|  | #8 | (“Acupotome”):ti,ab,kw OR (“acupotomy”):ti,ab,kw OR (“needle knife”):ti,ab,kw OR (“cutting needle”):ti,ab,kw OR (“needle scalpel”):ti,ab,kw OR (“acupotomology”):ti,ab,kw OR (“miniscalpel”):ti,ab,kw OR (“sword-like needle”):ti,ab,kw | 2359  2557* | |  |
|  | #9 | (“acupoint catgut embedding”):ti,ab,kw OR (“catgut implantation”):ti,ab,kw OR (“catgut embedding”):ti,ab,kw OR (“thread embedding”):ti,ab,kw OR (“thread implantation”):ti,ab,kw OR (“Catgut”)/exp | 1103  1204* | |  |
|  | #10 | (“Laser acupuncture”):ti,ab,kw OR (“Laser puncture”):ti,ab,kw OR (“laser needle”):ti,ab,kw OR (“laserpuncture”):ti,ab,kw | 679  744* | |  |
|  | #11 | (“Randomized Controlled Trial”):it OR (“controlled clinical trial”):it OR (“Randomized Controlled Trial”):it OR (“Controlled Clinical Trial”):ti,ab,kw OR (“clinical trial”):ti,ab,kw OR (“random”):ti,ab,kw OR (“randomized”):ti,ab,kw OR (“placebo”):it OR (“randomly”):ti,ab,kw OR (“trial”):ti,ab,kw OR (“clinical research”):ti,ab,kw OR (“Clinical Trials as Topic”)/exp | 2511208  2848364* | |  |
|  | #12 | [animals]/lim NOT [humans]/lim | 6186971  6503788* | |  |
|  | #13 | #2 OR #3 OR #4 OR #5 OR #6 OR #7 OR #8 OR #9 OR #10 | 232622  254913* | |  |
|  | #14 | #1 AND #11 AND #13 | 1701  1978* | |  |
|  | #15 | #14 NOT #12 | 1493  1732* | |  |
| CINAHL | #1 | TI Obesity OR AB Obesity OR TI Weight loss OR AB Weight loss OR TI overweight OR AB overweight OR TI weightloss OR AB weightloss OR TI over weight OR AB over weight OR TI obese OR AB obese OR TI weight control OR AB weight control OR TI Body weight OR AB Body weight OR TI weight reduction OR AB weight reduction OR TI body mass OR AB body mass OR TI body mass index OR AB body mass index OR TI BMI OR AB BMI | 87653  85971* | |  |
|  | #2 | TI Acupuncture Therapy OR AB Acupuncture Therapy OR TI Acupuncture Points OR AB Acupuncture Points OR TI Acupuncture OR AB Acupuncture OR TI Meridians OR AB Meridians OR TI acupoint OR AB acupoint OR TI acupressure OR AB acupressure OR TI Needle OR AB Needle OR TI Needling OR AB Needling OR TI Needle embedding therapy OR AB Needle embedding therapy OR TI dry needling OR AB dry needling OR TI trigger point OR AB trigger point OR TI meridian OR AB meridian | 13486  14459* | |  |
|  | #3 | TI Electroacupuncture OR AB Electroacupuncture OR TI electro-acupuncture OR AB electro-acupuncture | 859  1001* | |  |
|  | #4 | TI Scalp acupuncture OR AB Scalp acupuncture OR TI head acupuncture OR AB head acupuncture | 44  66* | |  |
|  | #5 | TI Acupuncture, Ear OR AB Acupuncture, Ear OR TI Auriculotherapy OR AB Auriculotherapy OR TI ear acupuncture OR AB ear acupuncture OR TI auricular acupuncture OR AB auricular acupuncture OR TI auricular acupressure OR AB auricular acupressure OR TI auricular acupoint stimulation OR AB auricular acupoint stimulation OR TI auricular therapy OR AB auricular therapy | 206  280* | |  |
|  | #6 | TI Pharmacopuncture OR AB Pharmacopuncture OR TI Herbal acupuncture OR AB Herbal acupuncture OR TI Hydro-acupuncture OR AB Hydro-acupuncture | 41  238* | |  |
|  | #7 | TI fire acupuncture OR AB thermal OR TI fire needling OR AB fire needling OR TI warm acupuncture OR AB warm acupuncture OR TI warm needle OR AB warm needle OR TI Warm needle acupuncture OR AB Warm needle acupuncture OR TI needle warming moxibustion OR AB needle warming moxibustion | 3601  3776* | |  |
|  | #8 | TI Acupotome OR AB Acupotome OR TI acupotomy OR AB acupotomy OR TI needle knife OR AB needle knife OR TI cutting needle OR AB cutting needle OR TI needle scalpel OR AB neelde scalpel OR TI acupotomology OR AB acupotomology OR TI miniscalpel OR AB miniscalpel OR TI sword-like needle OR AB sword-like needle | 122  132* | |  |
|  | #9 | TI catgut OR AB catgut OR TI acupoint catgut embedding OR AB acupoint catgut embedding OR TI catgut implantation OR AB catgut implantation OR TI catgut embedding OR AB catgut embedding OR TI thread embedding OR AB thread embedding OR thread implantation OR thread implantation | 48  58* | |  |
|  | #10 | TI Laser acupuncture OR AB Laser acupuncture OR TI Laser puncture OR AB Laser puncture OR TI laser needle OR AB laser needle OR TI laserpuncture OR AB laserpuncture | 93  152* | |  |
|  | #11 | TI Randomized Controlled Trial OR AB Randomized Controlled Trial OR TI Controlled Clinical Trial OR AB Controlled Clinical Trial OR TI clinical trial OR AB clinical trial OR TI random OR AB random OR randomized OR randomized OR placebo OR placebo OR TI Clinical Trials as Topic OR AB Clinical Trials as Topic OR TI randomly OR AB randomly OR TI trial OR AB trial OR TI groups OR AB groups OR TI clinical research OR AB clinical research | 471617  510128 | |  |
|  | #12 | S2 OR S3 OR S4 OR S5 OR S6 OR S7 OR S8 OR S9 OR S10 | 17340  18533 | |  |
|  | #13 | S1 AND S11 AND S12 | 205  226* | |  |
| AMED | #1 | TI Obesity OR AB Obesity OR TI Weight loss OR AB Weight loss OR TI overweight OR AB overweight OR TI weightloss OR AB weightloss OR TI over weight OR AB over weight OR TI obese OR AB obese OR TI weight control OR AB weight control OR TI Body weight OR AB Body weight OR TI weight reduction OR AB weight reduction OR TI body mass OR AB body mass OR TI body mass index OR AB body mass index OR TI BMI OR AB BMI | | 6741  9154* | |
|  | #2 | TI Acupuncture Therapy OR AB Acupuncture Therapy OR TI Acupuncture Points OR AB Acupuncture Points OR TI Acupuncture OR AB Acupuncture OR TI Meridians OR AB Meridians OR TI acupoint OR AB acupoint OR TI acupressure OR AB acupressure OR TI Needle OR AB Needle OR TI Needling OR AB Needling OR TI Needle embedding therapy OR AB Needle embedding therapy OR TI dry needling OR AB dry needling OR TI trigger point OR AB trigger point OR TI meridian OR AB meridian |  | |  |
|  | #3 | TI Electroacupuncture OR AB Electroacupuncture OR TI electro-acupuncture OR AB electro-acupuncture |  | |  |
|  | #4 | TI Scalp acupuncture OR AB Scalp acupuncture OR TI head acupuncture OR AB head acupuncture |  | |  |
|  | #5 | TI Acupuncture, Ear OR AB Acupuncture, Ear OR TI Auriculotherapy OR AB Auriculotherapy OR TI ear acupuncture OR AB ear acupuncture OR TI auricular acupuncture OR AB auricular acupuncture OR TI auricular acupressure OR AB auricular acupressure OR TI auricular acupoint stimulation OR AB auricular acupoint stimulation OR TI auricular therapy OR AB auricular therapy |  | |  |
|  | #6 | TI Pharmacopuncture OR AB Pharmacopuncture OR TI Herbal acupuncture OR AB Herbal acupuncture OR TI Hydro-acupuncture OR AB Hydro-acupuncture |  | |  |
|  | #7 | TI fire acupuncture OR AB thermal OR TI fire needling OR AB fire needling OR TI warm acupuncture OR AB warm acupuncture OR TI warm needle OR AB warm needle OR TI Warm needle acupuncture OR AB Warm needle acupuncture OR TI needle warming moxibustion OR AB needle warming moxibustion |  | |  |
|  | #8 | TI Acupotome OR AB Acupotome OR TI acupotomy OR AB acupotomy OR TI needle knife OR AB needle knife OR TI cutting needle OR AB cutting needle OR TI needle scalpel OR AB neelde scalpel OR TI acupotomology OR AB acupotomology OR TI miniscalpel OR AB miniscalpel OR TI sword-like needle OR AB sword-like needle |  | |  |
|  | #9 | TI catgut OR AB catgut OR TI acupoint catgut embedding OR AB acupoint catgut embedding OR TI catgut implantation OR AB catgut implantation OR TI catgut embedding OR AB catgut embedding OR TI thread embedding OR AB thread embedding OR thread implantation OR thread implantation |  | |  |
|  | #10 | TI Laser acupuncture OR AB Laser acupuncture OR TI Laser puncture OR AB Laser puncture OR TI laser needle OR AB laser needle OR TI laserpuncture OR AB laserpuncture |  | |  |
|  | #11 | TI Randomized Controlled Trial OR AB Randomized Controlled Trial OR TI Controlled Clinical Trial OR AB Controlled Clinical Trial OR TI clinical trial OR AB clinical trial OR TI random OR AB random OR randomized OR randomized OR placebo OR placebo OR TI Clinical Trials as Topic OR AB Clinical Trials as Topic OR TI randomly OR AB randomly OR TI trial OR AB trial OR TI groups OR AB groups OR TI clinical research OR AB clinical research | 46702  61472* | |  |
|  | #12 | S2 OR S3 OR S4 OR S5 OR S6 OR S7 OR S8 OR S9 OR S10 | 3839  9785* | |  |
|  | #13 | S1 AND S11 AND S12 | 89  153* | |  |

* Retrieved May 8, 2024

**Supplementary Table 2.** Excluded Reports by Eligibility Assessment

| Author | Year | Title |
| --- | --- | --- |
| Inappropriate population (n = 103) | | |
| Zhang, H. M.-//-Wu, X. L.-//-Jiang, C.-//-Shi, R. X. | 2017 | Effect of Acupuncture Therapy on Body Compositions in Patients with Obesity |
| He, J.-//-Zhang, X.-//-Qu, Y.-//-Huang, H.-//-Liu, X.-//-Du, J.-//-Guo, S. | 2015 | Effect of Combined Manual Acupuncture and Massage on Body Weight and Body Mass Index Reduction in Obese and Overweight Women: a Randomized, Short-term Clinical Trial |
| Nourshahi, M.-//-Ahmadizad, S.-//-Nikbakht, H.-//-Heidarnia, M. A.-//-Ernst, E. | 2009 | The effects of triple therapy (acupuncture, diet and exercise) on body weight: a randomized, clinical trial |
| Shen, E. Y.-//-Hsieh, C. L.-//-Chang, Y. H.-//-Lin, J. G. | 2009 | Observation of sympathomimetic effect of ear acupuncture stimulation for body weight reduction |
| Chen, F.-//-Wu, S.-//-Zhang, Y. | 2007 | Effect of acupoint catgut embedding on TNF-alpha and insulin resistance in simple obesity patients |
| Liang, C. M.-//-Hu, H.-//-Wang, C. X.-//-Sun, S. G.-//-Yang, W. J.-//-Pan, L. | 2016 | Randomized Controlled Clinical Trials for Acupuncture Treatment of Abdominal Obesity |
| Liang, C. M.-//-Hu, H.-//-Li, Y. Y. | 2012 | Acupuncture treatment of abdominal obesity patients by "belt vessel (Daimai) regulating method" |
| Shen, L. Y.-//-Liang, C. M.-//-Yang, W. J.-//-Pan, L.-//-Li, H.-//-Hu, H. | 2018 | Acupuncture Treatment of Polycystic Ovarian Syndrome Patients with Abdominal Obesity by Regulating Dai Meridian: a Randomized Controlled Clinical Trial |
| Zhang, C. Y.-//-Yang, L. | 2015 | Effect of Acupuncture Therapy on Visceral Fat Thickness in Simple Central Obesity Patients |
| Bai, Y. P.-//-Fu, J. Y. | 2007 | Clinical observation on the regularity of acupuncture-induced body-reduction in excess-heat-type obesity patients |
| Zhang, H. M.-//-Jiang, C.-//-Cheng, D. X.-//-Shi, R. X. | 2017 | Effect of Acupuncture Therapy in Combination with Nutrition Control on Body-weight Reduction in Postpartum Obesity Subjects |
| Wang, J. J.-//-Huang, W.-//-Wei, D.-//-Yang, T. Y.-//-Zhou, Z. Y. | 2019 | Comparison of therapeutic effects of electroacupuncture and acupoint catgut embedding in redu-cing serum leptin and insulin levels in simple obesity patients |
| Huang, L. C.-//-Pan, W. Y. | 2011 | Comparation of effect and cost-benefit analysis between acupoint catgut-embedding and electroacupuncture on simple obesity |
| Wang, Y. J.-//-Jiao, S. L. | 2015 | Observations on the therapeutic effect of acupoint catgut embedding on the early stage of type 2 diabetes |
| Dong, C.-//-Zhang, C. R.-//-Xue, B. Y.-//-Miu, W. F.-//-Fang, N. Y.-//-Li, K.-//-Ou, Z. J.-//-Xu, Y. Q. | 2020 | Electroacupuncture combined with lifestyle control on obese nonalcoholic fatty liver disease: a randomized controlled trial |
| ChiCtr | 2021 | Effect of Real and Sham Acupoint Catgut Embedding on Psoriasis Vulgaris With Overweight/Obesity: A Pilot Study of Randomized Controlled Trial |
| Deng, L. J.-//-Lun, Z. J.-//-Ma, X. W.-//-Zhou, J. L. | 2014 | Clinical observation on regulating the three energizer by acupoint catgut embedding combined with abdominal acupuncture in treating abdominal obesity: a randomized controlled trial |
| Yang, Y.-//-Liu, Y. | 2015 | BO's abdominal acupuncture for obese type-2 diabetes mellitus |
| Yeo, S.-//-Kim, K. S.-//-Lim, S. | 2014 | Randomised clinical trial of five ear acupuncture points for the treatment of overweight people |
| Jeon, J. H.-//-Yoon, J.-//-Cho, C. K.-//-Jung, I. C.-//-Kim, S.-//-Lee, S. H.-//-Yoo, H. S. | 2015 | Effect of acupuncture for radioactive-iodine-induced anorexia in thyroid cancer patients: a randomized, double-blinded, sham-controlled pilot study |
| Li, L.-//-Zheng, S. Z.-//-Wang, H. Y.-//-Fan, E.-//-Zhu, Y. P.-//-Wei, Q. L. | 2016 | Acupoint thread embedding for prehypertension due to phlegm-dampness: a randomized controlled trial |
| Tang, Z. Y.-//-Sun, W. S.-//-Zhang, X. | 2016 | Observations on the Efficacy of Acupoint PLGA Thread Embedding in Treating Simple Obesity |
| Nct | 2014 | Effectiveness and Safety of Acupuncture for Obesity and Over-weight People |
| Lam, N. C.-//-Petersen, T. R.-//-Gerstein, N. S.-//-Yen, T.-//-Starr, B.-//-Mariano, E. R. | 2014 | A randomized clinical trial comparing the effectiveness of ultrasound guidance versus nerve stimulation for lateral popliteal-sciatic nerve blocks in obese patients |
| Li, Z. X.-//-Xie, Y. L.-//-Yi, W.-//-Zhang, H. H.-//-Tang, X. R.-//-Liu, X. X.-//-Xu, N. G. | 2019 | Clinical therapeutic effect of hyperlipidemia of turbid phlegm obstruction pattern/syndrome treated with the different Jin's three-needle therapies |
| Xu, Z.-//-Li, R.-//-Zhu, C.-//-Li, M. | 2013 | Effect of acupuncture treatment for weight loss on gut flora in patients with simple obesity |
| Cao, Y.-//-Chen, H.-//-Zhao, D.-//-Zhang, L.-//-Yu, X.-//-Zhou, X.-//-Liu, Z. | 2019 | The efficacy of Tung's acupuncture for sex hormones in polycystic ovary syndrome: a randomized controlled trial |
| Stener-Victorin, E.-//-Jedel, E.-//-Janson, P. O.-//-Sverrisdottir, Y. B. | 2009 | Low-frequency electroacupuncture and physical exercise decrease high muscle sympathetic nerve activity in polycystic ovary syndrome |
| Ippoliti, F.-//-Liguori, A.-//-Petti, F.-//-Canitano, N.-//-Rughini, S. | 2008 | Leptin, ghrelin and TNF-alpha before and after hypo-caloric traditional Chinese diet and auricular acupuncture |
| Mohamed, A. R.-//-Shaban, M. M. | 2014 | Role of laser acupuncture in chronic respiratory diseases |
| Zhao, Z. M.-//-Liu, C. L.-//-Zhang, Q. Y.-//-Zhang, B. B.-//-Guo, J. H.-//-Yuan, A. H.-//-Cai, H. | 2018 | Acupuncture Treatment Reduces Body Weight Possibly by Down-regulating Insulin and Leptin Resistance, and Up-regulating Soluble Leptin Receptor Level in Prediabetic Patients |
| Oztas, D.-//-Erdogan, S.-//-Koroglu, F. T.-//-Mollahaliloglu, S.-//-Erel, O. | 2018 | Useful Influence of Ear & Body Acupuncture Treatment on the Significant Reduction of Body Weight of Obesity and its Relationship with Oxidant-Antioxidant System |
| Abdi, H.-//-Ghayour, M. | 2015 | Effects of ear acupuncture on weight loss and serum levels of total cholesterol and triglyceride |
| Cabioglu, M. T.-//-Gundogan, N.-//-Ergene, N. | 2008 | The efficacy of electroacupuncture therapy for weight loss changes plasma lipoprotein A, Apolipoprotein A and Apolipoprotein B levels in obese women |
| Luo, H. L.-//-Li, R. H. | 2007 | Effect of electroacupuncture on leptin and adiponectin in simple obesity patients |
| Actrn | 2018 | Acupuncture or ear acupuncture for weight loss in Polycystic Ovary Syndrome |
| Jin, C. L.-//-Wei, L. X.-//-Zhao, J. P.-//-Wu, Z. C. | 2014 | Efficacy comparison between electroacupuncture and dyne-35 in treatment of polycystic ovary syndrome |
| Meng, H.-//-Hao, J.-//-Wang, H.-//-Zhao, J.-//-Zhao, C.-//-Zhai, X.-//-Sun, L.-//-Li, J. | 2014 | Effects of different frequencies of electroacupuncture on blood glucose tolerance patients |
| Wang, W. H.-//-Yu, Z. F.-//-Wang, H. L.-//-Song, G. M. | 2016 | Effects of replenishing qi for invigorating the spleen acupuncture on serum leptin of hyperuricemia population |
| Hu, Z. H.-//-Wang, Y.-//-Zhang, J. J.-//-Zhang, A. J.-//-Cao, Y.-//-Chen, L. J.-//-Lu, Q.-//-Wang, S. S.-//-Hong, M. H. | 2016 | Analysis of short- and long-term therapeutic efficacies of acupuncture for metabolic syndrome |
| Dong, H.-//-Wang, Q.-//-Cheng, L.-//-Wang, Z.-//-Wu, X.-//-Zhou, Z.-//-Yang, L.-//-Huang, D. | 2022 | Effect of Low-Frequency Electro-Acupuncture in Unmarried Women With Polycystic Ovary Syndrome: a Randomized Controlled Study |
| Dong, H. X.-//-Wang, Q.-//-Wang, Z.-//-Wu, X. K.-//-Cheng, L.-//-Zhou, Z. M.-//-Yang, L.-//-Yi, P.-//-Huang, D. M. | 2021 | Impact of Low Frequency Electro-acupuncture on Glucose and Lipid Metabolism in Unmarried PCOS Women: A Randomized Controlled Trial |
| Zhang, H. L.-//-Huo, Z. J.-//-Wang, H. N.-//-Wang, W.-//-Chang, C. Q.-//-Shi, L.-//-Li, D.-//-Li, R.-//-Qiao, J. | 2020 | Acupuncture ameliorates negative emotion in PCOS patients: a randomized controlled trial |
| Cao, Y.-//-Zhang, L.-//-Zhao, D.-//-Liu, Z. | 2017 | DONG's extraordinary acupoints for the ovarian function of polycystic ovary syndrome: a randomized controlled pilot trial |
| Yang, D.-//-Zhao, M.-//-Tan, J. | 2017 | Effect of polycystic ovary syndrome treated with the periodic therapy of acupuncture |
| Yang, B.-//-Zhang, C.-//-Yang, J.-//-Chen, C. | 2006 | Observation and mechanism exploration of acupuncture on body weight reduction |
| Kct | 2011 | Aricular acupuncture for pre & stage 1 hypertension patients: clinical trial |
| Park, K. S.-//-Gang, W.-//-Kim, P. W.-//-Yang, C.-//-Jun, P.-//-Jung, S. Y.-//-Kwon, O.-//-Lee, J. M.-//-Lee, H. J.-//-Lee, S. J.-//-et al., | 2022 | Efficacy and safety of acupuncture on oligomenorrhea due to polycystic ovary syndrome: an international multicenter, pilot randomized controlled trial |
| Avis, N. E.-//-Legault, C.-//-Coeytaux, R. R.-//-Pian-Smith, M.-//-Shifren, J. L.-//-Chen, W.-//-Valaskatgis, P. | 2008 | A randomized, controlled pilot study of acupuncture treatment for menopausal hot flashes |
| Brinkhaus, B. | 2009 | Effectiveness of diet and exercise vs. acupuncture for weight loss in obese women: results of a pilot study - Commentary |
| Irct20140907019073N | 2021 | Comparison of the efficacy of platelet rich plasma injection which extracted by two methods and placebo on pain, range of motion and function of patients with osteoarthritis of the knee |
| Irct20211016052782N | 2021 | The effect of platelet rich plasma on MRI findings among patients with knee osteoarthritis |
| Yang, B.-//-Zhang, C.-//-Yang, J.-//-Chen, C. | 2007 | Observation and Mechanism Exploration of Acupuncture on Body Weight Reduction |
| Jiao, N. | 2012 | Three sub-raise the cloudy acupuncture Point to stick on treats the hypertension clinical observation |
| You, W. K.-//-Lee, M. J.-//-Oh, J. G. | 2000 | The effects of auricular acupuncture for obesity on the change of hormone and energy metabolism during weight control of veteran Taekwondo players |
|  | 2015 | The effects of auricular acupuncture on weight reduction and feeding-related cytokines: a pilot study |
| Nct | 2020 | Leptin Infusion and Endothelial Vasomotor Response |
| Drks | 2022 | EFFECTS OF ECCENTRIC, CONCENTRIC AND ECCENTRIC/CONCENTRIC TRAINING ON MUSCLE FUNCTION AND MASS, FUNCTIONAL PERFORMANCE,CARDIOMETABOLIC HEALTH, QUALITY OF LIFE AND MOLECULAR ADAPTATIONS OF SKELETAL MUSCLE IN COPD PATIENTS: A MULTICENTER RANDOMIZED TRIAL |
| Nct | 2010 | Multi-Port Versus Single-port Cholecystectomy |
| Nct | 2022 | Efficacy Study of Acupuncture on Asymptomatic Hyperuricemia |
| Irct20191003044963N | 2020 | Comparison of supra-laryngeal nerve block by sonography guided rout and conventional rout |
| Suzuki, M.-//-Fukui, M.-//-Shiota, T.-//-Endo, K.-//-Sato, S.-//-Aihara, K.-//-Matsumoto, M.-//-Suzuki, S.-//-Itotani, R.-//-Ishitoko, M.-//-et al., | 2011 | Acupuncture improves nutritional status and BODE index in patients with chronic obstructive pulmonary disease: a randomized, placebo-controlled trial |
| Burd, N. A.-//-Yang, Y.-//-Moore, D. R.-//-Tang, J. E.-//-Tarnopolsky, M. A.-//-Phillips, S. M. | 2012 | Greater stimulation of myofibrillar protein synthesis with ingestion of whey protein isolate v. micellar casein at rest and after resistance exercise in elderly men |
| Xu, C. W.-//-Liu, H.-//-Li, M.-//-Zhao, D. | 2015 | Effect of acupuncture and exercise rehabilitationon on motor function and activity of daily life among hemiplegia patients after stroke |
| Zhang, Z. M.-//-Feng, C. L.-//-Pi, Z. K.-//-Fan, X. Y.-//-Chen, H. Q.-//-Zhang, J. | 2008 | Observation on clinical therapeutic effect of acupuncture on upper limb spasticity in the patient of poststroke |
| Leatherman, M. L.-//-Held, J. M.-//-Fluke, L. M.-//-McEvoy, C. S.-//-Inaba, K.-//-Grabo, D.-//-Martin, M. J.-//-Earley, A. S.-//-Ricca, R. L.-//-Polk, T. M. | 2017 | Relative device stability of anterior vs. axillary needle decompression for tension pneumothorax during casualty movement: preliminary analysis of a human cadaver model |
| Sang, P.-//-Zhao, J.-//-Wang, S.-//-Yang, H.-//-Shi, H. | 2020 | Influence of acupuncture on patients with post-stroke depression and through p11/tPA/BDNF pathway genes |
| Kalu, C.-//-Woelke, S.-//-Zhang, J.-//-Belury, M.-//-Shen, R.-//-Clinton, S. K.-//-Yee, L. | 2019 | Omega-3 fatty acids and ERPR(-) and HER2/neu(+/-) breast cancer prevention |
| Mora, S.-//-Robertson, C.-//-Guerrieri, G. A.-//-Cazzaniga, M.-//-Johansson, H.-//-Serrano, D.-//-Gulisano, M.-//-Cassano, E.-//-Franchi, D.-//-Decensi, A. | 2004 | A randomized 2 x 2 biomarker trial of low-dose tamoxifen and fenretinide in premenopausal women at high risk for breast cancer |
| Decensi, A.-//-Bonanni, B.-//-Guerrieri, G. A.-//-Robertson, C.-//-Cazzaniga, M.-//-Mariette, F.-//-Gulisano, M.-//-Latronico, A.-//-Franchi, D.-//-Johnson, K. | 2004 | A randomized 2x2 biomarker trial of low-dose tamoxifen and fenretinide in postmenopausal women at high-risk for breast cancer |
| Wang, HongYan-//-Zhang, YingQuan-//-Bi, Zhen | 2009 | Clinical research of central obesity treated with acupuncture, cupping and auricular therapy |
| Wen, Q.-//-Hu, M.-//-Lai, M.-//-Li, J.-//-Hu, Z.-//-Quan, K.-//-Liu, J.-//-Liu, H.-//-Meng, Y.-//-Wang, S.-//-et al., | 2022 | Effect of acupuncture and metformin on insulin sensitivity in women with polycystic ovary syndrome and insulin resistance: a three-armed randomized controlled trial |
| Ching, H. Y.-//-Wu, S. L.-//-Chen, W. C.-//-Hsieh, C. L. | 2012 | Effects of auricular acupressure on body weight parameters in patients with chronic schizophrenia |
| von Deneen, K. M.-//-Wei, Q.-//-Peng, L.-//-Hao, D. M.-//-Peng, C.-//-Xie, H. | 2011 | Randomized fMRI trial of the central effects of acute acupuncture on glucose levels and core body temperature in "overweight" males |
| Chien, L. W.-//-Lin, M. H.-//-Chung, H. Y.-//-Liu, C. F. | 2010 | Electro-acupuncture as an adjunct treatment for obesity? |
| Deng, Y. J.-//-Liu, W. Y.-//-Ouyang, L. | 2003 | Comparative observation of the effect of electroacupuncture and body acupuncture in treating simple obesity |
| Liang, C. M.-//-Wang, X. M.-//-Sun, S. G.-//-Hu, H. | 2019 | A clinical study on medical cupping for metabolic syndrome with abdominal obesity |
| Zhang, L.-//-Li, D. S.-//-Sheng, L. | 2003 | [Observations on the Effect of Magnetic Needle Treatment on Simple Obesity Complicated with Hyperlipidemia] (in Chinese) |
| Eich, H.-//-Hannig, M.-//-Zimmermann, E.-//-Klieser, E. | 2005 | Acupuncture in the treatment of psychoactive-drug-induced obesity - an experimental study |
| Lei, Y. | 2006 | Acupuncture,cupping,ear pressing,and herbs in treatment of simple obesity |
| Dai, J. Y.-//-Shao, J.-//-Wang, Y. H.-//-Wang, L.-//-Yin, X. Z. | 2006 | Clinical comparative observations on acupuncture theatment of 200 simple obesity by syndrome differentiation |
| Xiong, F. L.-//-Cui, J.-//-Ning, Y. | 2005 | [Clinical Efficacy of Lumbo-abdorninal Group Acupuncture plus Remote Point Selection for Treating Simple Obesity and the Study on Its Mechanism of Molecular Biologic Action] (in Chinese) |
| Formenti, P.-//-Galimberti, A.-//-Pinciroli, R.-//-Umbrello, M. | 2022 | Effect of Acupuncture on Diaphragm Function in Healthy Volunteers: A Pilot Clinical Study |
| Kerscher, M.-//-Wagner-Schiffler, S.-//-Drabik, A.-//-Kaptan, T. | 2018 | Cell-free autologous conditioned serum (ACS) significantly increases skin elasticity, and combination with hyaluronic acid (HA) shows no additive effects: Results of the OrthoSkin 2 clinical study |
| Masala, S.-//-Marsico, S. | 2017 | Intra-articular injections |
| Han, M.-//-Sun, Y.-//-Su, W.-//-Huang, S.-//-Li, S.-//-Gao, M.-//-Wang, W.-//-Wang, F.-//-Fang, Z.-//-Zhao, H. | 2017 | The Efficacy of Acupuncture on Anthropometric Measures and the Biochemical Markers for Metabolic Syndrome: A Randomized Controlled Pilot Study |
| Andrews, R. | 2016 | The brain-computer interface: Nano-hardware and clever software keep CARS on track |
| Bohua, Y.-//-Qusi, P.-//-Qihua, W.-//-fen, F. | 2015 | Exceptional data in paper on "The effect of meridian massage on BM, BMI, WC and HC in simple obesity patients: A randomized controlled trial": Response to the readers' letter |
| Kawachiya, S.-//-Bodri, D.-//-Matsumoto, T.-//-Kato, K.-//-Osada, H.-//-Takehara, Y.-//-Kato, O. | 2011 | Short-term, low-dose, post-trigger non-steroidal anti-inflammatory drug application improves the outcome of natural cycle IVF |
| Rahsepar, A. A.-//-Tavallaie, S.-//-Abdi, H.-//-Zhao, B.-//-Abbasi, P.-//-Nemati, M.-//-Safarian, M.-//-Mohammad, S.-//-Mohammadi, R. P. M.-//-Darbandi, S.-//-Darbandi, M.-//-Ghayour-Mobarhan, M.-//-Ferns, G. | 2011 | Effects of body acupuncture versus auricular acupuncture on anthropometric, lipid profile, inflammatory and immunologic markers: A randomized controlled trial study |
| Gibb, F. W.-//-McInnes, K. J.-//-Andrew, R.-//-Walker, B. R. | 2011 | Aromatase inhibition in healthy men induces insulin resistance, elevated blood pressure, and altered plasma lipids, with limited changes in transcript levels in subcutaneous adipose tissue |
| Sverrisdottir, Y.-//-Jedel, E.-//-Janson, P. O.-//-Stener-Victorin, E. | 2009 | Low-frequency electro-acupuncture and physical exercise decrease high muscle sympathetic nerve activity in polycystic ovary syndrome |
| Yu, A. S.-//-Yang, J. S.-//-Wei, L. X.-//-Xie, Y. Y. | 2005 | Observation on therapeutic effect of simple obesity treated with acupuncture, auricular point sticking and TDP |
| Shi, Y.-//-Zhang, L. S.-//-Zhao, C.-//-Zuo, X. Y. | 2005 | Controlled study of needle warming therapy and electroacupuncture on simple obesity of spleen deficiency type |
| Stener-Victorin, E.-//-Waldenström, U.-//-Tägnfors, U.-//-Lundeberg, T.-//-Lindstedt, G.-//-Janson, P. O. | 2000 | Effects of electro-acupuncture on anovulation in women with polycystic ovary syndrome |
| El-Shamy, F. F.-//-El-Kholy, S. S.-//-El-Rahman, M. M. A. | 2018 | Effectiveness of Laser Acupoints on Women With Polycystic Ovarian Syndrome: A Randomized Controlled Trial |
| Zhang, J.-//-Marquina, N.-//-Oxinos, G.-//-Sau, A.-//-Ng, D. | 2008 | Effect of laser acupoint treatment on blood pressure and body weight-a pilot study |
| Mi, Y. Q. | 2005 | [Clinical study on acupuncture for treatment of 80 cases of simple obesity] |
| X. Chen, W. Huang, D. Wei, J.-P. Zhao, W. Zhang, D.-G. Ding, et al. | 2022 | Effect of Acupoint Catgut Embedding for Middle-Aged Obesity: A Multicentre, Randomised, Sham-Controlled Trial |
| I. G. Lima, J. Silva, A. G. D. Silva, A. S. Andrade and L. Sousa | 2022 | Electroacupuncture reduces weight, skinfold thickness and waist circumference and increases skin temperature of the abdominal region in women: a randomized controlled trial |
| Y. Liu, Y. Zhu, L. Jiang, C. Lu, L. Xiao, T. Wang, et al. | 2022 | Efficacy of electro-acupuncture in postpartum with diastasis recti abdominis: a randomized controlled clinical tria |
| X. Zeng, Y. Li, L. Lu, H. Wen, G. Wang and C. Zuo | 2022 | A randomized controlled clinical study on Zuo's acupuncture treatment for prediabetes |
| X. Zhang, Q. Li, R. Yi, C. Xing, Y. Jin, J. Meng, et al. | 2022 | Effect of catgut embedding at acupoints versus non-acupoints in abdominal obesity: a randomized clinical trial |
| Inappropriate intervention (n = 27) | | |
| Darbandi, M.-//-Darbandi, S.-//-Mobarhan, M. G.-//-Owji, A. A.-//-Zhao, B.-//-Iraji, K.-//-Abdi, H.-//-Saberfiroozi, M.-//-Nematy, M.-//-Safarian, M.-//-et al., | 2012 | Effects of auricular acupressure combined with low-calorie diet on the leptin hormone in obese and overweight Iranian individuals |
| He, L.-//-Gao, X. L.-//-Deng, H. X.-//-Zhao, Y. X. | 2008 | Effects of acupuncture on body mass index and waist-hip ratio in the patient of simple obesity |
| Ge, W.-//-Ouyang, G.-//-Gu, X. M. | 2013 | Effects of acupoint catgut-embedding therapy combined with infrared sauna room in patients with simple obesity |
| Liang, J.-//-Feng, Z.-//-Feng, S.-//-Bao, S.-//-Wang, K. | 2018 | Skin needle embedding for obese impaired glucose tolerance |
| Xie, X. C.-//-Cao, Y. Q.-//-Gao, Q.-//-Wang, C.-//-Li, M.-//-Wei, S. G. | 2017 | Acupuncture Improves Intestinal Absorption of Iron in Iron-deficient Obese Patients: a Randomized Controlled Preliminary Trial |
| Li, W. Q.-//-Jiang, W.-//-Liu, J. | 2018 | Treatment of Simple Obesity Patients with Phlegm Dampness Stagnation Syndrome with Warming Needle Moxibustion |
| Yin, L. L.-//-Li, Y. H.-//-Wang, S. X. | 2008 | Observation on therapeutic effect of acupoint sticking therapy on simple obesity |
| Zhou, X.-//-Xing, B.-//-He, G.-//-Lyu, X.-//-Zeng, Y. | 2018 | The Effects of Electrical Acupuncture and Essential Amino Acid Supplementation on Sarcopenic Obesity in Male Older Adults: a Randomized Control Study |
| Zhang, F. H. | 2010 | Auricular point sticking and the combined therapy of auricular point sticking and body acupuncture for weight gain in 100 cases |
| Tao, S. A.-//-Bing, W. | 2015 | Clinical study on the effect of electroacupuncture on topical weight loss in simple obesity patients based on the analysis of human body components |
| Qiu, X. L.-//-Li, D. Q. | 2014 | Analysis on adipose improvement after acupuncture treating simple obesity |
| Sahebkar-Khorasani, M.-//-Safarian, M.-//-Jarahi, L.-//-Yousefi, M.-//-Salari, R.-//-Meshkat, M.-//-Ayati, M. H.-//-Bahrami-Taghanaki, H.-//-Kargozar, R.-//-Azizi, H. | 2022 | Comparative effectiveness of Hypericum perforatum, acupuncture, and lifestyle modification in the management of obesity: a randomized clinical trial |
| Lee, J. H. | 2005 | The effect of combined treatment with ultrasound and electro-lipolysis acupuncture on the change of figure in obese women |
| Su, XiaoLin-//-Zhang, RiLin-//-Wu, Yun | 2007 | Observation on therapeutic effect of acupuncture on obesity patients |
| Tong, Shu-Xian | 1994 | Treatment of obesity by integrating needling, cupping and magnetic therapy : a report of 356 cases |
| Bo, X. | 2003 | Dr. Guan Zun-Hui's clinical experience in treating simple obesity by acupuncture |
| Xu, J.-//-Ma, Q. | 2012 | Acupuncture combined with cupping therapy in the treatment of 50 cases of simple obesity |
| Zhang, X. X.-//-Tang, X. D.-//-Li, W. H. | 2012 | Twenty-eight cases of simple obesity of spleen and kidney yang deficiency pattern/syndrome in females treated with electroacupuncture and isolated-medicinal moxibustion |
| Wang, S. X.-//-Liang, R. N.-//-Xie, P. E.-//-Zhang, S. P. | 2005 | Randomized controlled observation of acupuncture and auricular-point tapping and pressing therapy in the treatment of simple obesity |
| Wang, S.-//-Liang, R.-//-Xie, P.-//-Zhang, S.-//-To, P. | 2007 | A clinically controlled study on the effect of acupuncture and auricular-point tapping and pressing therapy for the treatment of obesity |
| El-Shamy, F. F.-//-El-Kholy, S. S.-//-Labib, M.-//-Kabel, A. M. | 2019 | Ameliorative potential of acupressure on gestational diabetes mellitus: A randomized controlled trial |
| Qin, W.-//-Zhao, K.-//-Yang, H. | 2016 | Effect of acupoint catgut embedding therapy combined with Chinese medicine for nourishing the kidneys and promoting blood circulation and improving blood glucose and lipid levels as well as the pregnancy rate in obese PCOS patients with infertility |
| Yeh, M. L.-//-Chu, N. F.-//-Hsu, M. Y. F.-//-Hsu, C. C.-//-Chung, Y. C. | 2015 | Acupoint Stimulation on Weight Reduction for Obesity: A Randomized Sham-Controlled Study |
| Beyazit, Y.-//-Kekilli, M.-//-Purnak, T.-//-Spiegel, J. | 2011 | Ear stapling application as an alternative treatment for weight loss |
| Chen, M.-//-Xu, B.-//-Li, Y. T. | 2005 | Clinical study on electroacupuncture combined with manual acupuncture for treatment of simple obesity |
| Shi, Y.-//-Zhang, L. S.-//-Zhao, C.-//-He, C. Q. | 2006 | [Comparison of therapeutic effects of acupuncture-cupping plus acupoint catgut embedding and electroacupuncture on simple obesity of stomach and intestine excess-heat type] |
| Weng, C. S.-//-Hung, Y. L.-//-Shyu, L. Y.-//-Chang, Y. H. | 2004 | A study of electrical conductance of meridian in the obese during weight reduction |
| Inappropriate control group (n = 6) | | |
| Hsu, C. H.-//-Hwang, K. C.-//-Chao, C. L.-//-Lin, J. G.-//-Kao, S. T.-//-Chou, P. | 2005 | Effects of electroacupuncture in reducing weight and waist circumference in obese women: a randomized crossover trial |
| Nct | 2013 | The Effect Evaluation of Laser Acupuncture in Obesity |
| Buevich, V.-//-Bozhko, A.-//-Vtorova, L.-//-Fedorov, A. | 2010 | Acupuncture and psychotherapy in the complex treatment of obesity |
| Liang, Qiu-Hu | 1997 | Acupuncture,cupping,ear pressing,and herbs in treatment of simple obesity |
| Ismail, L. A. A.-//-Mohamed, N. A. E. G.-//-Kamel, S. A.-//-Helm, G. A.-//-Labib, L. M.-//-El-Din, A. S.-//-Salama, I. I. | 2016 | Effect of body acupuncture on anthropometric parameters, lipid profile, inflammatory markers and adipokines among obese adults |
| Lee, M. S.-//-Hwan Kim, J.-//-Lim, H. J.-//-Shin, B. C. | 2006 | Effects of abdominal electroacupuncture on parameters related to obesity in obese women: A pilot study |
| Inappropriate outcome (n = 45) | | |
| Schukro, R. P.-//-Heiserer, C.-//-Michalek-Sauberer, A.-//-Gleiss, A.-//-Sator-Katzenschlager, S. | 2014 | The effects of auricular electroacupuncture on obesity in female patients - A prospective randomized placebo-controlled pilot study |
| Elmahy, R. M. A.-//-Mohamed, G. S.-//-Rashed, L. A. | 2016 | Influence of laser puncture on endothelial dysfunction on hypertensive patients |
| Mazidi, M.-//-Abbasi-Parizad, P.-//-Abdi, H.-//-Zhao, B.-//-Rahsepar, A. A.-//-Tavallaie, S.-//-Parizadeh, S. M.-//-Rezaie, P.-//-Safariyan, M.-//-Nematy, M.-//-et al., | 2017 | The effect of electro-acupuncture on pro-oxidant antioxidant balance values in overweight and obese subjects: a randomized controlled trial study |
| Chen, Z. X. | 2008 | Clinical observation on acupuncture combined with diet control for treatment of simple obesity |
| tsmpp, R. B. R. | 2018 | Acupuncture associated with electrotherapy and running in the decreased localized fat |
| Liu, H.-//-Chen, S.-//-Zhong, C.-//-Ma, D.-//-Liu, M. | 2020 | Effect of acupuncture on blood lipid level in obese patient |
| Mazidi, M.-//-Abbasi-Parizad, P.-//-Abdi, H.-//-Zhao, B.-//-Rahsepar, A. A.-//-Tavallaie, S.-//-Parizadeh, S. M.-//-Rezaie, P.-//-Safariyan, M.-//-Nematy, M.-//-et al., | 2018 | The effect of electro-acupuncture on pro-oxidant antioxidant balance values in overweight and obese subjects: a randomized controlled trial study |
| Wei, Q. L.-//-Liu, Z. C. | 2004 | Treatment of simple obesity with auricular acupuncture, body acupuncture and combination of auricular and body acupuncture |
| Wei, Qun-Li | 2002 | Comparison between auricular acupuncture, body acupuncture and combination of auricular and body acupuncture in treating simple obesity |
| Wei, Qun-Li | 2001 | Comparison of the therapeutic effect of auricular acupuncture, body acupuncture and combination of auricular plus body acupuncture for treatment of simple obesity |
| Han, Y. P.-//-Li, Y.-//-Yang, G.-//-Wang, L. S. | 2015 | Efficacy observation of electro-acupuncture on simple obesity of female patients with spleen dysfunction and dampness syndrome |
| Yu, G. H.-//-Ding, G. A.-//-Chen, G. Z.-//-Liang, S. C.-//-Jiang, F.-//-Liang, Q. X.-//-Lu, H. H. | 2005 | [Observation on therapeutic effect of electroacupuncture on obesity induced by antipsychotics] |
| Güçel, F.-//-Bahar, B.-//-Demirtas, C.-//-Mit, S.-//-Cevik, C. | 2012 | Influence of acupuncture on leptin, ghrelin, insulin and cholecystokinin in obese women: a randomised, sham-controlled preliminary trial |
| Chen, I. J.-//-Yeh, Y. H.-//-Hsu, C. H. | 2018 | Therapeutic Effect of Acupoint Catgut Embedding in Abdominally Obese Women: a Randomized, Double-Blind, Placebo-Controlled Study |
| Hung, Y. C.-//-Hung, I. L.-//-Hu, W. L.-//-Tseng, Y. J.-//-Kuo, C. E.-//-Liao, Y. N.-//-Wu, B. Y.-//-Tsai, C. C.-//-Tsai, P. Y.-//-Chen, H. P.-//-et al., | 2016 | Reduction in postpartum weight with laser acupuncture: a randomized control trial |
| Wan, H.-//-Yan, S. X.-//-Yan, Z.-//-Zhang, S. W.-//-Wang, X.-//-Zhao, M. | 2022 | Simple obesity of stomach heat and damp obstruction treated with acupoint thread embedding therapy: a randomized controlled trial |
| Chen, M.-//-Shi, X. Y.-//-Xu, B.-//-Gu, Y. H.-//-Dong, Q.-//-Xu, L. F.-//-Li, K. P.-//-Zhang, J. B.-//-Mu, Y. Y. | 2011 | Clinical observation on acupotomy for treatment of simple obesity |
| Tong, J.-//-Chen, J. X.-//-Zhang, Z. Q.-//-Liu, C. S.-//-Pan, Y.-//-Zheng, J.-//-Yao, H. | 2011 | Clinical observation on simple obesity treated by acupuncture |
| Zhang, L.-//-Ding, Y.-//-Zhang, L.-//-Sun, G.-//-Wu, Z. | 2017 | Impacts of abdominal acupuncture on lipid metabolism in olanzapine-induced obesity |
| Lai, M. H.-//-Ma, H. X.-//-Yao, H.-//-Liu, H.-//-Song, X. H.-//-Huang, W. Y.-//-Wu, X. K. | 2010 | Effect of abdominal acupuncture therapy on the endocrine and metabolism in obesity-type polycystic ovarian syndrome patients |
| Garcia-Vivas, J. M.-//-Galaviz-Hernandez, C.-//-Becerril-Chavez, F.-//-Lozano-Rodriguez, F.-//-Zamorano-Carrillo, A.-//-Lopez-Camarillo, C.-//-Marchat, L. A. | 2014 | Acupoint catgut embedding therapy with moxibustion reduces the risk of diabetes in obese women |
| Flores, M.-//-Carlin, G.-//-Ordaz, C.-//-Oropeza, L.-//-Sanchez, V.-//-Becerril, F. | 2019 | Effect of the acupoint catgut embedding therapy vs sham acupuncture in overweight and obese patients in México |
| Cai, X. B.-//-Li, Y.-//-Wang, J. L.-//-Cao, J. | 2016 | Clinical observation of electro-acupuncture and acupoint catgut-embedding therapy in the treatment of obese polycystic ovary syndrome |
| El-Bandrawy, A. M.-//-Ghareeb, H. O. | 2016 | Effect of laser puncture combined with a diet-exercise intervention on obese polycystic ovarian females |
| Ge, B. H.-//-Wang, X. Y.-//-Zhang, T.-//-An, B. Z.-//-Chen, Y. Z.-//-Liu, Y. | 2015 | Effect of acupoint thread embedding on blood lipids and insulin in simple obesity |
| Zheng, Y. H.-//-Wang, X. H.-//-Lai, M. H.-//-Yao, H.-//-Liu, H.-//-Ma, H. X. | 2013 | Effectiveness of abdominal acupuncture for patients with obesity-type polycystic ovary syndrome: a randomized controlled trial |
| Li, Y. K.-//-Yin, G. Z. | 2010 | Observation on the effect of the catgut implantation treatment for obesity hypertriglyceridemia |
| Hassan, N. E.-//-El-Masry, S. A.-//-Elshebini, S. M.-//-Al-Tohamy, M.-//-Ahmed, N. H.-//-Rasheed, E. A.-//-El-Saeed, G. S. M.-//-Hassan, N. M.-//-Zikri, E. N.-//-El Hussieny, M. S. | 2014 | Comparison of three protocols: dietary therapy and physical activity, acupuncture, or laser acupuncture in management of obese females |
| Cabioglu, M. T.-//-Ergene, N. | 2006 | Changes in levels of serum insulin, C-peptide and glucose after electroacupuncture and diet therapy in obese women |
| Darbandi, S.-//-Darbandi, M.-//-Mokarram, P.-//-Owji, A. A.-//-Ghayour Mobarhan, M. | 2013 | Effects of body electroacupuncture on plasma leptin concentrations in people with obesity and overweight in Iran: a randomized controlled trial |
| Wang, Y. L.-//-Cao, X.-//-Liu, Z. C.-//-Xu, B. | 2013 | Observation on the therapeutic effect of electroacupuncture on simple obesity of gastrointestinal heat pattern/syndrome |
| Cabioglu, M. T.-//-Ergene, N.-//-Tan, U. | 2007 | Electroacupuncture treatment of obesity with psychological symptoms |
| Abdi, H.-//-Tayefi, M.-//-Moallem, S. R.-//-Zhao, B.-//-Fayaz, M.-//-Ardabili, H. M.-//-Razavi, A. A.-//-Darbandi, M.-//-Darbandi, S.-//-Abbasi, P.-//-et al., | 2017 | Abdominal and auricular acupuncture reduces blood pressure in hypertensive patients |
| Guo, Y. | 2014 | Plasma nesfatin-1 level in obese patients after acupuncture: a randomised controlled trial |
| Darbandi, S.-//-Darbandi, M.-//-Mokarram, P.-//-Owji, A. A.-//-Zhao, B.-//-Ghayor-Mobarhan, M.-//-Abdi, H.-//-Saberfiroozi, M.-//-Nematy, M.-//-Safarian, M. | 2013 | Effects of body electroacupuncture on plasma leptin concentrations in obese and overweight people in Iran: A randomized controlled trial |
| Cabioglu, M. T.-//-Ergene, N. | 2006 | Changes in Serum Leptin and Beta Endorphin Levels with Weight Loss by Electroacupuncture and Diet Restriction in Obesity Treatment |
| Cabioglu, M. T.-//-Ergene, N. | 2005 | Electroacupuncture therapy for weight loss reduces serum total cholesterol, triglycerides, and LDL cholesterol levels in obese women |
| Mahmoud Abd El- Kader, Shehab-//-Ahmed Khalifa, Doaa | 2012 | Impact of Weight Loss on Psychological Well Being and Biochemical Modulation in Obese Patients: A Comparison of Two Treatment Protocols |
| Çelebi, Mehmet Mesut-//-Akkurt, Soner-//-Gençbay, Mualla Biçer-//-Küçük, Mustafa Öner | 2019 | The Efficacy of Auricular Acupuncture Application in the Treatment of Obesity |
| Yao, H.-//-Chen, J. X.-//-Zhang, Z. Q.-//-Pan, Y.-//-Zheng, J.-//-Tong, J. | 2012 | Effect of acupuncture therapy on appetite of obesity patients |
| Lei, H.-//-Chen, X.-//-Hu, D. G.-//-Chen, Y. T.-//-Feng, L. C.-//-Chen, Z. Y.-//-Li, F. | 2016 | Evaluate the Efficacy of Electroacupuncture Therapy on Abdominal Fat in Obese Women by Using Magnetic Resonance Imaging |
| Han, G. H.-//-Ni, G. X.-//-Sun, J. H.-//-Pei, L. X.-//-Chen, L.-//-Li, Z.-//-Li, Q. X.-//-Chen, A.-//-Jiang, Y.-//-Yu, M. | 2022 | Short-term curative effect and safety on female abdominal obesity and defecation function treated with acupoint embedding therapy at different layers under B ultrasound |
| Sebayang, R. G.-//-Aditya, C.-//-Abdurrohim, K.-//-Lauwrence, B.-//-Mihardja, H.-//-Kresnawan, T.-//-Helianthi, D. R. | 2020 | Effects of Laser Acupuncture and Dietary Intervention on Key Obesity Parameters |
| Lillingston, F.-//-Fields, P.-//-Waechter, R. | 2019 | Auricular Acupuncture Associated with Reduced Waist Circumference in Overweight Women-A Randomized Controlled Trial |
| Chen, Li-Shu-//-Li, Yue-Ying-//-Chen, Hao-//-Liu, Bo-Wen-//-Wang, Da-Wei-//-Zhao, Yong-Hua | 2019 | Polyglycolic acid sutures embedded in abdominal acupoints for treatment of simple obesity in adults: a randomized control trial |
| Non-RCTs & Duplicates (n = 17) | | |
| Wang, H. | 2002 | Observation on the therapeutic effects of acupuncture for 60 cases of simple obesity |
| Hassan, N.-//-Shokier, H.-//-Soliman, M.-//-Shalaby, S.-//-Mostafa, M. | 2014 | Management of simple obesity by using laser acupuncture and traditional Chinese acupuncture |
| Cabioglu, M. T.-//-Ergene, N.-//-Surucu, H. S.-//-Celik, H. H.-//-Findik, D. | 2007 | Serum IgG, IgA, IgM, and IgE levels after electroacupuncture and diet therapy in obese women |
| Anshul S, Kashif M, Rohit Reddy PB, Ashwin U, Arshad K. | 2021 | Erratum regarding missing Declaration of Competing Interest statements in previously published articles (World Journal of Acupuncture – Moxibustion (2019) 29(1) (28–30), (S1003525719300364), (10.1016/j.wjam.2019.04.005)) |
| Chang, H. H. | 2010 | Acupuncture not effective as a sole intervention for obesity |
| Wei, Q.-//-Liu, Z. | 2005 | Acupuncture treatment of simple obesity |
| ChiCtr | 2022 | Efficacy of Acupuncture on Central Obesity: a randomized controlled trial |
| ChiCtr | 2022 | A randomized, single-blind, parallel-controlled study on the efficacy and safety of acupuncture point embedding in the treatment of obesity |
| ChiCtr | 2022 | A randomized double-blind and placebo-controlled clinical trial of Acupoint Application on spleen deficiency and wet resistance simple obesity |
| ChiCtr | 2022 | Lu's Acupuncture Therapy for Metabolic Syndrome through Strengthening Spleen and Stomach: a Randomized Controlled Trial |
| ChiCtr | 2022 | Effect of Electroacupuncture on Patients with Abdominal Obesity Based on Human Component Analysis: a Randomized Clinical Trial |
| ChiCtr | 2022 | A Multicenter Randomized Controlled Clinical Trial of Electro-acupuncture in Patients with Metabolic Associated Fatty Liver Disease |
| ChiCtr | 2023 | A clinical trial of the effect of different depth thread-embedding therapy based on Shu-mu combined points for weight loss in overweight/obesity |
| Pactr | 2023 | Efficacy of laser acupuncture on neurophysiological parameters of median nerve in postpartum women: a randomized Controlled Clinical Trial |
| C. F. Weiniger, B. Carvalho, I. Ronel, C. Greenberger, B. Aptekman, O. Almog, et al. | 2023 | Erratum to “A randomized trial to investigate needle redirections/re-insertions using a handheld ultrasound device versus traditional palpation for spinal anesthesia in obese women undergoing cesarean delivery” [Int. J. Obstetric Anesth. 49 (2022) 103229] (International Journal of Obstetric Anesthesia (2022) 49, (S0959289X21002879), (10.1016/j.ijoa.2021.103229) |
| ChiCtr | 2024 | Optimal selection of clinical regimens for simple obesity treated with acupoint catgut embedding: a 2³factorial randomized controlled trial |
| ChiCtr | 2024 | Long needle penetration acupuncture for Central obesity of spleen deficiency and phlegm dampness type: a randomized controlled trial |
